# Consumption of tetracyclines, sulphonamides and trimethoprim, and other antibacterials in the community, European Union/European Economic Area, 1997–2017

**DOI:** 10.1093/jac/dkab177

**Published:** 2021-08-01

**Authors:** Ann Versporten, Robin Bruyndonckx, Niels Adriaenssens, Niel Hens, Dominique L Monnet, Geert Molenberghs, Herman Goossens, Klaus Weist, Samuel Coenen, the ESAC-Net study group, Reinhild Strauss, Eline Vandael, Stefana Sabtcheva, Arjana Tambić Andrašević, Isavella Kyriakidou, Jiří Vlček, Ute Wolff Sönksen, Elviira Linask, Emmi Sarvikivi, Karima Hider-Mlynarz, Hans-Peter Blank, Flora Kontopidou, Ria Benkő, Gudrun Aspelund, Ajay Oza, Filomena Fortinguerra, Ieva Rutkovska, Rolanda Valintėlienė, Marcel Bruch, Peter Zarb, Stephanie Natsch, Hege Salvesen Blix, Anna Olczak-Pieńkowska, Ana Silva, Gabriel Adrian Popescu, Tomáš Tesař, Milan Čižman, Mayte Alonso Herreras, Vendela Bergfeldt, Amelia Andrews

**Affiliations:** 1 Laboratory of Medical Microbiology, Vaccine & Infectious Disease Institute (VAXINFECTIO), University of Antwerp, Antwerp, Belgium; 2 Interuniversity Institute for Biostatistics and statistical Bioinformatics (I-BIOSTAT), Data Science Institute, Hasselt University, Hasselt, Belgium; 3 Centre for General Practice, Department of Family Medicine & Population Health (FAMPOP), University of Antwerp, Antwerp, Belgium; 4 Centre for Health Economic Research and Modelling Infectious Diseases, Vaccine & Infectious Disease Institute (VAXINFECTIO), University of Antwerp, Antwerp, Belgium; 5 Disease Programmes Unit, European Centre for Disease Prevention and Control, Stockholm, Sweden; 6 Interuniversity Institute for Biostatistics and Statistical Bioinformatics (I-BIOSTAT), Catholic University of Leuven, Leuven, Belgium

## Abstract

**Objectives:**

Data on consumption of tetracyclines, sulphonamides and trimethoprim, and other antibacterials were collected from 30 EU/European Economic Area (EEA) countries over two decades. This article reviews temporal trends, seasonal variation, presence of change-points and changes in the composition of main subgroups of tetracyclines, sulphonamides and trimethoprim and other antibacterials.

**Methods:**

For the period 1997–2017, data on consumption of tetracyclines (ATC group J01A), sulphonamides and trimethoprim (ATC group J01E), and other antibacterials (ATC group J01X) in the community and aggregated at the level of the active substance, were collected using the WHO ATC/DDD methodology (ATC/DDD index 2019). Consumption was expressed in DDD per 1000 inhabitants per day and in packages per 1000 inhabitants per day. Consumption of tetracyclines, sulphonamides and trimethoprim, and other antibacterials was analysed based on ATC-4 subgroups and presented as trends, seasonal variation, presence of change-points and compositional changes.

**Results:**

In 2017, consumption of tetracyclines, sulphonamides and trimethoprim, and other antibacterials in the community expressed in DDD per 1000 inhabitants per day varied considerably between countries. Between 1997 and 2017, consumption of tetracyclines did not change significantly, while its seasonal variation significantly decreased over time. Consumption of sulphonamides and trimethoprim significantly decreased until 2006, and its seasonal variation significantly decreased over time. The consumption of other antibacterials showed no significant change over time or in seasonal variation.

**Conclusions:**

Consumption and composition of tetracyclines, sulphonamides and trimethoprim, and other antibacterials showed wide variations between EU/EEA countries and over time. This represents an opportunity to further reduce consumption of these groups in some countries and improve the quality of their prescription.

## Introduction

This article presents data from the European Surveillance of Antimicrobial Consumption Network (ESAC-Net,^1^ formerly ESAC) on consumption of tetracyclines, sulphonamides and trimethoprim, and other antibacterials in the community (i.e. primary care sector) for 30 EU/European Economic Area (EEA) countries in 2017 (Table 1). It updates a previous ESAC study published in 2011, and in doing so it provides updated comparable and reliable information on antibiotic consumption that can aid in fighting the global problem of antimicrobial resistance.^2^ In 2017, tetracyclines, sulphonamides and trimethoprim, and other antibacterials represented 11.3%, 2.9% and 6.1% of total antibiotic consumption in the community, respectively.^3^ The objective of this study was to analyse temporal trends, seasonal variation and the presence of change-points in consumption of tetracyclines, sulphonamides and trimethoprim, and other antibacterials for the period 1997–2017 in the community as well as to analyse their composition over time.

**Table 1.** Classification of tetracyclines (J01A), sulphonamides and trimethoprim (J01E), and other antibacterials (J01X; ATC/DDD index 2019)

Tetracyclines			
J01AA01	Demeclocycline	J01AA08	Minocycline^a^
**J01AA02**	**Doxycycline** ^a^	J01AA09	*Rolitetracycline* ^b^
J01AA03	*Chlortetracycline* ^b^	J01AA10	*Penimepicycline* ^b^
**J01AA04**	**Lymecycline** ^a^	J01AA11	*Clomocycline* ^b^
J01AA05	*Metacycline*	J01AA12	Tigecycline
J01AA06	Oxytetracycline	J01AA20	*Combinations of tetracyclines* ^b^
J01AA07	Tetracycline	J01AA56	*Oxytetracycline, combinations*

Sulphonamides and trimethoprim			

Trimethoprim and derivatives		Long-acting sulphonamides	
**J01EA01**	**Trimethoprim** ^a^	J01ED01	*Sulfadimethoxine* ^b^
J01EA02	*Brodimoprim* ^b^	J01ED02	*Sulfalene* ^b^
J01EA03	*Iclaprim* ^b^	J01ED03	*Sulfametomidine* ^b^
		J01ED04	*Sulfametoxydiazine* ^b^
Short-acting sulphonamides		J01ED05	*Sulfamethoxypyridazine*
J01EB01	*Sulfaisodimidine* ^b^	J01ED06	*Sulfaperin* ^b^
J01EB02	Sulfamethizole	J01ED07	*Sulfamerazine* ^b^
J01EB03	*Sulfadimidine* ^b^	J01ED08	*Sulfaphenazole* ^b^
J01EB04	Sulfapyridine	J01ED09	*Sulfamazon* ^b^
J01EB05	Sulfafurazole^b^	J01ED20	*Combinations* ^b^
J01EB06	*Sulfanilamide* ^b^		
J01EB07	*Sulfathiazole* ^b^	Combinations of sulphonamides and trimethoprim, including derivatives	
J01EB08	*Sulfathiourea* ^b^	J01EE01	**Sulfamethoxazole and trimethoprim** ^a^
J01EB20	*Combinations* ^b^	J01EE02	Sulfadiazine and trimethoprim^b^
		J01EE03	Sulfametrole and trimethoprim
I ntermediate-acting sulphonamides		J01EE04	*Sulfamoxole and trimethoprim* ^b^
J01EC01	*Sulfamethoxazole* ^b^	J01EE05	*Sulfadimidine and trimethoprim* ^b^
J01EC02	Sulfadiazine	J01EE06	*Sulfadiazine and tetroxoprim* ^b^
J01EC03	*Sulfamoxole* ^b^	J01EE07	*Sulfamerazine and trimethoprim* ^b^
J01EC20	*Combinations* ^b^		

Other antibacterials			

Glycopeptide antibacterials		Nitrofuran derivatives	
J01XA01	Vancomycin	**J01XE01**	**Nitrofurantoin** ^a^
J01XA02	Teicoplanin	J01XE02	Nifurtoinol^a^
J01XA03	*Televancin* ^b^	J01XE03	Furazidin^c^
J01XA04	Dalbavacin^b^	J01XE51	Nitrofurantoin, combinations^c^
J01XA05	*Oritavancin* ^b^		
		Other antibacterials	
Polymyxins		**J01XX01**	**Fosfomycin**
J01XB01	Colistin	J01XX02	*Xibornol* ^b^
J01XB02	*Polymyxin B* ^b^	J01XX03	Clofoctol
		J01XX04	*Spectinomycin*
Steroid antibacterials		**J01XX05**	**Methenamine** ^a^
J01XC01	*Fusidic acid*	J01XX06	*Mandelic acid*
		J01XX07	Nitroxoline
Imidazole derivatives		J01XX08	Linezolid
J01XD01	Metronidazole^a^	J01XX09	Daptomycin
J01XD02	Tinidazole	J01XX10	*Bacitracin* ^b^
J01XD03	*Ornidazole*	J01XX11	Tedizolid^c^

**Bold type** indicates that consumption was part of the top 90% of the community consumption of tetracyclines (J01A), sulphonamides and trimethoprim (J01E), or other antibacterials (J01X) in 28 EU/EEA countries in 2017; *Italic type* indicated that no consumption of this antibiotic was reported in 28 EU/EEA countries in 2017.

a Consumption was part of the top 90% of the community consumption of tetracyclines (J01A), sulphonamides and trimethoprim (J01E), or other antibacterials (J01X) in 30 EU/EEA countries in 2009.

b No consumption of this antibiotic was reported in 30 EU/EEA countries in 2009.

c This antibiotic was not included in the ATC/DDD index in 2009 and was therefore not reported in 2009.

## Methods

The methods for collecting and analysing the data are described in the introductory article of this series.^4^ In summary, data on consumption of tetracyclines, i.e. Anatomical Therapeutic Chemical (ATC) group J01A, sulphonamides and trimethoprim (ATC group J01E), and other antibacterials (ATC group J01X) in the community and aggregated at the level of the active substance, were collected using the WHO ATC/DDD methodology (ATC/DDD index 2019^5^) and expressed in DDD per 1000 inhabitants per day. In addition, where data were available, consumption was also expressed in packages per 1000 inhabitants per day. There are 14, 33 and 26 unique ATC codes for tetracyclines, sulphonamides and trimethoprim, and other antibacterials, respectively, in the ATC/DDD index 2019. Compared with previous descriptions of the consumption of other antibacterials in the community, two additional nitrofuran derivatives, i.e. furazidin (J01XE03) and nitrofurantoin, combinations (J01XE51), and one additional other antibacterial substance, i.e. tedizolid (J01XX11), have been assigned an ATC code by the WHO (Table 1).^2^

The evolution of the number of DDD per package over time was assessed using a linear mixed model. The temporal trend, seasonal variation and presence of change-points for tetracyclines, sulphonamides and trimethoprim and other antibacterials were assessed using a non-linear change-point mixed model fitted to quarterly data expressed in DDD per 1000 inhabitants per day from 1997 to 2017.^6^ The relative proportions of the main subgroups for sulphonamides and trimethoprim, and other antibacterials were assessed through a compositional data analysis modelling yearly data expressed in DDD per 1000 inhabitants per day from 1997 to 2017.^7^

## Results

### Tetracyclines

An overview of consumption of tetracyclines (ATC J01A) in the community, expressed in DDD and packages per 1000 inhabitants per day for all participating countries between 1997 and 2017 is available as Supplementary data at *JAC* Online (Tables S1 and S2, respectively).

#### Consumption of tetracyclines in the community in 2017

In 2017, two substances accounted for 90% of consumption of tetracyclines in the community expressed in DDD per 1000 inhabitants per day: doxycycline (75.8% in 2017 compared with 74.9% in 2009) and lymecycline (14.8% in 2017 compared with 10.2% in 2009) (Table 1).

**Figure 1.**
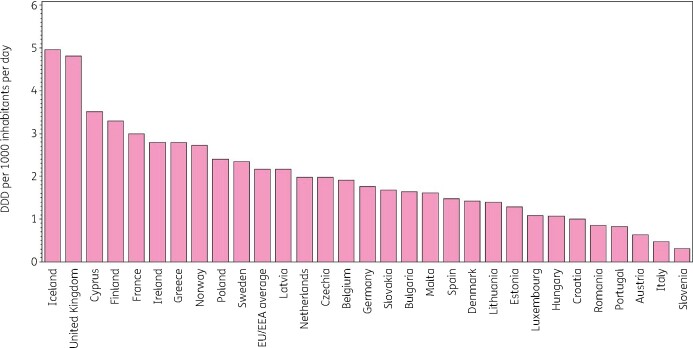
Consumption of tetracyclines (ATC J01A) in the community, expressed in DDD (ATC/DDD index 2019) per 1000 inhabitants per day, 30 EU/EEA countries, 2017. For Czechia, 2015 data are used. For Slovakia, 2016 data are used. For Cyprus and Romania, total care data, i.e. community and hospital sector combined, are used.

Figure 1 shows the consumption of tetracyclines in the community expressed in DDD per 1000 inhabitants per day in 2017, which varied by factor 16 between countries with the highest (5.0 DDD per 1000 inhabitants per day in Iceland) and the lowest (0.3 DDD per 1000 inhabitants per day in Slovenia) consumption. This was higher than in 2009 (factor of 10, from 5.1 DDD per 1000 inhabitants per day in Iceland to 0.5 DDD per 1000 inhabitants per day in Italy).

Doxycycline was the only tetracycline prescribed in Croatia, Lithuania and Slovenia and it represented >50% of consumption of tetracyclines in the community in all but four countries (Belgium, Denmark, Sweden and the United Kingdom, where its consumption represented >30%). Lymecycline was the most consumed in Sweden (51.5% of consumption of tetracyclines in the community), and represented >30% in Belgium, Norway and the United Kingdom, and >20% in Austria, Denmark, Ireland and Finland. Minocycline represented >20% of consumption of tetracyclines in the community in Belgium, Luxembourg and Malta. The substance tetracycline (J01AA07) was the most consumed in Denmark (34.6%) and represented >10% in Finland, Norway and Romania (total care data, i.e. community and hospital sector combined). Tigecycline for parenteral use was consumed in the community in six countries ranging from 0.006 DDD per 1000 inhabitants per day in Greece to 0.001 DDD per 1000 inhabitants per day in Malta.

**Figure 2.**
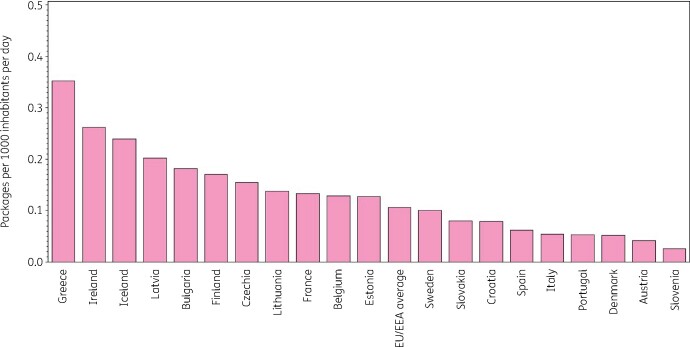
Consumption of tetracyclines (ATC J01A) in the community, expressed in packages per 1000 inhabitants per day, 20 EU/EEA countries in 2017. For Czechia, 2015 data are used. For Slovakia, 2016 data are used. For Cyprus and Romania, total care data, i.e. community and hospital sector combined, are used.

Figure 2 shows consumption of tetracyclines in the community expressed in packages per 1000 inhabitants per day for 20 EU/EEA countries in 2017. Bulgaria ranked 11th for its consumption of tetracyclines in DDD per 1000 inhabitants per day and 5th in packages per 1000 inhabitants per day, Lithuania 14th and 8th, France 3rd and 9th, Sweden 6th and 12th, and Denmark 13th and 18th, respectively (Table 2). Consequently, the lowest mean number of DDD per package was found in Bulgaria (9 DDD per package) and Lithuania (10.2 DDD per package), the highest in France (22.6 DDD per package), Sweden (23.5 DDD per package) and Denmark (27.5 DDD per package). The number of DDD per package ranged from 7.9 in Greece to 27.5 in Denmark and was higher than in 2009 (from 6.3 in Bulgaria to 22.8 in Denmark). In the EU/EEA countries, the number of DDD per package did not change significantly over time during 1997–2017.

**Table 2.** Ranking of consumption of tetracyclines (ATC J01A) in the community, expressed in DDDs or packages per 1000 inhabitants per day, 20 EU/EEA countries in 2017

Country	Greece	Ireland	Iceland	Latvia	Bulgaria	Finland	Czechia	Lithuania	France	Belgium	Estonia	Sweden	Slovakia	Croatia	Spain	Italy	Portugal	Denmark	Austria	Slovenia
Ranking for packages per 1000 inhabitants per day	1	2	3	4	5	6	7	8	9	10	11	12	13	14	15	16	17	18	19	20
Ranking for DDD per 1000 inhabitants per day	5	4	1	7	11	2	8	14	3	9	15	6	10	16	12	19	17	13	18	20
Number of DDD per package	7.9	10.7	20.7	10.7	9.0	19.3	12.8	10.2	22.6	14.9	10.1	23.5	20.8	12.6	23.6	8.8	15.6	27.5	15.3	11.8

For Czechia, 2015 data are used. For Slovakia, 2016 data are used. For Cyprus and Romania, total care data, i.e. community and hospital sector combined, are used.

#### Longitudinal data analysis, 1997–2017

**Figure 3.**
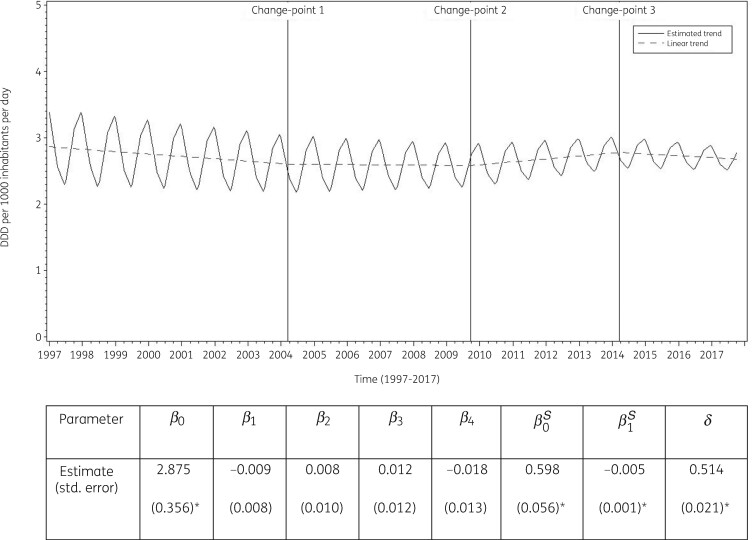
Estimated trend (solid line) and linear trend (dashed line) of consumption of tetracyclines (ATC J01A) in the community based on quarterly data, 25 EU/EEA countries, 1997–2017. *β_0_*, predicted consumption in the first quarter of 1997; *β_1_*, predicted increase (if positive)/decrease (if negative) in consumption per quarter; *β_2_*, predicted difference in slope after versus before the first change-point; *β_3_*, predicted difference in slope after versus before the second change-point; *β_4_*, predicted difference in slope after versus before the third change-point; *β_0_^S^*, predicted amplitude of the upward winter and downward summer peak in consumption; *β_1_^S^*, predicted increase (if positive)/decrease (if negative) of the amplitude of the upward winter and downward summer peak in consumption per quarter; *δ*, shift in timing of the upward winter and downward summer peak from one year to another. An asterisk indicates the result was statistically significant at significance level 0.05.

The best fit was obtained for a model including three change-points: one in the first quarter of 2004, another in the third quarter of 2009 and a third one in the first quarter of 2014. The final model fits the observed data well (Figure S1). The longitudinal data analysis estimated an average consumption of tetracyclines of 2.875 (SE 0.356) DDD per 1000 inhabitants per day in the first quarter of 1997, which did not change significantly over time: −0.009 (SE 0.008) DDD per 1000 inhabitants per day per quarter until the first quarter of 2004, then −0.001 (SE 0.011) DDD per 1000 inhabitants per day per quarter until the third quarter of 2009, then +0.011 (SE 0.016) DDD per 1000 inhabitants per day per quarter until the first quarter or 2014, and finally −0.007 (SE 0.020) DDD per 1000 inhabitants per day per quarter. A statistically significant seasonal variation was observed with an amplitude of 0.598 (SE 0.056) DDD per 1000 inhabitants per day, which decreased significantly over time by 0.005 (SE 0.001) DDD per 1000 inhabitants per day per quarter (Figure 3).

Based on the fitted model, consumption of tetracyclines in the community in 1997 was significantly higher than average in Belgium, Finland, Iceland, Latvia and Poland, and significantly lower than average in Austria, Croatia, Denmark, Italy, Portugal, Slovakia, Slovenia and Spain (observed profiles shown in Figure S2 and S3). The seasonal variation was significantly larger than average in Belgium, Finland, Iceland, the Netherlands, Poland and Sweden, and significantly smaller than average in Denmark, Greece, Ireland, Italy, Portugal, Slovenia, Spain and the United Kingdom. The decrease in consumption of tetracyclines in the community between 1997 and the first quarter of 2004 was significantly higher than average in Belgium, Finland, Hungary and Portugal. The decrease in consumption of tetracyclines between the second quarter of 2004 and the third quarter of 2009 was significantly larger than average in Czechia, Ireland, Latvia and Slovakia. The increase in consumption of tetracyclines between the fourth quarter of 2009 and the first quarter of 2014 was significantly larger than average in the United Kingdom. The decrease in consumption of tetracyclines between the second quarter of 2014 and 2017 was significantly higher than average in Finland.

Between 2009 and 2017, consumption of tetracyclines increased in 12 countries (Table S1). The largest increases were observed for Greece, Spain (including private prescriptions from 2016 onwards) and the United Kingdom, while the largest decreases were observed for Estonia, Germany and Luxembourg. The increase in consumption of tetracyclines was mainly the result of the increase in doxycycline consumption. For Malta, a substantial increase in lymecycline consumption was also observed. As in 2009, Italy had the lowest consumption of tetracyclines in 2017.

#### Compositional data analysis (1997–2017)

For the tetracyclines, grouping into subgroups is not available from the ATC classification^5^ and variation in the consumption among the different tetracycline substances was not assessed.

### Sulphonamides and trimethoprim

An overview of consumption of sulphonamides and trimethoprim (ATC J01E) in the community, expressed in DDD and packages per 1000 inhabitants per day for all participating countries between 1997 and 2017 is available as Supplementary data (Tables S3 and S4, respectively).

#### Consumption of sulphonamides and trimethoprim in 2017

In 2017, the two most used substances accounted for 90% of the consumption of sulphonamides and trimethoprim in the community expressed in DDD per 1000 inhabitants per day: sulfamethoxazole and trimethoprim (65.8% in 2017 compared with 67.7% in 2009) and trimethoprim (29.4% in 2017 compared with 29.8% in 2009) (Table 1).

**Figure 4.**
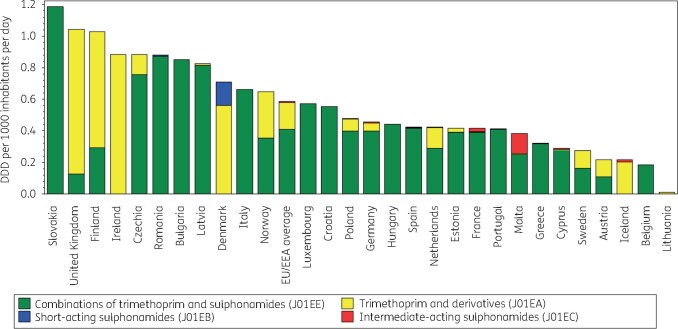
Consumption of sulphonamides and trimethoprim (ATC J01E) in the community, expressed in DDD (ATC/DDD index 2019) per 1000 inhabitants per day, 30 EU/EEA countries, 2017. For Czechia, 2015 data are used. For Slovakia, 2016 data are used. For Cyprus and Romania, total care data, i.e. community and hospital sector combined, are used.

Figure 4 shows the consumption of sulphonamides and trimethoprim in the community expressed in DDD per 1000 inhabitants per day in 2017. Consumption of sulphonamides and trimethoprim varied by a factor of 105 between countries with the highest (1.04 DDD per 1000 inhabitants per day in the United Kingdom) and the lowest (0.01 DDD per 1000 inhabitants per day in Lithuania) consumption (Table S3), which was lower than in 2009 (factor of 203, from 1.18 DDD per 1000 inhabitants per day in the United Kingdom to 0.006 DDD per 1000 inhabitants per day in Lithuania).

For six countries (Belgium, Bulgaria, Croatia, Hungary, Italy and Luxembourg), consumption of sulphonamides and trimethoprim in the community was represented by a single substance, namely sulfamethoxazole plus trimethoprim (J01EE01). In another eight countries, combinations of sulphonamides and trimethoprim, including derivatives (J01EE) represented >90% of the consumption of sulphonamides and trimethoprim in the community, and it represented >80% of this consumption in Germany and Poland. In Ireland and Lithuania, consumption of sulphonamides and trimethoprim in the community was only represented by trimethoprim (J01EA01). Trimethoprim also represented >90% of consumption of sulphonamides and trimethoprim in the community in Iceland, >80% in the United Kingdom, >70% in Denmark and Finland and >50% in Austria. Denmark was the only country consuming a substantial proportion of short-acting sulphonamides (>20%, sulfamethizole). The highest consumption of intermediate-acting sulphonamides (sulfadiazine) was found in Malta (>30%), followed by Iceland and France (>5%), and Cyprus (total care data) and Germany (>1%). Consumption of sulfadiazine was <1% in six countries and no consumption of this substance was reported for 14 countries. In 2017, no country consumed long-acting sulphonamides in the community.

**Figure 5.**
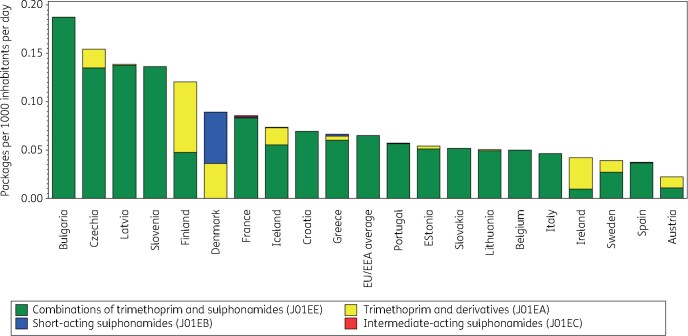
Consumption of sulphonamides and trimethoprim (ATC J01E) in the community, expressed in packages per 1000 inhabitants per day, 20 EU/EEA countries, 2017. For Czechia, 2015 data are used. For Slovakia, 2016 data are used. For Cyprus and Romania, total care data, i.e. community and hospital sector combined, are used.

Figure 5 shows the consumption of sulphonamides and trimethoprim in the community expressed in packages per 1000 inhabitants per day for 20 EU/EEA countries in 2017. In addition, country ranking is depicted according to both DDD and packages per 1000 inhabitants per day in 2017 (Table 3). The number of DDD per package ranged from 0.2 in Lithuania to 22.9 in Slovakia (2016 data). A high number of DDD per package was associated with a higher ranking of consumption expressed in DDD per 1000 inhabitants per day than in packages per 1000 inhabitants per day. Slovakia (2016 data) and Ireland, for example, had a higher ranking in DDD than in packages per 1000 inhabitants per day because of the high number of DDD per package. Conversely, Iceland and Lithuania, for example, had a lower ranking in DDD than in packages per 1000 inhabitants per day because of low number of DDD per package. In the EU/EEA countries, the number of DDD per package did not change significantly over time during 1997–2017.

**Table 3.** Ranking of consumption of sulphonamides and trimethoprim (ATC J01E) in the community, expressed in DDDs or packages per 1000 inhabitants per day, 20 EU/EEA countries, 2017

Country	Bulgaria	Czechia	Latvia	Slovenia	Finland	Denmark	France	Iceland	Croatia	Greece	Portugal	Estonia	Slovakia	Lithuania	Belgium	Italy	Ireland	Sweden	Spain	Austria
Ranking for packages per 1000 inhabitants per day	1	2	3	–	4	5	6	7	8	9	10	11	12	13	14	15	16	17	18	19
Ranking for DDD per 1000 inhabitants per day	5	4	6	–	2	7	12	17	9	14	13	11	1	19	18	8	3	15	10	16
Number of DDD per package	4.5	5.7	5.9	–	8.5	8.0	4.9	3.0	8.0	4.9	7.2	7.7	22.9	0.2	3.7	14.3	20.9	7.0	11.3	9.7

For Czechia, 2015 data are used. For Slovakia, 2016 data are used. For Cyprus and Romania, total care data, i.e. community and hospital sector combined, are used. For Slovenia, sulphonamide and trimethoprim (J01E) consumption was not reported in 2017.

#### Longitudinal data analysis, 1997–2017

**Figure 6.**
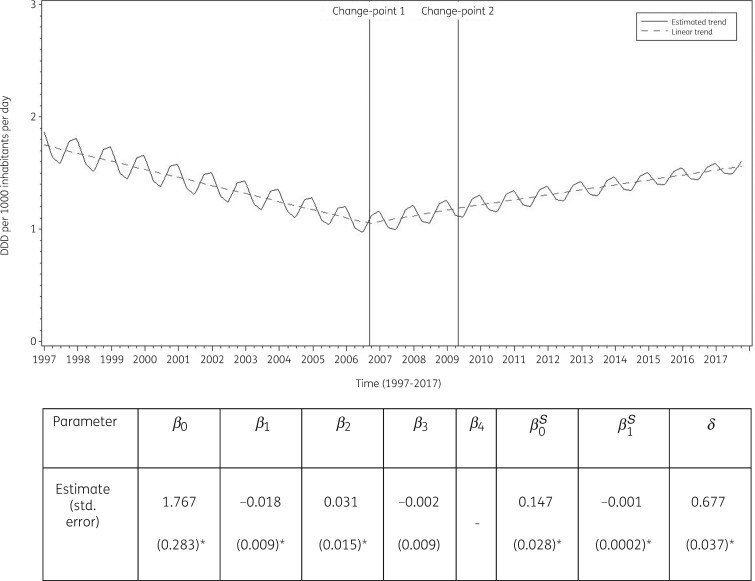
Estimated trend (solid line) and linear trend (dashed line) of consumption of sulphonamides and trimethoprim (ATC J01E) in the community expressed based on quarterly data, 25 EU/EEA countries, 1997–2017. *β_0_*, predicted consumption in the first quarter of 1997; *β_1_*, predicted increase (if positive)/decrease (if negative) in consumption per quarter; *β_2_*, predicted difference in slope after versus before the first change-point; *β_3_*, predicted difference in slope after versus before the second change-point; *β_4_*, predicted difference in slope after versus before the third change-point; *β_0_^S^*, predicted amplitude of the upward winter and downward summer peak in consumption; *β_1_^S^*, predicted increase (if positive)/decrease (if negative) of the amplitude of the upward winter and downward summer peak in consumption per quarter; *δ*, shift in timing of the upward winter and downward summer peak from one year to another. An asterisk indicates that the result was statistically significant at significance level 0.05.

The best fit was obtained for a model including two change-points: one in the third quarter of 2006 and another in the second quarter of 2009. The final model fits the observed data well (Figure S4). The longitudinal data analysis estimated an average consumption of sulphonamides and trimethoprim in the EU/EEA of 1.767 (SE 0.283) DDD per 1000 inhabitants per day in the first quarter of 1997, which significantly decreased by −0.018 (SE 0.009) DDD per 1000 inhabitant per day per quarter until the third quarter of 2006. After the first change-point, consumption of sulphonamides and trimethoprim did not change significantly; +0.013 (SE 0.015) DDD per 1000 inhabitants per day per quarter up to the second quarter of 2009, then +0.010 (SE 0.016) DDD per 1000 inhabitants per day per quarter. A statistically significant seasonal variation was observed with an amplitude of 0.147 (SE 0.028) DDD per 1000 inhabitants per day, which decreased significantly over time by 0.001 (SE 0.0002) DDD per 1000 inhabitants per day per quarter (Figure 6).

Based on the fitted model, consumption of sulphonamides and trimethoprim in the community in 1997 was significantly larger than average in Iceland, Latvia and Poland, and significantly smaller than average in Austria, Belgium, Denmark, Germany, Ireland, Italy, Luxembourg, the Netherlands, Portugal, Slovenia, Spain, Sweden and the United Kingdom (observed profiles shown in Figures S5 and S6). The seasonal variation was significantly larger than average in Czechia, Hungary, Latvia and Poland, and significantly smaller than average in Austria, Denmark, the Netherlands, Portugal, Spain, Sweden and the United Kingdom. The decrease in consumption of sulphonamides and trimethoprim in the community between 1997 and the third quarter of 2006 was significantly larger than average in Latvia and Poland. The increase in consumption of sulphonamides and trimethoprim between the last quarter of 2006 and the second quarter of 2009 was significantly larger than average in Latvia.

#### Compositional data analysis, 1997–2017

Although consumption of sulphonamides and trimethoprim in the community significantly decreased over time until 2006 and later remained stable, the proportional consumption of trimethoprim and derivatives (J01EA) significantly increased relative to that of all other subgroups of sulphonamides and trimethoprim. The proportional consumption of long-acting sulphonamides (J01ED) significantly decreased relative to that of all other subgroups of sulphonamides and trimethoprim. In addition, the proportional consumption of short-acting sulphonamides (J01EB) significantly decreased relative to that of intermediate-acting sulphonamides (J01EC) and of sulphonamides and trimethoprim, including derivatives (J01EE) (Table 4).

**Table 4.** Change in the composition of the consumption of sulphonamides and trimethoprim (ATC J01E) in the community, expressed in DDD (ATC/DDD index 2019) per 1000 inhabitants per day, 30 EU/EEA countries, as a function of time during 1997–2017

	J01EA	J01EB	J01EC	J01ED	J01EE
J01EA		**0.1194**	**0.0212**	**0.4550**	**0.0203**
J01EB	**−0.1194**		**−0.0983**	**0.3355**	**−0.0992**
J01EC	**−0.0212**	**0.0983**		**0.4338**	−0.0009
J01ED	**−0.4550**	**−0.3355**	**−0.4338**		**−0.4347**
J01EE	**−0.0203**	**0.0992**	0.0009	**0.4347**	

Values are estimated changes in the log ratio of the row versus column subgroup of antibiotics with increasing time. Bold type indicates a statistically significant effect; positive values represent an increase and negative values represent a decrease.

J01EA, trimethoprim and derivatives; J01EB, short-acting sulphonamides; J01EC, intermediate-acting sulphonamides; J01ED, long-acting sulphonamides; J01EE, sulphonamides and trimethoprim, including derivatives.

Trends of proportional consumption in individual countries are shown in Figure S7. When comparing the composition of the consumption of sulphonamides and trimethoprim in 2017 with that in 2009, we focused on countries reporting consumption in both years (i.e. all countries except Belgium and Slovenia). For the proportion of trimethoprim and derivatives (J01EA), both increases and decreases were observed. The largest increases were observed for Iceland (+40.32%), Ireland (+26.40%) and Poland (+15.82%), while the largest decreases were observed for Finland (−28.57%), Norway (−20.52%) and Sweden (−15.39%). These changes were counteracted by decreases (or increases, respectively) in the proportion of combinations of sulphonamides and trimethoprim (J01EE). The proportion of short-acting sulphonamides only changed only for Denmark (−14.50%). The proportion of intermediate-acting sulphonamides remained stable for most countries, but changes >5% were observed for Malta (+28.01%) and Iceland (+6.69%). Long-acting sulphonamides were not consumed in the community in any of the countries in 2017, while in 2009 they were still consumed in the United Kingdom (0.04%).

### Other antibacterials

An overview of consumption of other antibacterials (ATC J01X) in the community, expressed in DDD and packages per 1000 inhabitants per day for all participating countries between 1997 and 2017 is available as Supplementary data (Tables S5 and S6, respectively).

#### Consumption of other antibacterials in the community in 2017

In 2017, the three most used substances accounted for 90% of the consumption of other antibacterials in the community expressed in DDD per 1000 inhabitants per day: nitrofurantoin (58.5% in 2017 compared with 55.3% in 2009), methenamine hippurate/mandelate (22.6% in 2017 compared with 23.3% in 2009) and fosfomycin (10.6% in 2017 compared with 4.7% in 2009) (Table 1).

**Figure 7.**
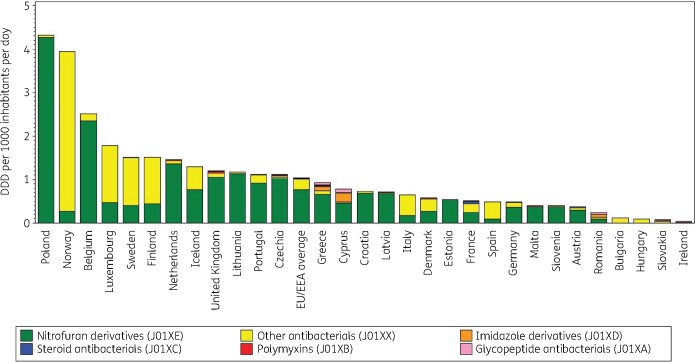
Consumption of other antibacterials (ATC J01X) in the community, expressed in DDD (ATC/DDD index 2019) per 1000 inhabitants per day, 30 EU/EEA countries in 2017. For Czechia, 2015 data are used. For Slovakia, 2016 data are used. For Cyprus and Romania, total care data, i.e. community and hospital sector combined, are used. For Ireland, nitrofurantoin (J01XE01) consumption is not included.

Figure 7 shows the consumption of other antibacterials (J01X) in the community expressed in DDD per 1000 inhabitants per day in 2017. Consumption of other antibacterials varied by a factor of 130 between countries with the highest (4.32 DDD per 1000 inhabitants per day in Poland) and the lowest [0.03 DDD per 1000 inhabitants per day in Ireland; consumption of nitrofurantoin (J01XE01) not included] consumption (Table S5). This was much lower than in 2009 (factor of 892, from 2.85 DDD per 1000 inhabitants per day in Norway to 0.003 DDD per 1000 inhabitants per day in Bulgaria).

Nitrofuran derivatives (J01XE) represented >50% of the consumption of other antibacterials in the community in 17 countries: >90% in Belgium, Croatia, Czechia (2015 data), Estonia, Latvia, Lithuania, Malta, the Netherlands, Poland and Slovenia; >80% in Portugal and the United Kingdom; >70% in Austria, Germany and Greece and >50% in Cyprus (total care data, community and hospital sector combined) and Iceland. In Spain and Norway, nitrofuran derivatives represented <20% of the consumption of other antibacterials. No consumption of this subgroup was reported for Bulgaria, Hungary and Slovakia (2016 data). In all but two countries, nitrofurantoin was the only nitrofuran derivative consumed [nifurtoinol in Belgium (24.4%) and Luxembourg (6.0%)].

The subgroup ‘other antibacterials’ (J01XX) represented >70% of the other antibacterials (J01X) in eight countries (Bulgaria, Finland, Hungary, Italy, Luxembourg, Norway, Spain and Sweden), and it represented >20% in Denmark, France, Germany, Iceland, Ireland [consumption of nitrofurantoin (J01XE01) not included] and Slovakia (2016 data). Methenamine hippurate/mandelate and fosfomycin, and to a lesser extent nitroxoline and linezolid, were also consumed, each country using a substantial proportion of only one of these substances. The highest consumption of glycopeptide antibacterials (J01XA, i.e. parenteral vancomycin and teicoplanin) was reported for Cyprus (total care data, i.e. community and hospital sector combined) followed by Greece, Romania (total care data), Czechia (2015 data) and the United Kingdom. For six countries, no consumption of glycopeptide antibacterials was reported in the community. The highest consumption of polymyxins (J01XB; only represented by parenteral colistin) in the community was observed for the United Kingdom, followed by the Netherlands, Greece and Denmark. Steroid antibacterials (J01XC; represented by fusidic acid) were consumed by 14 countries and represented 10.5% of the consumption of other antibacterials (J01X) in Ireland [consumption of nitrofurantoin (J01XE01) not included] and France. Imidazole derivatives [J01XD, represented mainly by parenteral metronidazole; and not including oral metronidazole (P01AB01)] were consumed in the community in 22 countries with the highest consumption reported in Cyprus (total care data, i.e. community and hospital sector combined), followed by Greece, Romania (total care data) and Czechia (2015 data).

**Figure 8.**
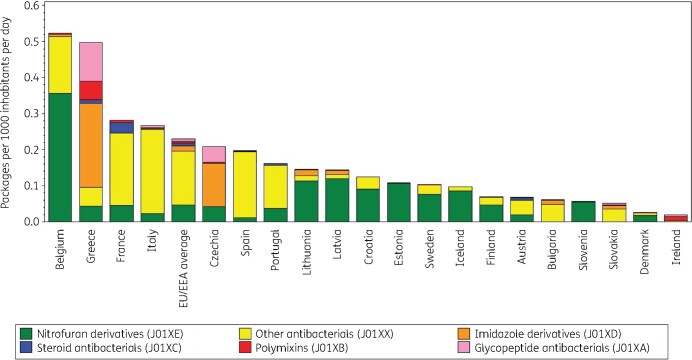
Consumption of other antibacterials (ATC J01X) in the community, expressed in packages per 1000 inhabitants per day 20 EU/EEA countries, 2017. For Czechia, 2015 data are used. For Slovakia, 2016 data are used. For Cyprus and Romania, total care data, i.e. community and hospital sector combined, are used. For Ireland, nitrofurantoin (J01XE01) consumption is not included.

Figure 8 shows consumption of other antibacterials in the community expressed in packages per 1000 inhabitants per day for 20 EU/EEA countries in 2017. The number of DDD per package ranged from 1.6 in Slovakia (2016 data) to 22.5 in Denmark. A high number of DDD per package was generally associated with a higher ranking in DDD per 1000 inhabitants per day than in packages per 1000 inhabitants per day (Table 5). Denmark and Finland had a higher ranking in DDD than in packages per 1000 inhabitants per day because of the high number of DDD per package. Inversely, Greece, France and Italy, for example, had a lower ranking in DDD than in packages per 1000 inhabitants per day because of the low number of DDD per package. In the EU/EEA countries, the number of DDD per package significantly increased over time during 1997–2017, with the steepness of this increase significantly reducing over the years.

**Table 5.** Ranking of consumption of other antibacterials (ATC J01X) in the community, expressed in DDDs or packages per 1000 inhabitants per day 20 EU/EEA countries, 2017

Country	Belgium	Greece	France	Italy	Czechia	Spain	Portugal	Lithuania	Latvia	Croatia	Estonia	Sweden	Iceland	Finland	Austria	Bulgaria	Slovenia	Slovakia	Denmark	Ireland
Ranking for packages per 1000 inhabitants per day	1	2	3	4	5	6	7	8	9	10	11	12	13	14	15	16	17	18	19	20
Ranking for DDD per 1000 inhabitants per day	1	8	14	11	7	15	6	5	10	9	13	2	4	3	17	18	16	19	12	20
Number of DDD per package	4.8	1.9	1.8	2.4	5.3	2.5	6.9	8.0	4.9	5.9	5.0	14.6	13.3	21.7	5.7	1.8	7.0	1.6	22.5	1.7

For Czechia, 2015 data are used. For Slovakia, 2016 data are used. For Cyprus and Romania, total care data, i.e. community and hospital sector combined, are used. For Ireland, nitrofurantoin (J01XE01) consumption is not included.

#### Longitudinal data analysis, 1997–2017

**Figure 9.**
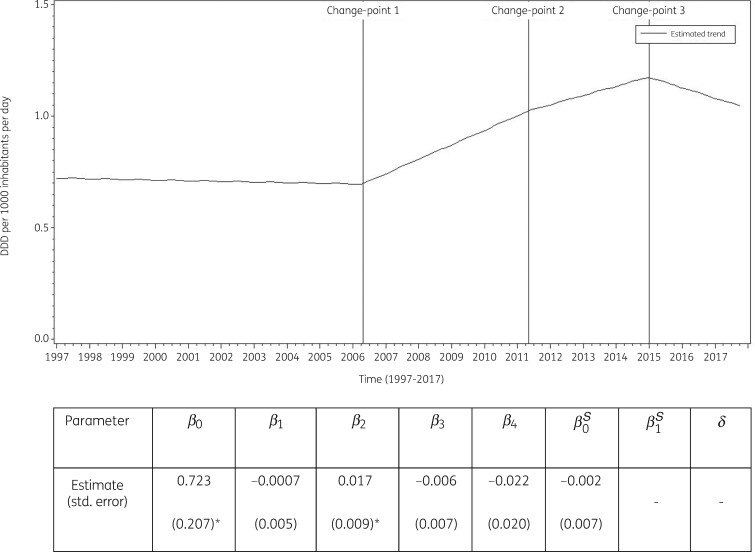
Estimated trend (solid line) of consumption of other antibacterials (ATC J01X) in the community based on quarterly data from 25 EU/EEA countries, 1997–2017. *β_0_*, predicted consumption in the first quarter of 1997; *β_1_*, predicted increase (if positive)/decrease (if negative) in consumption per quarter; *β_2_*, predicted difference in slope after versus before the first change-point; *β_3_*, predicted difference in slope after versus before the second change-point; *β_4_*, predicted difference in slope after versus before the third change-point; *β_0_^S^*, predicted amplitude of the upward winter and downward summer peak in consumption; *β_1_^S^*, predicted increase (if positive)/decrease (if negative) of the amplitude of the upward winter and downward summer peak in consumption per quarter; *δ*, shift in timing of the upward winter and downward summer peak from one year to another. An asterisk indicates that the result was statistically significant at significance level 0.05.

The best fit was obtained for a model including three change-points: one in the second quarter of 2006, another in the second quarter of 2011 and a third one in the first quarter of 2015. The final model fits the observed data well (Figure S8). The longitudinal data analysis estimated an average consumption of other antibacterials in the EU/EEA of 0.723 (SE 0.207) DDD per 1000 inhabitants per day in the first quarter of 1997 which did not change significantly over time: −0.001 (SE 0.005) DDD per 1000 inhabitants per day per quarter up to the second quarter of 2006, then +0.016 (SE 0.010) DDD per 1000 inhabitants per day per quarter up to the second quarter of 2011, then +0.010 (SE 0.013) DDD per 1000 inhabitants per day per quarter up to the first quarter of 2015, and finally −0.011 (SE 0.007) DDD per 1000 inhabitants per day per quarter. Furthermore, the longitudinal data analysis showed no significant seasonal variation for the consumption of other antibacterials (Figure 9).

Based on the fitted model, consumption of other antibacterials in the community in 1997 was significantly higher than average in Belgium, Estonia, Finland, Latvia, Luxembourg and Sweden, and lower than average in Austria, Croatia, Germany, Hungary, Poland, Slovakia, Slovenia, Spain and the United Kingdom (observed profiles shown in Figures S9 and S10). The seasonal variation was significantly larger than average in Czechia. The decrease in consumption of other antibacterials in the community between 1997 and the second quarter of 2006 was significantly larger than average in Czechia, Estonia, Iceland, Latvia and Luxembourg. The increase between the third quarter of 2006 and the second quarter of 2011 was significantly larger than average in Iceland, Lithuania and Portugal. The increase between the third quarter of 2011 and the first quarter of 2015 was significantly larger than average in Lithuania. The decrease between the second quarter of 2015 and the last quarter of 2017 was significantly larger than average in Czechia.

#### Compositional data analysis, 1997–2009

The proportional consumption of polymyxins (J01XB) significantly increased over time relative to that of the other subgroups (Table 3). The proportional consumption of steroid antibacterials (J01XC) significantly decreased relative to that of the other subgroups except imidazole derivatives (J01XD; no significant change). The relative consumption of glycopeptide antibacterials (J01XA) significantly decreased relative to that of other antibacterials (J01XX) and the proportional consumption of imidazole derivatives (J01XD) significantly decreased relative to that of nitrofuran derivatives (J01XE) (Table 6).

**Table 6.** Change in the composition of the consumption of other antibacterial (ATC J01X) in the community, expressed in DDD (ATC/DDD index 2019) per 1000 inhabitants per day, 30 EU/EEA countries, as a function of time during 1997–2017

	J01XA	J01XB	J01XC	J01XD	J01XE	J01XX
J01XA		**−0.0771**	**0.0444**	0.0187	−0.0160	**−0.0287**
J01XB	**0.0771**		**0.1214**	**0.0958**	**0.0611**	**0.0484**
J01XC	**−0.0444**	**−0.1214**	**−0.0611**	−0.0257	**−0.0603**	**−0.0703**
J01XD	−0.0187	**−0.0958**	0.0257		**−0.0347**	0.0257
J01XE	0.0160	**−0.0611**	**0.0603**	**0.0347**		−0.0127
J01XX	**0.0287**	**−0.0484**	**0.0703**	−0.0257	0.0127	

Values are estimated changes in the log ratio of the row versus column subgroup of antibiotics with increasing time. Bold type indicates a statistically significant effect; positive values represent an increase and negative values represent a decrease.

J01XA, glycopeptide antibacterials; J01XB, polymyxins; J01XC, steroid antibacterials; J01XD, imidazole derivatives; J01XE, nitrofuran derivatives; J01XX, other antibacterials.

Trends of proportional consumption for individual countries are shown in Figure S11. When comparing the composition of the consumption of other antibacterials in 2017 with that in 2009, the proportion of polymyxins increased for most of the participating countries. Increases >5% were reported for Ireland [+14.44%; consumption of nitrofurantoin (J01XE01) not included], Slovakia (+11.61%; 2016 data) and Bulgaria (+5.54%). The proportional consumption of steroid antibacterials decreased for most of the countries, with decreases >5% reported for Iceland (−40.96%), Ireland [−20.20%; consumption of nitrofurantoin (J01XE01) not included], Austria (−10.13%), Greece (−7.83%), and France (−7.73%). The proportional consumption of nitrofuran derivatives decreased for most countries, with the largest decreases reported for Hungary (−71.06%), Luxembourg (−68.03%) and Denmark (−46.71%). The proportional consumption of the ‘other antibacterials’ subgroup (J01XX) increased in most countries, with the largest increases reported for Bulgaria (+77.74%), Luxembourg (+68.27%), Hungary (+66.93%) and Denmark (+49.12%). Overall, the increase was mainly attributable to an increased consumption of fosfomycin (J01XX01). The proportions of glycopeptide antibacterials and imidazole derivatives showed both increases and decreases. The largest increases in the consumption of glycopeptide antibacterials in the community were observed for Romania (+14.70%; total care data, i.e. community and hospital sector combined; coverage in 2009 limited to 30%–40%), Ireland [+6.28%; consumption of nitrofurantoin (J01XE01) not included] and Cyprus (+1.90%; total care data), and the largest decreases for Bulgaria (−5.72%), Slovakia (−0.87%; 2016 data) and Greece (−0.67%). The largest increases in the consumption of imidazole derivatives in the community were observed for Slovakia (+22.46%), Cyprus (+6.53%; total care data) and Greece (+2.60%), and the largest decreases for Bulgaria (−77.56%), Romania (−73.75%; total care data; coverage in 2009 limited to 30%–40%) and Lithuania (−52.34%).

## Discussion

This study updates a previous description of the consumption of tetracyclines, sulphonamides and trimethoprim, and other antibacterials in the community in EU/EEA countries.^2^ Most substances reported in this article are prescribed to treat a wide range of infections and acne as well as infections with atypical pathogens such as *Chlamydophila pneumoniae, Mycoplasma pneumoniae* or *Legionella pneumophila*.^8^ While tetracycline consumption did not change significantly over time, its seasonal variation significantly decreased. Four substances represented 99% of consumption of tetracyclines in the community.

In 2017, the proportional consumption of tetracyclines out of all antibacterials for systemic use ranged from 2.51% in Italy to 28.22% in the United Kingdom.^3^ In European countries that are not part of the ESAC-Net but covered by the WHO Europe Antimicrobial Medicines Consumption (AMC) Network, there was also considerable variation, i.e. from <0.1% in North Macedonia to 15.3% in Azerbaijan.^9^

While the consumption of doxycycline (usually prescribed for respiratory infections), minocycline and tetracycline (usually prescribed for long-term treatment of acne) decreased since 2009, the consumption of lymecycline (usually prescribed to treat acne and respiratory infections) increased since 2009, mainly in some northern EU/EEA countries such as Finland, Norway, Sweden and the United Kingdom.

Consumption of sulphonamides and trimethoprim [usually prescribed to treat urinary tract infections (UTIs)] and the composition of this consumption varied considerably between countries. On average, within the EU/EAA, consumption of sulphonamides and trimethoprim significantly decreased over time until 2006 and remained stable thereafter, but seasonal variation continued to significantly decrease over time. Interestingly, considering the change in the composition of consumption of sulphonamides and trimethoprim, the proportion of trimethoprim and derivatives (J01EA) significantly increased over time compared with that of the most commonly prescribed subgroup combinations of sulphonamides and trimethoprim, including derivatives (J01EE), as well as other less-consumed sulphonamide subgroups. Given that total consumption of sulphonamides and trimethoprim did not change significantly after 2006, this implies that consumption of one subgroup was merely replaced by consumption of another subgroup, rather than consumption being reduced overall.

In the EU/EEA, the average consumption as well as the seasonal variation of the consumption of other antibacterials (J01X) did not change significantly over time. Nevertheless, there were large variations between countries with other antibacterials representing from 0.17% in Ireland to 27.46% in Norway of the consumption of antibacterials for systemic use in the community in 2017.^3^ This might be explained by a preference for treating some UTIs with methenamine, which does not drive antimicrobial resistance.^10^ In European countries that are not part of the ESAC-Net but covered by the WHO Europe Antimicrobial Medicines Consumption (AMC) Network considerable variation was also observed, from 1.4% in Albania to 10.8% in Georgia.^9^

In addition, there were large variations between countries and over time for specific subgroups. Consumption of parenteral glycopeptide antibacterials (vancomycin and teicoplanin); polymyxins (colistin) and imidazole derivatives for systemic use (metronidazole and tinidazole) was low in the community in most EU/EEA countries. Consumption of fusidic acid for systemic use, available in parenteral and oral formulation, was also low. Countries where these parenteral substances are prescribed in the community tend to report a high consumption of other antibacterials expressed in packages per 1000 inhabitants per day because each package might represent only one DDD of parenteral antibiotic. As such, Greece ranked high for its consumption of other antibacterials expressed in packages per 1000 inhabitants per day because of its high consumption of parenteral metronidazole, colistin, vancomycin and teicoplanin, which could be related to outpatient parenteral antibiotic therapy (OPAT) to treat pneumonia, UTIs and gastro-intestinal tract infections in Greece.^11^ Italy is also known for its consumption of parenteral antibiotics in the community, often administered at hospital infusion centres.^12^ Teicoplanin and ceftriaxone were the most commonly administered parenteral antibiotics at these centres.^13^ Esposito *et al.*^14^ predicted that the demand for OPAT is likely to further increase in Italy due to the potential saving in hospital care costs and improvements in the allocation of limited healthcare resources, with an increased consumption of teicoplanin until 2010.^15^ We confirmed that indeed teicoplanin for OPAT increased up to 0.06 DDD per 1000 inhabitants per day in Italy until 2010, but decreased steadily thereafter to 0.03 DDD per 1000 inhabitants per day in 2017.

For France and Italy, the higher ranking for their consumption of other antibacterials expressed in packages per 1000 inhabitants per day was also related to the consumption of fosfomycin, which is dispensed as one dose per package and is commonly prescribed for the treatment of UTIs in these countries. Some guidelines may recommend oral fosfomycin as first-line therapy for the treatment of uncomplicated cystitis.^16^ In many countries, resistance to fosfomycin remains low in uropathogenic *Escherichia coli*, while increasing resistance to trimethoprim/sulfamethoxazole, which is still widely used as the first-line agent for the treatment of uncomplicated UTIs, has been reported.^17–20^ Belgium scored high for its consumption of other antibacterials expressed in DDD per 1000 inhabitants per day and higher when expressed in packages per 1000 inhabitants per day, which is related to the high consumption of nitrofuran derivatives (mainly nitrofurantoin) prescribed to treat uncomplicated cystitis as well as for the long-term prophylaxis of UTIs.^21^^,^^22^ The absence of consumption of nitrofuran derivatives reported for Bulgaria, Hungary and Slovakia (2016 data) might be explained by the fact that nitrofurantoin had to be imported (due to manufacturing problems) and therefore was not included in the sales data reported by these countries.

Finally, six substances for parenteral use, i.e. tigecycline (J01AA12), colistin (J01XB01), fosfomycin (J01XX01), linezolid (J01XX08), daptomycin (J01XX09) and tedizolid (J01XX11), for which consumption was reported in 2017, are listed as Reserve antibiotics in the 2019 WHO Access, Watch or Reserve (AWaRe) classification list. Six countries, among which two provided total care data, i.e. community and hospital sector combined, reported consumption of tigecycline. There also were large variations in the consumption of colistin among the 22 countries reporting consumption of this antibiotic in the community in 2017. The AWaRe classification can be used in supporting antibiotic monitoring and optimal prescribing. Individual countries should follow up consumption of Reserve antibiotics, which are considered as ‘last resort’ options for the treatment of confirmed or suspected infections due to multidrug-resistant organisms. These should be prioritized as key targets in national stewardship programmes.^23^

For a more detailed discussion on the limitations of the collected data, we refer to the article on antibacterials for systemic use, included in this series.^4^ For a discussion on the limitations of the statistical approach used in this study and potential explanations for the common change-points detected through these analyses, we refer to the tutorial included in this series.^6^

In conclusion, as for all other main antibiotic groups, there were large variations between EU/EEA countries in the consumption and composition of the consumption for the tetracyclines, sulphonamides and trimethoprim, and other antibacterials in the community. This represents an opportunity to improve the quality of prescription of these antibiotics in many countries.

## Supplementary Material

dkab177_Supplementary_Data

## Supplementary data

Tables S1 to S6 and Figures S1 to S11 are available as Supplementary data at *JAC* Online.
