# Supplementary material for: Consumption of tetracyclines, sulphonamides and trimethoprim, and other antibacterials in the community, European Union/European Economic Area, 1997–2017
Source: J Antimicrob Chemother. 2021 Aug 1;76(Suppl 2):ii45–59. doi: 10.1093/jac/dkab177 (PMC8314111; doi:10.1093/jac/dkab177)
Supplement: dkab177_Supplementary_Data [file dkab177_supplementary_data.docx]

**Supplementary data**

**Table S1. Consumption of tetracyclines (ATC J01A) in the community, expressed in DDD (ATC/DDD index 2019) per 1000 inhabitants per day, 30 EU/EEA countries, 1997-2017.**

| **Country** | **1997** | **1998** | **1999** | **2000** | **2001** | **2002** | **2003** | **2004** | **2005** | **2006** | **2007** | **2008** | **2009** | **2010** | **2011** | **2012** | **2013** | **2014** | **2015** | **2016** | **2017** |
| --- | --- | --- | --- | --- | --- | --- | --- | --- | --- | --- | --- | --- | --- | --- | --- | --- | --- | --- | --- | --- | --- |
| Austria | - | 1.35 | 1.40 | 1.28 | 1.12 | 1.04 | 1.08 | 0.98 | 1.47 | 1.26 | 1.27 | 1.33 | 1.27 | 1.20 | 1.16 | 1.08 | 1.27 | 1.06 | 0.99 | 0.86 | 0.63 |
| Belgium | 4.41 | 4.17 | 3.82 | 3.48 | 2.94 | 2.65 | 2.29 | 2.06 | 1.96 | 1.85 | 1.96 | 2.20 | 2.14 | 2.10 | 2.09 | 2.11 | 2.16 | 2.10 | 2.03 | 1.99 | 1.92 |
| Bulgaria | *-* | *-* | *4.24* | *3.26* | *3.64* | *2.80* | *3.07* | *2.84* | *2.61* | 2.51 | 2.35 | 2.17 | 1.62 | 1.71 | 1.82 | 1.77 | 1.76 | 1.79 | 1.70 | 1.68 | 1.63 |
| Croatia | - | - | - | - | 1.46 | 1.86 | 1.95 | 1.95 | 2.06 | 1.80 | 1.87 | 1.83 | 1.66 | 1.15 | 1.39 | 1.19 | 1.19 | 1.12 | 1.14 | 1.02 | 1.00 |
| Cyprus | - | - | - | - | - | - | - | - | - | *3.11* | *2.51* | *2.82* | *2.87* | *3.27* | *2.76* | *3.09* | *2.79* | *3.30* | *3.61* | *3.84* | *3.50* |
| Czechia | - | 2.89 | 3.13 | - | - | - | 2.86 | 2.69 | 2.91 | 2.81 | 2.70 | 2.52 | 2.39 | 2.27 | 2.18 | 2.03 | 2.31 | 2.00 | 1.98 | - | - |
| Denmark | 0.98 | 0.98 | 0.92 | 0.99 | 0.99 | 1.04 | 1.07 | 1.17 | 1.27 | 1.37 | 1.47 | 1.56 | 1.62 | 1.70 | 1.73 | 1.76 | 1.96 | 1.66 | 1.61 | 1.61 | 1.42 |
| Estonia | - | - | - | - | *2.89* | 2.65 | 2.61 | 2.42 | 2.42 | 2.28 | 2.31 | 2.18 | 2.07 | 1.90 | 2.11 | 1.80 | 1.61 | 1.46 | 1.44 | 1.56 | 1.28 |
| Finland | 4.90 | 4.60 | 4.53 | 4.62 | 4.54 | 3.90 | 3.88 | 3.58 | 3.95 | 3.76 | 3.97 | 4.00 | 4.01 | 4.09 | 4.72 | 4.68 | 4.32 | 4.12 | 3.94 | 3.73 | 3.29 |
| France | 2.99 | 2.96 | 3.11 | 3.29 | 3.10 | 3.43 | 3.33 | 3.46 | 3.40 | 3.33 | 3.34 | 3.44 | 3.39 | 3.16 | 3.07 | 3.30 | 3.35 | 3.21 | 3.26 | 3.24 | 3.00 |
| Germany | 3.09 | 2.89 | 3.07 | 2.97 | 2.75 | 2.68 | 3.48 | 3.18 | 3.45 | 3.23 | 3.32 | 3.22 | 3.09 | 2.74 | 2.61 | 2.34 | 2.42 | 2.11 | 1.97 | 1.90 | 1.76 |
| Greece | 2.73 | 2.70 | 2.84 | 2.80 | 2.68 | 2.75 | 2.55 | *2.65* | *2.56* | *2.52* | *2.46* | *2.42* | 2.00 | *2.35* | 2.44 | 1.91 | 1.83 | 2.37 | 2.58 | 2.69 | 2.79 |
| Hungary | - | 2.63 | 3.11 | 2.29 | 2.19 | 1.93 | 1.94 | 1.77 | 1.74 | 1.44 | 1.40 | 1.40 | 1.35 | 1.38 | 1.28 | 1.18 | 1.18 | 1.18 | 1.21 | 1.11 | 1.07 |
| Iceland | *5.36* | *5.45* | *5.17* | *4.75* | *4.63* | *4.81* | *4.76* | *5.18* | *5.46* | 5.13 | 5.08 | 5.31 | 5.09 | *5.05* | *4.89* | *4.78* | *4.67* | 4.38 | 4.60 | 4.58 | 4.96 |
| Ireland | - | 3.16 | 3.15 | 3.02 | 3.23 | 3.44 | 3.64 | 3.83 | 3.26 | 3.34 | 3.32 | 3.19 | 2.74 | 2.60 | 2.78 | 2.93 | 2.96 | 2.72 | 2.57 | 2.54 | 2.80 |
| Italy | - | - | 0.53 | 0.52 | 0.53 | 0.33 | 0.49 | 0.48 | 0.47 | 0.49 | 0.50 | 0.54 | 0.52 | 0.54 | 0.51 | 0.54 | 0.56 | 0.58 | 0.55 | 0.56 | 0.48 |
| Latvia | - | - | - | - | - | 2.06 | - | 2.46 | 2.64 | 2.57 | 2.68 | 2.37 | 2.18 | 2.38 | 2.53 | 2.52 | 2.35 | 2.19 | 2.18 | 2.18 | 2.17 |
| Lithuania | - | - | - | - | - | - | - | - | - | *1.72* | *2.47* | *2.37* | *2.00* | *1.68* | *1.65* | 1.53 | 1.54 | 1.37 | 1.33 | 1.45 | 1.40 |
| Luxembourg | 4.04 | 3.47 | 3.28 | 2.92 | 2.75 | 2.61 | 2.40 | 2.28 | 2.21 | 1.99 | 2.09 | 2.16 | 2.08 | 1.96 | 1.99 | 1.88 | 1.85 | 1.73 | 1.69 | 1.78 | 1.09 |
| Malta | - | - | - | - | - | - | - | - | - | - | 0.93 | 1.11 | 1.10 | 1.02 | 1.06 | 1.68 | 1.00 | 1.20 | 0.75 | 1.77 | 1.60 |
| Netherlands | 2.64 | 2.55 | 2.49 | 2.47 | 2.39 | 2.33 | 2.22 | 2.22 | 2.41 | 2.39 | 2.55 | 2.64 | 2.68 | 2.66 | 2.60 | 2.49 | 2.33 | 2.23 | 2.25 | 2.10 | 1.98 |
| Norway | - | 3.24 | - | - | 3.05 | 3.05 | 2.95 | 2.89 | 3.04 | 2.76 | 2.86 | 2.80 | 2.71 | 2.77 | 3.09 | 3.45 | 3.24 | 3.12 | 3.08 | 2.89 | 2.73 |
| Poland | - | 3.90 | 3.90 | 4.07 | 4.15 | 3.90 | - | 2.85 | 2.91 | - | 2.77 | 2.50 | 2.47 | 2.11 | 2.07 | 2.39 | 2.51 | 2.40 | 2.39 | 2.34 | 2.39 |
| Portugal | 1.97 | 1.84 | 1.76 | 1.57 | 1.37 | 1.24 | 1.22 | 1.07 | 1.01 | 0.90 | - | 0.82 | 0.72 | 0.71 | 1.02 | 1.08 | 0.75 | 0.83 | 0.83 | 0.81 | 0.83 |
| Romania^a^ | - | - | - | - | - | - | - | - | - | - | - | - | *0.11* | - | *1.34* | *1.33* | *1.19* | *1.13* | *1.10* | *0.93* | *0.85* |
| Slovakia | - | - | 1.92 | 2.08 | 2.23 | 2.37 | 2.53 | 2.36 | 2.29 | 1.92 | 1.52 | 1.51 | 1.50 | - | *1.45* | 1.37 | 1.58 | 1.67 | 1.73 | 1.67 | - |
| Slovenia | 0.93 | 0.86 | 0.85 | 0.81 | 0.77 | 0.70 | 0.71 | 0.61 | 0.57 | 0.55 | 0.58 | 0.52 | <0.01 | 0.04 | 0.32 | 0.43 | 0.46 | 0.45 | 0.42 | 0.41 | 0.30 |
| Spain^b^ | 0.82 | 0.75 | 0.67 | 0.61 | 0.62 | 0.62 | 0.62 | 0.62 | 0.59 | 0.59 | 0.59 | 0.60 | 0.60 | 0.70 | 0.70 | 0.68 | 0.74 | 0.79 | 0.74 | 1.48 | 1.47 |
| Sweden | 2.97 | 3.35 | 3.33 | 3.28 | 3.27 | 3.10 | 3.03 | 3.06 | 3.25 | 3.32 | 3.37 | 3.22 | 3.03 | 3.33 | 3.54 | 3.44 | 2.85 | 2.66 | 2.54 | 2.40 | 2.35 |
| UK | 3.45 | 3.41 | 3.20 | 3.16 | 3.22 | 3.29 | 3.30 | 3.35 | 3.34 | 3.34 | 3.51 | 3.74 | 3.96 | 4.15 | 4.32 | 4.64 | 4.90 | 5.03 | 4.97 | 4.86 | 4.81 |

**-**, no consumption reported; Numbers reported in *italic* are total care data, i.e. community and hospital sector combined; ^a^Data for Romania have a coverage in 2009 limited to 30-40%; ^b^Data for Spain include private prescriptions from 2016 onwards.

**Table S2. Consumption of tetracyclines (ATC J01A) in the community, expressed in packages per 1000 inhabitants per day, 23 EU/EEA countries, 2006-2017.**

| **Country** | **2006** | **2007** | **2008** | **2009** | **2010** | **2011** | **2012** | **2013** | **2014** | **2015** | **2016** | **2017** |
| --- | --- | --- | --- | --- | --- | --- | --- | --- | --- | --- | --- | --- |
| Austria | - | 0.10 | 0.10 | 0.09 | 0.09 | 0.08 | 0.08 | 0.09 | 0.08 | 0.07 | 0.06 | 0.04 |
| Belgium^a^ | - | 0.14 | 0.15 | 0.14 | 0.14 | 0.13 | 0.13 | 0.13 | 0.13 | 0.13 | 0.14 | 0.13 |
| Bulgaria | 0.36 | 0.33 | 0.30 | 0.26 | 0.24 | 0.25 | 0.24 | 0.24 | 0.23 | 0.22 | 0.20 | 0.18 |
| Croatia | - | 0.22 | 0.20 | 0.17 | 0.07 | 0.13 | 0.10 | 0.10 | 0.11 | 0.11 | 0.08 | 0.08 |
| Czechia | - | 0.23 | - | - | 0.18 | 0.17 | 0.16 | 0.18 | 0.14 | 0.15 | - | - |
| Denmark | - | 0.07 | 0.07 | 0.07 | 0.07 | 0.08 | 0.08 | 0.08 | 0.07 | 0.06 | 0.05 | 0.05 |
| Estonia | 0.23 | 0.24 | 0.22 | 0.21 | 0.20 | 0.22 | 0.18 | 0.17 | 0.15 | 0.14 | 0.15 | 0.13 |
| Finland | - | - | 0.22 | 0.22 | 0.22 | 0.27 | 0.26 | 0.23 | 0.22 | 0.21 | 0.20 | 0.17 |
| France | - | - | - | - | 0.18 | 0.18 | 0.16 | 0.16 | 0.16 | 0.15 | 0.14 | 0.13 |
| Greece | *0.31* | *0.28* | *0.28* | 0.24 | *0.28* | 0.28 | 0.24 | 0.25 | 0.30 | 0.33 | 0.34 | 0.35 |
| Iceland | - | - | - | - | *0.27* | *0.27* | *0.38* | *0.23* | 0.22 | 0.23 | 0.22 | 0.24 |
| Ireland | - | 0.24 | - | - | 0.22 | 0.23 | 0.25 | 0.25 | 0.24 | 0.23 | 0.24 | 0.26 |
| Italy | - | - | 0.07 | - | 0.07 | 0.06 | 0.07 | 0.07 | 0.07 | 0.06 | 0.07 | 0.05 |
| Latvia | - | - | - | - | 0.22 | 0.23 | 0.23 | 0.22 | 0.20 | 0.20 | 0.20 | 0.20 |
| Lithuania | - | *0.26* | *0.25* | *0.21* | *0.17* | *0.17* | 0.16 | 0.16 | 0.14 | 0.13 | 0.14 | 0.14 |
| Luxembourg | 0.15 | 0.15 | 0.15 | 0.14 | 0.13 | 0.14 | 0.13 | 0.12 | 0.12 | 0.12 | 0.11 | - |
| Netherlands^b^ | - | - | 0.31 | 0.32 | - | - | - | - | - | - | - | - |
| Portugal | - | - | 0.06 | 0.05 | 0.05 | 0.07 | 0.07 | 0.05 | 0.05 | 0.05 | 0.05 | 0.05 |
| Slovakia | - | - | - | - | - | *0.11* | 0.10 | 0.11 | 0.09 | 0.08 | 0.08 | - |
| Slovenia | - | 0.03 | 0.02 | <0.01 | <0.01 | 0.02 | 0.02 | 0.02 | 0.02 | 0.02 | 0.02 | 0.03 |
| Spain^c^ | **-** | **-** | **-** | - | 0.04 | 0.04 | 0.04 | 0.04 | 0.04 | 0.03 | 0.06 | 0.06 |
| Sweden | **-** | **-** | **-** | 0.15 | 0.15 | 0.16 | 0.15 | 0.13 | 0.12 | 0.11 | 0.10 | 0.10 |
| UK | **-** | **-** | **-** | - | - | - | - | - | - | 0.01 | - | - |

**-**, no consumption reported; Numbers reported in *italic* are total care data, i.e. community and hospital sector combined; ^a^Data for Belgium are slightly overestimated
from 2016 onwards (nursing homes counting units versus packages before 2016); ^b^Data for the Netherlands are based on average package size; ^c^Data for Spain include private prescriptions from 2016 onwards.

**Table S3. Consumption of sulfonamides and trimethoprim (ATC J01E) in the community, expressed in DDD (ATC/DDD index 2019) per 1000 inhabitants per day, 30 EU/EEA countries, 1997-2017.**

| **Country** | **1997** | **1998** | **1999** | **2000** | **2001** | **2002** | **2003** | **2004** | **2005** | **2006** | **2007** | **2008** | **2009** | **2010** | **2011** | **2012** | **2013** | **2014** | **2015** | **2016** | **2017** |
| --- | --- | --- | --- | --- | --- | --- | --- | --- | --- | --- | --- | --- | --- | --- | --- | --- | --- | --- | --- | --- | --- |
| **Austria** | - | **0.69** | **0.68** | **0.66** | **0.50** | **0.48** | **0.40** | **0.32** | **0.32** | **0.31** | **0.31** | **0.29** | **0.29** | **0.26** | **0.24** | **0.22** | **0.23** | **0.20** | **0.19** | **0.20** | **0.22** |
| EA | - | 0.32 | 0.34 | 0.33 | 0.30 | 0.31 | 0.23 | 0.20 | 0.21 | 0.20 | 0.20 | 0.19 | 0.18 | 0.17 | 0.15 | 0.14 | 0.15 | 0.13 | 0.12 | 0.11 | 0.11 |
| EB | - | - | - | - | - | - | - | - | - | - | - | - | - | - | - | - | - | - | - | - | - |
| EC | - | - | - | - | - | - | <0.01 | <0.01 | - | - | - | - | - | - | - | - | - | - | - | - | - |
| ED | - | - | - | - | - | - | - | - | - | - | - | - | - | - | - | - | - | - | - | - | - |
| EE | - | 0.37 | 0.34 | 0.33 | 0.20 | 0.18 | 0.17 | 0.11 | 0.11 | 0.11 | 0.11 | 0.10 | 0.11 | 0.09 | 0.09 | 0.08 | 0.08 | 0.07 | 0.08 | 0.09 | 0.11 |
| **Belgium** | **0.71** | **0.67** | **0.63** | **0.56** | **0.49** | **0.44** | **0.39** | **0.36** | **0.35** | **0.35** | **0.37** | **0.38** | - | **0.26** | **0.30** | - | **0.27** | **0.18** | **0.21** | **0.20** | **0.19** |
| EA | 0.01 | 0.01 | 0.01 | 0.01 | 0.01 | <0.01 | <0.01 | - | - | - | - | - | - | - | - | - | - | - | - | - | - |
| EB | - | - | - | - | - | - | - | - | - | - | - | - | - | - | - | - | - | - | - | - | - |
| EC | - | - | - | - | - | - | - | - | - | - | - | - | - | - | - | - | - | - | - | - | - |
| ED | 0.06 | 0.05 | 0.04 | 0.04 | 0.04 | 0.03 | <0.01 | - | - | - | - | - | - | - | - | - | - | - | - | - | - |
| EE | 0.64 | 0.60 | 0.57 | 0.51 | 0.44 | 0.41 | 0.39 | 0.36 | 0.35 | 0.35 | 0.37 | 0.38 | - | 0.26 | 0.30 | - | 0.27 | 0.18 | 0.21 | 0.20 | 0.19 |
| **Bulgaria** | - | - | - | ***1.13*** | ***1.31*** | ***0.94*** | ***1.08*** | ***0.97*** | ***0.88*** | **0.98** | **0.99** | **0.99** | **0.86** | **0.87** | **0.90** | **0.82** | **0.83** | **0.84** | **0.79** | **0.78** | **0.85** |
| EA | - | - | - | *-* | *-* | *-* | *-* | *-* | *-* | - | - | - | - | - | - | - | - | - | - | - | - |
| EB | - | - | - | *-* | *-* | *-* | *-* | *-* | *-* | - | - | - | - | - | - | - | - | - | - | - | - |
| EC | - | - | - | *-* | *-* | *-* | *-* | *-* | *-* | - | - | - | - | - | - | - | - | - | - | - | - |
| ED | - | - | - | *0.21* | *0.10* | *-* | *-* | *-* | *-* | - | - | - | - | - | - | - | - | - | - | - | - |
| EE | - | - | - | *0.93* | *1.20* | *0.94* | *1.08* | *0.97* | *0.88* | 0.98 | 0.99 | 0.99 | 0.86 | 0.87 | 0.90 | 0.82 | 0.83 | 0.84 | 0.79 | 0.78 | 0.85 |
| **Croatia** | - | - | - | - | **1.74** | **1.90** | **1.76** | **1.67** | **1.61** | **1.40** | **1.44** | **1.24** | **1.03** | **0.87** | **0.73** | **0.67** | **0.67** | **0.65** | **0.63** | **0.59** | **0.55** |
| EA | - | - | - | - | - | - | - | - | - | - | - | - | - | - | - | - | - | - | - | - | - |
| EB | - | - | - | - | - | - | - | - | - | - | - | - | - | - | - | - | - | - | - | - | - |
| EC | - | - | - | - | - | - | - | - | - | - | - | - | - | - | - | - | - | - | - | - | - |
| ED | - | - | - | - | - | - | - | - | - | - | - | - | - | - | - | - | - | - | - | - | - |
| EE | - | - | - | - | 1.74 | 1.90 | 1.76 | 1.67 | 1.61 | 1.40 | 1.44 | 1.24 | 1.03 | 0.87 | 0.73 | 0.67 | 0.67 | 0.65 | 0.63 | 0.59 | 0.55 |
| **Cyprus** | - | - | - | - | - | - | - | - | - | ***0.44*** | ***0.35*** | ***0.42*** | ***0.46*** | ***0.37*** | ***0.30*** | ***0.35*** | ***0.31*** | ***0.28*** | ***0.22*** | ***0.01*** | ***0.29*** |
| EA | - | - | - | - | - | - | - | - | - | *0.01* | *0.01* | *0.01* | *0.01* | *0.01* | *0.01* | *0.01* | *<0.01* | *<0.01* | *<0.01* | *<0.01* | *<0.01* |
| EB | - | - | - | - | - | - | - | - | - | *-* | *-* | *-* | *-* | *-* | *-* | *-* | *-* | *-* | *-* | *-* | *-* |
| EC | - | - | - | - | - | - | - | - | - | *<0.01* | *<0.01* | *<0.01* | *<0.01* | *<0.01* | *0.01* | *<0.01* | *<0.01* | *<0.01* | *<0.01* | *<0.01* | *0.01* |
| ED | - | - | - | - | - | - | - | - | - | *-* | *-* | *-* | *-* | *-* | *-* | *-* | *-* | *-* | *-* | *-* | *-* |
| EE | - | - | - | - | - | - | - | - | - | *0.42* | *0.34* | *0.41* | *0.44* | *0.36* | *0.28* | *0.34* | *0.31* | *0.28* | *0.21* | *-* | *0.28* |
| Country, community consumption of sulfonamides and trimethoprim (J01E); EA, consumption of trimethoprim and derivatives (J01EA); EB, consumption of short-acting sulfonamides (J01EB); EC, consumption of intermediate-acting sulfonamides (J01EC); ED, consumption of long-acting sulfonamides (J01ED); EE, consumption of combinations of sulfonamides and trimethoprim (J01EE); **-**, no consumption reported; Numbers reported in *italic* are total care data, i.e. community and hospital sector combined; ^a^Data for Romania have a coverage in 2009 limited to 30-40%; ^b^Data for Spain include private prescriptions from 2016 onwards. | | | | | | | | | | | | | | | | | | | | | |
| **Czechia** | - | **1.69** | **1.62** | - | - | - | **1.18** | **1.18** | **1.24** | **1.02** | **1.10** | **0.87** | **0.89** | **0.85** | **0.81** | **0.74** | **0.81** | **0.78** | **0.88** | - | - |
| EA | - | 0.16 | 0.25 | - | - | - | 0.21 | 0.22 | 0.24 | 0.18 | 0.29 | 0.14 | 0.14 | 0.14 | 0.13 | 0.12 | 0.13 | 0.12 | 0.13 | - | - |
| EB | - | 0.01 | <0.01 | - | - | - | - | - | - | - | - | - | - | - | - | - | - | - | - | - | - |
| EC | - | - | - | - | - | - | - | - | - | - | - | - | - | - | - | - | - | - | - | - | - |
| ED | - | - | - | - | - | - | - | - | - | - | - | - | - | - | - | - | - | - | - | - | - |
| EE | - | 1.53 | 1.36 | - | - | - | 0.97 | 0.95 | 1.00 | 0.84 | 0.81 | 0.73 | 0.75 | 0.71 | 0.69 | 0.62 | 0.68 | 0.65 | 0.76 | - | - |
| **Denmark** | **0.79** | **0.77** | **0.74** | **0.73** | **0.74** | **0.75** | **0.76** | **0.77** | **0.79** | **0.82** | **0.81** | **0.77** | **0.75** | **0.77** | **0.74** | **0.77** | **0.75** | **0.76** | **0.73** | **0.72** | **0.71** |
| EA | 0.30 | 0.32 | 0.32 | 0.33 | 0.35 | 0.36 | 0.38 | 0.41 | 0.44 | 0.47 | 0.49 | 0.49 | 0.48 | 0.51 | 0.50 | 0.53 | 0.53 | 0.55 | 0.56 | 0.56 | 0.56 |
| EB | 0.41 | 0.41 | 0.38 | 0.37 | 0.36 | 0.36 | 0.36 | 0.36 | 0.35 | 0.35 | 0.31 | 0.28 | 0.27 | 0.26 | 0.24 | 0.22 | 0.22 | 0.21 | 0.18 | 0.16 | 0.15 |
| EC | - | - | - | - | - | - | - | - | - | - | - | - | - | - | - | - | - | - | - | - | - |
| ED | - | - | - | - | - | - | - | - | - | - | - | - | - | - | - | - | - | - | - | - | - |
| EE | 0.08 | 0.04 | 0.03 | 0.04 | 0.04 | 0.03 | 0.03 | <0.01 | <0.01 | <0.01 | <0.01 | - | <0.01 | <0.01 | <0.01 | 0.02 | - | - | <0.01 | - | - |
| **Estonia** | - | - | - | - | ***1.39*** | **1.06** | **0.79** | **0.62** | **0.56** | **0.66** | **0.53** | **0.46** | **0.42** | **0.37** | **0.40** | **0.39** | **0.40** | **0.38** | **0.43** | **0.40** | **0.42** |
| EA | - | - | - | - | *0.02* | 0.02 | 0.02 | 0.02 | 0.02 | 0.02 | 0.03 | 0.02 | 0.02 | 0.02 | 0.02 | 0.03 | 0.03 | 0.03 | 0.03 | 0.02 | 0.02 |
| EB | - | - | - | - | *<0.01* | <0.01 | - | - | - | - | - | - | - | - | - | - | - | - | - | - | - |
| EC | - | - | - | - | *-* | <0.01 | <0.01 | <0.01 | - | - | - | - | - | - | - | - | - | - | - | - | - |
| ED | - | - | - | - | *0.15* | 0.08 | 0.06 | 0.04 | 0.02 | <0.01 | - | - | - | - | - | - | - | - | - | - | - |
| EE | - | - | - | - | *1.22* | 0.96 | 0.71 | 0.56 | 0.53 | 0.63 | 0.51 | 0.44 | 0.40 | 0.35 | 0.38 | 0.37 | 0.37 | 0.36 | 0.40 | 0.38 | 0.39 |
| **Finland** | **2.09** | **1.96** | **1.99** | **1.90** | **1.83** | **1.72** | **1.68** | **1.67** | **1.60** | **1.52** | **1.53** | **1.05** | **1.05** | **1.03** | **1.54** | **1.41** | **1.35** | **1.31** | **1.25** | **1.11** | **1.03** |
| EA | 1.21 | 1.18 | 1.26 | 1.26 | 1.22 | 1.20 | 1.18 | 1.23 | 1.18 | 1.13 | 1.12 | 1.05 | 1.05 | 1.03 | 1.06 | 0.99 | 0.94 | 0.89 | 0.85 | 0.79 | 0.73 |
| EB | - | - | - | - | - | - | - | - | - | - | - | - | - | - | - | - | - | - | - | - | - |
| EC | - | - | - | - | - | - | - | - | - | - | - | - | - | - | - | - | - | - | - | - | - |
| ED | - | - | - | - | - | - | - | - | - | - | - | - | - | - | - | - | - | - | - | - | - |
| EE | 0.88 | 0.78 | 0.73 | 0.65 | 0.61 | 0.51 | 0.50 | 0.44 | 0.42 | 0.39 | 0.41 | - | - | - | 0.48 | 0.42 | 0.41 | 0.42 | 0.40 | 0.31 | 0.29 |
| **France** | **0.72** | **0.66** | **0.60** | **0.56** | **0.53** | **0.49** | **0.45** | **0.47** | **0.47** | **0.46** | **0.48** | **0.47** | **0.42** | **0.41** | **0.38** | **0.44** | **0.27** | **0.48** | **0.41** | **0.41** | **0.41** |
| EA | <0.01 | <0.01 | <0.01 | <0.01 | <0.01 | 0.02 | <0.01 | - | - | - | - | - | - | - | - | - | - | - | - | - | <0.01 |
| EB | 0.07 | 0.06 | 0.03 | 0.02 | 0.02 | - | 0.01 | 0.01 | 0.01 | - | - | - | 0.01 | 0.01 | <0.01 | <0.01 | <0.01 | <0.01 | <0.01 | <0.01 | <0.01 |
| EC | 0.05 | 0.04 | 0.04 | 0.04 | 0.04 | 0.04 | 0.05 | 0.04 | 0.04 | 0.04 | 0.03 | 0.04 | 0.02 | 0.03 | - | - | 0.03 | 0.03 | 0.03 | 0.02 | 0.02 |
| ED | - | - | - | - | - | - | - | - | - | - | - | - | - | - | - | - | - | - | - | - | - |
| EE | 0.60 | 0.56 | 0.52 | 0.49 | 0.46 | 0.43 | 0.38 | 0.42 | 0.42 | 0.43 | 0.45 | 0.44 | 0.40 | 0.38 | 0.38 | 0.44 | 0.25 | 0.45 | 0.38 | 0.38 | 0.39 |
| Country, community consumption of sulfonamides and trimethoprim (J01E); EA, consumption of trimethoprim and derivatives (J01EA); EB, consumption of short-acting sulfonamides (J01EB); EC, consumption of intermediate-acting sulfonamides (J01EC); ED, consumption of long-acting sulfonamides (J01ED); EE, consumption of combinations of sulfonamides and trimethoprim (J01EE); **-**, no consumption reported; Numbers reported in *italic* are total care data, i.e. community and hospital sector combined; ^a^Data for Romania have a coverage in 2009 limited to 30-40%; ^b^Data for Spain include private prescriptions from 2016 onwards. | | | | | | | | | | | | | | | | | | | | | |
| **Germany** | **1.24** | **1.12** | **1.16** | **1.13** | **1.09** | **1.02** | **0.96** | **0.93** | **0.94** | **0.89** | **0.86** | **0.81** | **0.73** | **0.69** | **0.61** | **0.55** | **0.53** | **0.50** | **0.47** | **0.46** | **0.46** |
| EA | 0.03 | 0.03 | 0.03 | 0.04 | 0.04 | 0.04 | 0.04 | 0.05 | 0.05 | 0.05 | 0.05 | 0.05 | 0.05 | 0.05 | 0.05 | 0.05 | 0.05 | 0.05 | 0.05 | 0.05 | 0.05 |
| EB | - | - | - | - | - | - | - | - | - | - | - | - | - | - | - | - | - | - | - | - | - |
| EC | 0.02 | 0.02 | 0.02 | 0.01 | 0.01 | 0.01 | 0.01 | 0.01 | 0.01 | 0.01 | 0.01 | 0.01 | 0.01 | 0.01 | 0.01 | 0.01 | 0.01 | 0.01 | 0.01 | 0.01 | 0.01 |
| ED | 0.01 | <0.01 | <0.01 | <0.01 | 0.01 | 0.01 | <0.01 | - | - | - | - | - | - | - | - | - | - | - | - | - | - |
| EE | 1.18 | 1.07 | 1.11 | 1.08 | 1.03 | 0.97 | 0.91 | 0.87 | 0.88 | 0.82 | 0.80 | 0.75 | 0.67 | 0.63 | 0.55 | 0.49 | 0.47 | 0.44 | 0.41 | 0.40 | 0.40 |
| **Greece** | **0.91** | **0.98** | **0.79** | **0.68** | **0.59** | **0.55** | **0.61** | ***0.68*** | ***0.69*** | ***0.48*** | ***0.41*** | ***0.42*** | **0.36** | ***0.30*** | **0.31** | **0.32** | **0.32** | **0.34** | **0.41** | **0.37** | **0.32** |
| EA | - | - | - | - | - | - | - | *-* | *-* | *-* | *-* | *-* | - | *-* | - | - | - | - | - | - | - |
| EB | - | - | - | - | - | - | - | *-* | *-* | *-* | *-* | *-* | - | *-* | - | - | - | - | - | - | - |
| EC | - | - | - | - | - | - | - | *-* | *-* | *-* | *-* | *-* | - | *-* | - | <0.01 | <0.01 | <0.01 | <0.01 | <0.01 | <0.01 |
| ED | - | - | - | - | - | - | - | *-* | *-* | *-* | *-* | *-* | - | *-* | - | - | - | - | - | - | - |
| EE | 0.91 | 0.98 | 0.79 | 0.68 | 0.59 | 0.55 | 0.61 | *0.68* | *0.69* | *0.48* | *0.41* | *0.42* | 0.36 | *0.30* | 0.31 | 0.32 | 0.32 | 0.34 | 0.40 | 0.37 | 0.32 |
| **Hungary** | - | **1.39** | **1.52** | **1.16** | **1.11** | **1.01** | **1.24** | **1.09** | **0.99** | **0.82** | **0.73** | **0.69** | **0.65** | **0.60** | **0.56** | **0.53** | **0.53** | **0.50** | **0.51** | **0.48** | **0.44** |
| EA | - | - | - | - | - | - | - | - | - | - | - | - | - | - | - | - | - | - | - | - | - |
| EB | - | - | - | - | - | - | - | - | - | - | - | - | - | - | - | - | - | - | - | - | - |
| EC | - | - | - | - | - | - | - | - | - | - | - | - | - | - | - | - | - | - | - | - | - |
| ED | - | - | - | - | - | - | - | - | - | - | - | - | - | - | - | - | - | - | - | - | - |
| EE | - | 1.39 | 1.52 | 1.16 | 1.11 | 1.01 | 1.24 | 1.09 | 0.99 | 0.82 | 0.73 | 0.69 | 0.65 | 0.60 | 0.56 | 0.53 | 0.53 | 0.50 | 0.51 | 0.48 | 0.44 |
| **Iceland** | ***2.44*** | ***2.50*** | ***2.16*** | ***2.21*** | ***2.13*** | ***1.97*** | ***1.92*** | ***1.91*** | ***1.97*** | **1.55** | **1.40** | **1.36** | **1.08** | ***0.95*** | ***0.95*** | ***0.97*** | ***0.81*** | **0.72** | **0.58** | **0.20** | **0.22** |
| EA | *0.81* | *0.96* | *0.83* | *0.96* | *0.95* | *0.79* | *0.82* | *0.85* | *0.93* | 0.76 | 0.70 | 0.65 | 0.57 | *0.42* | *0.50* | *0.49* | *0.45* | 0.41 | 0.27 | 0.20 | 0.20 |
| EB | *-* | *-* | *-* | *-* | *-* | *-* | *-* | *-* | *-* | - | - | - | - | *-* | *-* | *-* | *-* | - | - | - | - |
| EC | *-* | *-* | *-* | *-* | *-* | *-* | *-* | *-* | *-* | <0.01 | - | - | - | *-* | *-* | *-* | *-* | - | - | - | 0.01 |
| ED | *-* | *-* | *-* | *-* | *-* | *-* | *-* | *-* | *-* | - | - | - | - | *-* | *-* | *-* | *-* | - | - | - | - |
| EE | *1.63* | *1.54* | *1.33* | *1.25* | *1.18* | *1.17* | *1.11* | *1.05* | *1.04* | 0.79 | 0.70 | 0.71 | 0.51 | *0.53* | *0.45* | *0.49* | *0.36* | 0.30 | 0.31 | - | - |
| **Ireland** | - | **0.92** | **0.92** | **0.90** | **0.91** | **0.93** | **0.68** | **0.82** | **0.90** | **0.90** | **0.70** | **1.00** | **1.13** | **1.14** | **1.18** | **1.16** | **0.98** | **1.00** | **1.04** | **1.10** | **0.88** |
| EA | - | 0.69 | 0.72 | 0.73 | 0.76 | 0.78 | 0.53 | 0.66 | 0.74 | 0.73 | 0.70 | 0.66 | 0.83 | 0.85 | 0.89 | 0.87 | 0.68 | 0.70 | 0.88 | 0.91 | 0.88 |
| EB | - | <0.01 | <0.01 | - | - | - | - | - | - | - | - | - | - | - | - | - | - | - | - | - | - |
| EC | - | - | - | - | - | - | - | - | - | - | - | - | - | - | - | - | - | - | - | - | - |
| ED | - | 0.01 | <0.01 | - | - | - | - | - | - | - | - | - | - | - | - | - | - | - | - | - | - |
| EE | - | 0.22 | 0.20 | 0.17 | 0.16 | 0.15 | 0.16 | 0.16 | 0.16 | 0.17 | - | 0.34 | 0.30 | 0.29 | 0.30 | 0.29 | 0.30 | 0.30 | 0.16 | 0.19 | - |
| Country, community consumption of sulfonamides and trimethoprim (J01E); EA, consumption of trimethoprim and derivatives (J01EA); EB, consumption of short-acting sulfonamides (J01EB); EC, consumption of intermediate-acting sulfonamides (J01EC); ED, consumption of long-acting sulfonamides (J01ED); EE, consumption of combinations of sulfonamides and trimethoprim (J01EE); **-**, no consumption reported; Numbers reported in *italic* are total care data, i.e. community and hospital sector combined; ^a^Data for Romania have a coverage in 2009 limited to 30-40%; ^b^Data for Spain include private prescriptions from 2016 onwards. | | | | | | | | | | | | | | | | | | | | | |
| **Italy** | - | - | **1.08** | **0.87** | **0.81** | **0.73** | **0.70** | **0.62** | **0.59** | **0.56** | **0.53** | **0.50** | **0.47** | **0.46** | **0.36** | **0.36** | **0.35** | **0.34** | **0.34** | **0.37** | **0.66** |
| EA | - | - | - | - | - | - | - | - | - | - | - | - | - | - | - | - | - | - | - | - | - |
| EB | - | - | - | - | - | - | - | - | - | - | - | - | - | - | - | - | - | - | - | - | - |
| EC | - | - | - | - | - | <0.01 | <0.01 | <0.01 | <0.01 | <0.01 | <0.01 | <0.01 | <0.01 | <0.01 | <0.01 | <0.01 | - | - | - | - | - |
| ED | - | - | 0.20 | 0.10 | 0.04 | 0.03 | 0.02 | <0.01 | <0.01 | <0.01 | - | - | - | - | - | - | - | - | - | - | - |
| EE | - | - | 0.88 | 0.77 | 0.77 | 0.70 | 0.67 | 0.62 | 0.59 | 0.56 | 0.53 | 0.50 | 0.47 | 0.45 | 0.36 | 0.36 | 0.35 | 0.34 | 0.34 | 0.37 | 0.66 |
| **Latvia** | - | - | - | - | - | **1.10** | - | **1.05** | **1.05** | **<0.01** | **<0.01** | **0.87** | **1.13** | **0.93** | **1.02** | **0.95** | **0.97** | **0.85** | **0.84** | **0.81** | **0.82** |
| EA | - | - | - | - | - | 0.01 | - | <0.01 | <0.01 | <0.01 | <0.01 | <0.01 | <0.01 | <0.01 | <0.01 | <0.01 | 0.01 | 0.01 | 0.01 | 0.01 | 0.01 |
| EB | - | - | - | - | - | - | - | - | - | - | - | - | - | - | - | - | - | - | - | - | - |
| EC | - | - | - | - | - | - | - | - | - | - | - | - | - | - | - | <0.01 | <0.01 | <0.01 | <0.01 | <0.01 | <0.01 |
| ED | - | - | - | - | - | 0.07 | - | - | - | - | - | - | - | - | - | - | - | - | - | - | - |
| EE | - | - | - | - | - | 1.02 | - | 1.05 | 1.05 | - | - | 0.86 | 1.13 | 0.93 | 1.02 | 0.95 | 0.96 | 0.84 | 0.84 | 0.80 | 0.82 |
| **Lithuania** | - | - | - | - | - | - | - | - | - | ***0.01*** | ***0.01*** | ***0.01*** | ***0.01*** | ***0.38*** | ***0.38*** | **0.37** | **0.39** | **0.36** | **0.36** | **0.01** | **0.01** |
| EA | - | - | - | - | - | - | - | - | - | *0.01* | *0.01* | *0.01* | *0.01* | *0.01* | *0.01* | 0.01 | 0.01 | 0.01 | 0.01 | 0.01 | 0.01 |
| EB | - | - | - | - | - | - | - | - | - | *-* | *-* | *-* | *-* | *-* | *-* | - | - | - | - | - | - |
| EC | - | - | - | - | - | - | - | - | - | *-* | *-* | *-* | *-* | *-* | *-* | - | - | - | - | - | - |
| ED | - | - | - | - | - | - | - | - | - | *-* | *-* | *-* | *-* | *-* | *-* | - | - | - | - | - | - |
| EE | - | - | - | - | - | - | - | - | - | *-* | *-* | *-* | *-* | *0.37* | *0.38* | 0.36 | 0.38 | 0.35 | 0.35 | - | - |
| **Luxembourg** | **0.76** | **0.66** | **0.64** | **0.52** | **0.49** | **0.46** | **0.42** | **0.37** | **0.36** | **0.34** | **0.34** | **0.34** | **0.37** | **0.35** | **0.33** | **0.32** | **0.32** | **0.31** | **0.28** | **0.27** | **0.57** |
| EA | <0.01 | <0.01 | <0.01 | <0.01 | <0.01 | <0.01 | - | - | - | - | - | - | - | - | - | - | - | - | - | - | - |
| EB | - | - | - | - | - | - | - | - | - | - | - | - | - | - | - | - | - | - | - | - | - |
| EC | - | - | - | - | - | - | - | - | - | - | - | - | - | - | - | - | - | - | - | - | - |
| ED | - | <0.01 | - | <0.01 | - | - | - | - | - | - | - | - | - | - | - | - | - | - | - | - | - |
| EE | 0.76 | 0.66 | 0.64 | 0.52 | 0.49 | 0.46 | 0.42 | 0.37 | 0.36 | 0.34 | 0.34 | 0.34 | 0.37 | 0.35 | 0.33 | 0.32 | 0.32 | 0.31 | 0.28 | 0.27 | 0.57 |
| **Malta** | - | - | - | - | - | - | - | - | - | - | **0.20** | **0.20** | **0.18** | **0.19** | **0.29** | **0.20** | **0.19** | **0.27** | **0.24** | **0.26** | **0.38** |
| EA | - | - | - | - | - | - | - | - | - | - | 0.01 | <0.01 | <0.01 | 0.01 | 0.01 | <0.01 | <0.01 | <0.01 | <0.01 | <0.01 | 0.01 |
| EB | - | - | - | - | - | - | - | - | - | - | - | - | - | - | - | - | - | - | - | - | - |
| EC | - | - | - | - | - | - | - | - | - | - | - | 0.01 | 0.01 | 0.01 | 0.02 | <0.01 | - | 0.01 | - | - | 0.13 |
| ED | - | - | - | - | - | - | - | - | - | - | - | - | - | - | - | - | - | - | - | - | - |
| EE | - | - | - | - | - | - | - | - | - | - | 0.19 | 0.19 | 0.17 | 0.17 | 0.27 | 0.20 | 0.19 | 0.26 | 0.24 | 0.25 | 0.25 |
| Country, community consumption of sulfonamides and trimethoprim (J01E); EA, consumption of trimethoprim and derivatives (J01EA); EB, consumption of short-acting sulfonamides (J01EB); EC, consumption of intermediate-acting sulfonamides (J01EC); ED, consumption of long-acting sulfonamides (J01ED); EE, consumption of combinations of sulfonamides and trimethoprim (J01EE); **-**, no consumption reported; Numbers reported in *italic* are total care data, i.e. community and hospital sector combined; ^a^Data for Romania have a coverage in 2009 limited to 30-40%; ^b^Data for Spain include private prescriptions from 2016 onwards. | | | | | | | | | | | | | | | | | | | | | |
| **Netherlands** | **0.77** | **0.75** | **0.76** | **0.71** | **0.70** | **0.68** | **0.67** | **0.65** | **0.63** | **0.62** | **0.58** | **0.58** | **0.56** | **0.55** | **0.54** | **0.52** | **0.47** | **0.44** | **0.43** | **0.42** | **0.42** |
| EA | 0.28 | 0.28 | 0.30 | 0.28 | 0.28 | 0.27 | 0.27 | 0.26 | 0.25 | 0.24 | 0.22 | 0.22 | 0.21 | 0.20 | 0.20 | 0.19 | 0.17 | 0.16 | 0.14 | 0.14 | 0.13 |
| EB | <0.01 | <0.01 | <0.01 | <0.01 | <0.01 | <0.01 | - | - | - | - | - | - | - | - | - | - | - | - | - | - | - |
| EC | <0.01 | <0.01 | <0.01 | <0.01 | <0.01 | 0.01 | <0.01 | <0.01 | <0.01 | <0.01 | <0.01 | <0.01 | <0.01 | <0.01 | <0.01 | <0.01 | <0.01 | <0.01 | <0.01 | <0.01 | <0.01 |
| ED | - | - | - | - | - | - | - | - | - | - | - | - | - | - | - | - | - | - | - | - | - |
| EE | 0.48 | 0.46 | 0.46 | 0.43 | 0.42 | 0.40 | 0.39 | 0.39 | 0.38 | 0.38 | 0.36 | 0.36 | 0.35 | 0.35 | 0.34 | 0.33 | 0.29 | 0.28 | 0.28 | 0.28 | 0.29 |
| **Norway** | - | **1.27** | - | - | **1.11** | **1.09** | **1.03** | **1.05** | **1.02** | **0.81** | **0.79** | **0.77** | **0.73** | **0.70** | **0.69** | **0.68** | **0.67** | **0.68** | **0.67** | **0.66** | **0.65** |
| EA | - | 0.81 | - | - | 0.77 | 0.73 | 0.70 | 0.73 | 0.71 | 0.56 | 0.54 | 0.51 | 0.48 | 0.46 | 0.44 | 0.42 | 0.40 | 0.37 | 0.34 | 0.31 | 0.29 |
| EB | - | <0.01 | - | - | <0.01 | <0.01 | - | - | - | - | - | - | - | - | - | - | - | - | - | - | - |
| EC | - | <0.01 | - | - | - | - | - | - | - | - | - | - | - | - | - | - | - | - | - | <0.01 | <0.01 |
| ED | - | - | - | - | - | - | - | - | - | - | - | - | - | - | - | - | - | - | - | - | - |
| EE | - | 0.45 | - | - | 0.34 | 0.35 | 0.33 | 0.32 | 0.31 | 0.26 | 0.26 | 0.26 | 0.25 | 0.24 | 0.25 | 0.26 | 0.27 | 0.31 | 0.33 | 0.34 | 0.36 |
| **Poland** | - | **2.72** | **2.99** | **2.60** | **2.62** | **1.25** | - | **0.59** | **0.59** | - | **1.04** | **0.96** | **0.95** | **0.09** | **0.10** | **1.50** | **0.57** | **0.54** | **0.58** | **0.56** | **0.48** |
| EA | - | <0.01 | <0.01 | 0.02 | 0.03 | 0.04 | - | <0.01 | <0.01 | - | <0.01 | <0.01 | <0.01 | <0.01 | <0.01 | 0.05 | 0.06 | 0.06 | 0.07 | 0.08 | 0.08 |
| EB | - | - | - | - | - | - | - | - | - | - | - | - | - | - | - | - | - | - | - | - | - |
| EC | - | - | - | - | - | - | - | - | - | - | - | - | - | - | - | - | - | <0.01 | 0.01 | 0.01 | <0.01 |
| ED | - | - | - | - | - | <0.01 | - | - | - | - | - | - | - | - | - | - | - | - | - | - | - |
| EE | - | 2.72 | 2.99 | 2.58 | 2.59 | 1.21 | - | 0.59 | 0.58 | - | 1.04 | 0.96 | 0.94 | 0.09 | 0.10 | 1.45 | 0.51 | 0.47 | 0.51 | 0.48 | 0.40 |
| **Portugal** | **1.08** | **1.05** | **1.03** | **0.99** | **0.90** | **0.88** | **1.24** | **0.87** | **0.58** | **0.51** | - | **0.44** | **0.43** | **0.47** | **0.70** | **0.53** | **0.44** | **0.44** | **0.43** | **0.42** | **0.41** |
| EA | - | - | - | - | - | - | - | - | - | - | - | - | - | - | - | - | - | - | - | - | - |
| EB | - | - | - | - | - | - | - | - | - | - | - | - | - | - | - | - | - | - | - | - | - |
| EC | 0.01 | 0.01 | 0.02 | 0.02 | 0.02 | 0.03 | 0.03 | 0.03 | 0.02 | 0.03 | - | 0.01 | <0.01 | <0.01 | <0.01 | <0.01 | <0.01 | <0.01 | <0.01 | <0.01 | <0.01 |
| ED | - | - | - | - | - | - | - | - | - | - | - | - | - | - | - | - | - | - | - | - | - |
| EE | 1.08 | 1.04 | 1.02 | 0.97 | 0.88 | 0.85 | 1.21 | 0.84 | 0.56 | 0.48 | - | 0.43 | 0.43 | 0.46 | 0.70 | 0.52 | 0.44 | 0.44 | 0.42 | 0.42 | 0.41 |
| **Romania^a^** | - | - | - | - | - | - | - | - | - | - | - | - | ***0.16*** | - | ***0.96*** | ***0.89*** | ***0.94*** | ***0.88*** | ***0.89*** | ***0.81*** | ***0.88*** |
| EA | - | - | - | - | - | - | - | - | - | - | - | - | *-* | - | *-* | *-* | *-* | *-* | *-* | *-* | *-* |
| EB | - | - | - | - | - | - | - | - | - | - | - | - | *-* | - | *0.01* | *0.01* | *0.01* | *0.01* | *0.01* | *0.01* | *<0.01* |
| EC | - | - | - | - | - | - | - | - | - | - | - | - | *-* | - | *-* | *-* | *-* | *-* | *-* | *-* | *-* |
| ED | - | - | - | - | - | - | - | - | - | - | - | - | *-* | - | *-* | *-* | *-* | *-* | *-* | *-* | *-* |
| EE | - | - | - | - | - | - | - | - | - | - | - | - | *0.16* | - | *0.95* | *0.89* | *0.93* | *0.87* | *0.88* | *0.81* | *0.87* |
| Country, community consumption of sulfonamides and trimethoprim (J01E); EA, consumption of trimethoprim and derivatives (J01EA); EB, consumption of short-acting sulfonamides (J01EB); EC, consumption of intermediate-acting sulfonamides (J01EC); ED, consumption of long-acting sulfonamides (J01ED); EE, consumption of combinations of sulfonamides and trimethoprim (J01EE); **-**, no consumption reported; Numbers reported in *italic* are total care data, i.e. community and hospital sector combined; ^a^Data for Romania have a coverage in 2009 limited to 30-40%; ^b^Data for Spain include private prescriptions from 2016 onwards. | | | | | | | | | | | | | | | | | | | | | |
| **Slovakia** | - | - | **1.70** | **1.33** | **1.22** | **1.04** | **0.85** | **0.79** | **0.72** | **0.60** | **0.52** | **0.48** | **0.43** | - | ***0.40*** | **0.34** | **0.38** | **0.41** | **0.45** | **1.19** | - |
| EA | - | - | 0.06 | 0.06 | 0.06 | 0.06 | 0.05 | 0.05 | 0.05 | 0.04 | 0.04 | 0.04 | 0.03 | - | *0.03* | 0.02 | 0.02 | 0.03 | 0.02 | - | - |
| EB | - | - | <0.01 | <0.01 | - | - | - | - | - | - | - | - | - | - | *-* | - | - | - | - | - | - |
| EC | - | - | - | - | - | - | - | - | - | - | - | - | - | - | *-* | - | - | - | - | - | - |
| ED | - | - | - | - | - | - | - | - | - | - | - | - | - | - | *-* | - | - | - | - | - | - |
| EE | - | - | 1.64 | 1.27 | 1.16 | 0.98 | 0.80 | 0.74 | 0.67 | 0.56 | 0.48 | 0.44 | 0.40 | - | *0.38* | 0.32 | 0.36 | 0.38 | 0.43 | 1.19 | - |
| **Slovenia** | **1.22** | **1.17** | **1.14** | **1.20** | **1.21** | **1.09** | **1.15** | **1.24** | **1.21** | **1.16** | **1.16** | **1.13** | **1.06** | **1.06** | **0.99** | **0.94** | **0.90** | **0.82** | **0.79** | - | - |
| EA | - | - | - | - | - | - | - | - | - | - | - | - | - | - | - | - | - | - | - | - | - |
| EB | - | - | - | - | - | - | - | - | - | - | - | - | - | - | - | - | - | - | - | - | - |
| EC | - | - | - | - | - | - | - | - | - | - | - | - | - | - | - | - | - | - | - | - | - |
| ED | - | - | - | - | - | - | - | - | - | - | - | - | - | - | - | - | - | - | - | - | - |
| EE | 1.22 | 1.17 | 1.14 | 1.20 | 1.21 | 1.09 | 1.15 | 1.24 | 1.21 | 1.16 | 1.16 | 1.13 | 1.06 | 1.06 | 0.99 | 0.94 | 0.90 | 0.82 | 0.79 | - | - |
| **Spain^b^** | **0.77** | **0.60** | **0.41** | **0.38** | **0.35** | **0.34** | **0.33** | **0.31** | **0.30** | **0.29** | **0.29** | **0.30** | **0.30** | **0.27** | **0.27** | **0.26** | **0.27** | **0.27** | **0.27** | **0.26** | **0.42** |
| EA | <0.01 | <0.01 | <0.01 | <0.01 | <0.01 | <0.01 | <0.01 | <0.01 | <0.01 | <0.01 | <0.01 | <0.01 | <0.01 | <0.01 | <0.01 | <0.01 | <0.01 | <0.01 | <0.01 | <0.01 | <0.01 |
| EB | <0.01 | <0.01 | - | - | - | - | - | - | - | - | - | - | - | - | - | - | - | - | - | - | - |
| EC | 0.04 | 0.03 | 0.03 | 0.02 | 0.02 | 0.02 | 0.02 | 0.01 | 0.01 | 0.01 | 0.01 | 0.01 | 0.01 | 0.01 | 0.01 | 0.01 | 0.01 | 0.01 | 0.01 | 0.01 | <0.01 |
| ED | - | - | - | - | - | - | - | - | - | - | - | - | - | - | - | - | - | - | - | - | - |
| EE | 0.72 | 0.57 | 0.38 | 0.35 | 0.33 | 0.32 | 0.32 | 0.30 | 0.28 | 0.28 | 0.28 | 0.28 | 0.29 | 0.26 | 0.26 | 0.25 | 0.26 | 0.26 | 0.26 | 0.25 | 0.42 |
| **Sweden** | **0.75** | **0.75** | **0.78** | **0.76** | **0.75** | **0.74** | **0.72** | **0.70** | **0.67** | **0.67** | **0.75** | **0.57** | **0.52** | **0.36** | **0.46** | **0.44** | **0.42** | **0.40** | **0.40** | **0.38** | **0.27** |
| EA | 0.57 | 0.57 | 0.60 | 0.60 | 0.59 | 0.58 | 0.56 | 0.53 | 0.49 | 0.49 | 0.43 | 0.36 | 0.29 | 0.25 | 0.22 | 0.20 | 0.17 | 0.15 | 0.14 | 0.12 | 0.11 |
| EB | - | - | - | - | - | - | - | - | - | - | - | - | - | - | - | - | - | - | - | - | - |
| EC | - | - | - | - | - | - | - | - | - | - | <0.01 | <0.01 | <0.01 | <0.01 | <0.01 | <0.01 | <0.01 | - | - | - | - |
| ED | - | - | - | - | - | - | - | - | - | - | - | - | - | - | - | - | - | - | - | - | - |
| EE | 0.18 | 0.18 | 0.17 | 0.16 | 0.16 | 0.16 | 0.16 | 0.17 | 0.18 | 0.19 | 0.32 | 0.21 | 0.23 | 0.11 | 0.24 | 0.24 | 0.25 | 0.25 | 0.26 | 0.26 | 0.16 |
| **UK** | **1.17** | **1.13** | **1.09** | **1.08** | **1.09** | **1.06** | **1.06** | **1.08** | **1.07** | **1.05** | **1.08** | **1.13** | **1.18** | **1.24** | **1.25** | **1.33** | **1.48** | **1.49** | **1.32** | **1.22** | **1.04** |
| EA | 1.05 | 1.04 | 1.01 | 1.00 | 1.01 | 1.01 | 1.03 | 1.04 | 1.04 | 1.04 | 1.07 | 1.13 | 1.17 | 1.19 | 1.20 | 1.22 | 1.28 | 1.28 | 1.19 | 1.10 | 0.92 |
| EB | <0.01 | <0.01 | <0.01 | <0.01 | <0.01 | <0.01 | <0.01 | <0.01 | <0.01 | <0.01 | <0.01 | <0.01 | <0.01 | <0.01 | <0.01 | <0.01 | <0.01 | <0.01 | <0.01 | <0.01 | <0.01 |
| EC | <0.01 | <0.01 | <0.01 | <0.01 | <0.01 | <0.01 | <0.01 | <0.01 | <0.01 | <0.01 | <0.01 | <0.01 | <0.01 | <0.01 | <0.01 | <0.01 | <0.01 | <0.01 | 0.01 | <0.01 | <0.01 |
| ED | 0.06 | 0.05 | 0.05 | 0.04 | 0.04 | 0.02 | <0.01 | <0.01 | <0.01 | - | <0.01 | <0.01 | <0.01 | <0.01 | <0.01 | <0.01 | <0.01 | <0.01 | - | - | - |
| EE | 0.05 | 0.04 | 0.03 | 0.03 | 0.03 | 0.03 | 0.03 | 0.03 | 0.03 | <0.01 | <0.01 | <0.01 | <0.01 | 0.04 | 0.05 | 0.10 | 0.20 | 0.21 | 0.12 | 0.12 | 0.13 |

Country, community consumption of sulfonamides and trimethoprim (J01E); EA, consumption of trimethoprim and derivatives (J01EA); EB, consumption of short-acting sulfonamides (J01EB); EC, consumption of intermediate-acting sulfonamides (J01EC); ED, consumption of long-acting sulfonamides (J01ED); EE, consumption of combinations of sulfonamides and trimethoprim (J01EE); **-**, no consumption reported; Numbers reported in *italic* are total care data, i.e. community and hospital sector combined; ^a^Data for Romania have a coverage in 2009 limited to 30-40%; ^b^Data for Spain include private prescriptions from 2016 onwards.

**Table S4. Consumption of sulfonamides and trimethoprim (ATC J01E) in the community, expressed in packages per 1000 inhabitants per day, 23 EU/EEA countries, 2006-2017.**

| **Country** | **2006** | **2007** | **2008** | **2009** | **2010** | **2011** | **2012** | **2013** | **2014** | **2015** | **2016** | **2017** |  |
| --- | --- | --- | --- | --- | --- | --- | --- | --- | --- | --- | --- | --- | --- |
| **Austria** | - | **0.03** | **0.03** | **0.03** | **0.03** | **0.03** | **0.03** | **0.03** | **0.02** | **0.02** | **0.02** | **0.02** |  |
| EA | - | 0.02 | 0.02 | 0.02 | 0.02 | 0.02 | 0.02 | 0.02 | 0.01 | 0.01 | 0.01 | 0.01 |  |
| EB | - | - | - | - | - | - | - | - | - | - | - | - |  |
| EC | - | - | - | - | - | - | - | - | - | - | - | - |  |
| ED | - | - | - | - | - | - | - | - | - | - | - | - |  |
| EE | - | 0.01 | 0.01 | 0.01 | 0.01 | 0.01 | 0.01 | 0.01 | 0.01 | 0.01 | 0.01 | 0.01 |  |
| **Belgium^a^** | - | **0.05** | **0.05** | **0.05** | **0.05** | **0.05** | **0.05** | **0.04** | **0.04** | **0.04** | **0.06** | **0.05** |  |
| EA | - | - | - | - | - | - | - | - | - | - | - | - |  |
| EB | - | - | - | - | - | - | - | - | - | - | - | - |  |
| EC | - | - | - | - | - | - | - | - | - | - | - | - |  |
| ED | - | - | - | - | - | - | - | - | - | - | - | - |  |
| EE | - | 0.05 | 0.05 | 0.05 | 0.05 | 0.05 | 0.05 | 0.04 | 0.04 | 0.04 | 0.06 | 0.05 |  |
| **Bulgaria** | **0.25** | **0.25** | **0.26** | **0.23** | **0.24** | **0.24** | **0.23** | **0.23** | **0.22** | **0.21** | **0.21** | **0.19** |  |
| EA | - | - | - | - | - | - | - | - | - | - | - | - |  |
| EB | - | - | - | - | - | - | - | - | - | - | - | - |  |
| EC | - | - | - | - | - | - | - | - | - | - | - | - |  |
| ED | - | - | - | - | - | - | - | - | - | - | - | - |  |
| EE | 0.25 | 0.25 | 0.26 | 0.23 | 0.24 | 0.24 | 0.23 | 0.23 | 0.22 | 0.21 | 0.21 | 0.19 |  |
| **Croatia** | - | **0.28** | **0.23** | **0.20** | **0.17** | **0.14** | **0.13** | **0.13** | **0.12** | **0.12** | **0.09** | **0.07** |  |
| EA | - | - | - | - | - | - | - | - | - | - | - | - |  |
| EB | - | - | - | - | - | - | - | - | - | - | - | - |  |
| EC | - | - | - | - | - | - | - | - | - | - | - | - |  |
| ED | - | - | - | - | - | - | - | - | - | - | - | - |  |
| EE | - | 0.28 | 0.23 | 0.20 | 0.17 | 0.14 | 0.13 | 0.13 | 0.12 | 0.12 | 0.09 | 0.07 |  |
| **Czechia** | - | **0.22** | - | - | **0.17** | **0.16** | **0.14** | **0.15** | **0.14** | **0.15** | - | - |  |
| EA | - | 0.04 | - | - | 0.03 | 0.02 | 0.02 | 0.02 | 0.02 | 0.02 | - | - |  |
| EB | - | - | - | - | - | - | - | - | - | - | - | - |  |
| EC | - | - | - | - | - | - | - | - | - | - | - | - |  |
| ED | - | - | - | - | - | - | - | - | - | - | - | - |  |
| EE | - | 0.18 | - | - | 0.14 | 0.14 | 0.12 | 0.13 | 0.12 | 0.13 | - | - |  |
| Country, communityl consumption of sulfonamides and trimethoprim (J01E); EA, consumption of trimethoprim and derivatives (J01EA); EB, consumption of short-acting sulfonamides (J01EB); EC, consumption of intermediate-acting sulfonamides (J01EC); ED, consumption of long-acting sulfonamides (J01ED); EE, consumption of combinations of sulfonamides and trimethoprim (J01EE); **-**, no consumption reported; Numbers reported in *italic* are total care data, i.e. community and hospital sector combined; ^a^Data for Belgium are slightly overestimated from 2016 onwards (nursing homes counting units versus packages before 2016); ^b^Data for the Netherlands are based on average package size; ^c^Data for Spain include private prescriptions from 2016 onwards. | | | | | | | | | | | | | |
| **Denmark** | - | **0.14** | **0.13** | **0.13** | **0.13** | **0.12** | **0.12** | **0.11** | **0.11** | **0.10** | **0.09** | **0.09** |  |
| EA | - | 0.03 | 0.03 | 0.03 | 0.03 | 0.03 | 0.03 | 0.03 | 0.04 | 0.04 | 0.04 | 0.04 |  |
| EB | - | 0.11 | 0.10 | 0.10 | 0.09 | 0.09 | 0.08 | 0.08 | 0.08 | 0.06 | 0.06 | 0.05 |  |
| EC | - | - | - | - | - | - | - | - | - | - | - | - |  |
| ED | - | - | - | - | - | - | - | - | - | - | - | - |  |
| EE | - | - | <0.01 | - | - | - | <0.01 | - | - | - | - | - |  |
| **Estonia** | **0.10** | **0.10** | **0.09** | **0.08** | **0.07** | **0.07** | **0.06** | **0.06** | **0.06** | **0.06** | **0.05** | **0.05** |  |
| EA | <0.01 | <0.01 | <0.01 | <0.01 | <0.01 | <0.01 | <0.01 | <0.01 | <0.01 | <0.01 | <0.01 | <0.01 |  |
| EB | - | - | - | - | - | - | - | - | - | - | - | - |  |
| EC | - | - | - | - | - | - | - | - | - | - | - | - |  |
| ED | <0.01 | - | - | - | - | - | - | - | - | - | - | - |  |
| EE | 0.10 | 0.09 | 0.08 | 0.08 | 0.07 | 0.06 | 0.06 | 0.06 | 0.06 | 0.05 | 0.05 | 0.05 |  |
| **Finland** | - | - | **0.08** | **0.08** | **0.09** | **0.16** | **0.14** | **0.14** | **0.14** | **0.13** | **0.13** | **0.12** |  |
| EA | - | - | 0.08 | 0.08 | 0.09 | 0.09 | 0.08 | 0.08 | 0.08 | 0.08 | 0.08 | 0.07 |  |
| EB | - | - | - | - | - | - | - | - | - | - | - | - |  |
| EC | - | - | - | - | - | - | - | - | - | - | - | - |  |
| ED | - | - | - | - | - | - | - | - | - | - | - | - |  |
| EE | - | - | - | - | - | 0.07 | 0.06 | 0.06 | 0.06 | 0.05 | 0.05 | 0.05 |  |
| **France** | - | - | - | - | **0.09** | **0.09** | **0.10** | **0.06** | **0.08** | **0.08** | **0.08** | **0.09** |  |
| EA | - | - | - | - | - | - | - | - | - | - | - | <0.01 |  |
| EB | - | - | - | - | <0.01 | <0.01 | <0.01 | <0.01 | <0.01 | <0.01 | <0.01 | <0.01 |  |
| EC | - | - | - | - | <0.01 | - | - | <0.01 | <0.01 | <0.01 | <0.01 | <0.01 |  |
| ED | - | - | - | - | - | - | - | - | - | - | - | - |  |
| EE | - | - | - | - | 0.08 | 0.08 | 0.09 | 0.06 | 0.08 | 0.08 | 0.08 | 0.08 |  |
| **Greece** | ***0.10*** | ***0.09*** | ***0.09*** | **0.07** | ***0.07*** | **0.07** | **0.07** | **0.07** | **0.07** | **0.08** | **0.07** | **0.07** |  |
| EA | *-* | *-* | *-* | - | *-* | - | - | - | - | - | - | - |  |
| EB | *-* | *-* | *-* | - | *-* | - | - | - | - | - | - | - |  |
| EC | *-* | *-* | *-* | - | *-* | - | <0.01 | - | <0.01 | <0.01 | <0.01 | <0.01 |  |
| ED | *-* | *-* | *-* | - | *-* | - | - | - | - | - | - | - |  |
| EE | *0.10* | *0.09* | *0.09* | 0.07 | *0.07* | 0.07 | 0.07 | 0.07 | 0.07 | 0.08 | 0.07 | 0.07 |  |
| Country, community consumption of sulfonamides and trimethoprim (J01E); EA, consumption of trimethoprim and derivatives (J01EA); EB, consumption of short-acting sulfonamides (J01EB); EC, consumption of intermediate-acting sulfonamides (J01EC); ED, consumption of long-acting sulfonamides (J01ED); EE, consumption of combinations of sulfonamides and trimethoprim (J01EE); **-**, no consumption reported; Numbers reported in *italic* are total care data, i.e. community and hospital sector combined; ^a^Data for Belgium are slightly overestimated from 2016 onwards (nursing homes counting units versus packages before 2016); ^b^Data for the Netherlands are based on average package size; ^c^Data for Spain include private prescriptions from 2016 onwards. | | | | | | | | | | | | | |
| **Iceland** | - | - | - | - | ***0.15*** | ***0.14*** | ***0.15*** | ***0.12*** | **0.11** | **0.09** | **0.08** | **0.07** |  |
| EA | - | - | - | - | *0.04* | *0.05* | *0.05* | *0.04* | 0.04 | 0.03 | 0.02 | 0.02 |  |
| EB | - | - | - | - | *-* | *-* | *-* | *-* | - | - | - | - |  |
| EC | - | - | - | - | *-* | *-* | *-* | *-* | - | - | - | <0.01 |  |
| ED | - | - | - | - | *-* | *-* | *-* | *-* | - | - | - | - |  |
| EE | - | - | - | - | *0.11* | *0.10* | *0.10* | *0.08* | 0.07 | 0.06 | 0.06 | 0.06 |  |
| **Ireland** |  | **0.03** |  |  | **0.04** | **0.04** | **0.04** | **0.04** | **0.04** | **0.04** | **0.04** | **0.04** |  |
| EA | - | 0.02 | - | - | 0.03 | 0.03 | 0.03 | 0.03 | 0.03 | 0.03 | 0.03 | 0.03 |  |
| EB | - | - | - | - | - | - | - | - | - | - | - | - |  |
| EC | - | - | - | - | - | - | - | - | - | - | - | - |  |
| ED | - | - | - | - | - | - | - | - | - | - | - | - |  |
| EE | - | 0.01 | - | - | 0.01 | 0.01 | 0.01 | 0.01 | 0.01 | 0.01 | 0.01 | 0.01 |  |
| **Italy** | - | - | **0.07** | - | **0.06** | **0.06** | **0.06** | **0.05** | **0.05** | **0.05** | **0.05** | **0.05** |  |
| EA | - | - | - | - | - | - | - | - | - | - | - | - |  |
| EB | - | - | - | - | - | - | - | - | - | - | - | - |  |
| EC | - | - | <0.01 | - | <0.01 | <0.01 | - | - | - | - | - | - |  |
| ED | - | - | - | - | - | - | - | - | - | - | - | - |  |
| EE | - | - | 0.07 | - | 0.06 | 0.06 | 0.06 | 0.05 | 0.05 | 0.05 | 0.05 | 0.05 |  |
| **Latvia** | - | - | - | - | **0.17** | **0.17** | **0.16** | **0.16** | **0.14** | **0.14** | **0.14** | **0.14** |  |
| EA | - | - | - | - | <0.01 | <0.01 | <0.01 | <0.01 | <0.01 | <0.01 | <0.01 | <0.01 |  |
| EB | - | - | - | - | - | - | - | - | - | - | - | - |  |
| EC | - | - | - | - | - | - | - | - | - | - | - | - |  |
| ED | - | - | - | - | - | - | - | - | - | - | - | - |  |
| EE | - | - | - | - | 0.17 | 0.17 | 0.16 | 0.16 | 0.14 | 0.14 | 0.14 | 0.14 |  |
| **Lithuania** | *-* | ***<0.01*** | ***<0.01*** | ***<0.01*** | ***0.07*** | ***0.07*** | **0.06** | **0.06** | **0.05** | **0.05** | **0.05** | **0.05** |  |
| EA | *-* | *<0.01* | *<0.01* | *<0.01* | *<0.01* | *<0.01* | <0.01 | <0.01 | <0.01 | <0.01 | <0.01 | <0.01 |  |
| EB | *-* | *-* | *-* | *-* | *-* | *-* | - | - | - | - | - | - |  |
| EC | *-* | *-* | *-* | *-* | *-* | *-* | - | - | - | - | - | - |  |
| ED | *-* | *-* | *-* | *-* | *-* | *-* | - | - | - | - | - | - |  |
| EE | *-* | *-* | *-* | *-* | *0.07* | *0.07* | 0.05 | 0.06 | 0.05 | 0.05 | 0.05 | 0.05 |  |
| Country, community consumption of sulfonamides and trimethoprim (J01E); EA, consumption of trimethoprim and derivatives (J01EA); EB, consumption of short-acting sulfonamides (J01EB); EC, consumption of intermediate-acting sulfonamides (J01EC); ED, consumption of long-acting sulfonamides (J01ED); EE, consumption of combinations of sulfonamides and trimethoprim (J01EE); **-**, no consumption reported; Numbers reported in *italic* are total care data, i.e. community and hospital sector combined; ^a^Data for Belgium are slightly overestimated from 2016 onwards (nursing homes counting units versus packages before 2016); ^b^Data for the Netherlands are based on average package size; ^c^Data for Spain include private prescriptions from 2016 onwards. | | | | | | | | | | | | | |
| **Luxembourg** | **0.06** | **0.06** | **0.06** | **0.06** | **0.07** | **0.06** | **0.06** | **0.06** | **0.06** | **0.05** | **0.06** | - |  |
| EA | - | - | - | - | - | - | - | - | - | - | - | - |  |
| EB | - | - | - | - | - | - | - | - | - | - | - | - |  |
| EC | - | - | - | - | - | - | - | - | - | - | - | - |  |
| ED | - | - | - | - | - | - | - | - | - | - | - | - |  |
| EE | 0.06 | 0.06 | 0.06 | 0.06 | 0.07 | 0.06 | 0.06 | 0.06 | 0.06 | 0.05 | 0.06 | - |  |
| **Netherlands^b^** | - | - | **0.10** | **0.10** | - | - | - | - | - | - | - | - |  |
| EA | - | - | 0.03 | 0.03 | - | - | - | - | - | - | - | - |  |
| EB | - | - | - | - | - | - | - | - | - | - | - | - |  |
| EC | - | - | <0.01 | <0.01 | - | - | - | - | - | - | - | - |  |
| ED | - | - | - | - | - | - | - | - | - | - | - | - |  |
| EE | - | - | 0.07 | 0.07 | - | - | - | - | - | - | - | - |  |
| **Portugal** | - | - | **0.08** | **0.07** | **0.08** | **0.09** | **0.09** | **0.06** | **0.06** | **0.06** | **0.06** | **0.06** |  |
| EA | - | - | - | - | - | - | - | - | - | - | - | - |  |
| EB | - | - | - | - | - | - | - | - | - | - | - | - |  |
| EC | - | - | <0.01 | <0.01 | <0.01 | <0.01 | <0.01 | - | <0.01 | <0.01 | <0.01 | <0.01 |  |
| ED | - | - | - | - | - | - | - | - | - | - | - | - |  |
| EE | - | - | 0.08 | 0.07 | 0.08 | 0.09 | 0.09 | 0.06 | 0.06 | 0.06 | 0.06 | 0.06 |  |
| **Slovakia** | - | - | - | - | - | **0.09** | **0.07** | **0.08** | **0.09** | **0.07** | **0.05** | - |  |
| EA | - | - | - | - | - | *<0.01* | <0.01 | <0.01 | <0.01 | <0.01 | - | - |  |
| EB | - | - | - | - | - | *-* | - | - | - | - | - | - |  |
| EC | - | - | - | - | - | *-* | - | - | - | - | - | - |  |
| ED | - | - | - | - | - | *-* | - | - | - | - | - | - |  |
| EE | - | - | - | - | - | *0.08* | 0.07 | 0.08 | 0.08 | 0.07 | 0.05 | - |  |
| **Slovenia** | - | **0.25** | **0.23** | **0.22** | **0.22** | **0.21** | **0.20** | **0.19** | **0.17** | **0.16** | **0.15** | **0.14** |  |
| EA | - | - | - | - | - | - | - | - | - | - | - | - |  |
| EB | - | - | - | - | - | - | - | - | - | - | - | - |  |
| EC | - | - | - | - | - | - | - | - | - | - | - | - |  |
| ED | - | - | - | - | - | - | - | - | - | - | - | - |  |
| EE | - | 0.25 | 0.23 | 0.22 | 0.22 | 0.21 | 0.20 | 0.19 | 0.17 | 0.16 | 0.15 | 0.14 |  |
| Country, community consumption of sulfonamides and trimethoprim (J01E); EA, consumption of trimethoprim and derivatives (J01EA); EB, consumption of short-acting sulfonamides (J01EB); EC, consumption of intermediate-acting sulfonamides (J01EC); ED, consumption of long-acting sulfonamides (J01ED); EE, consumption of combinations of sulfonamides and trimethoprim (J01EE); **-**, no consumption reported; Numbers reported in *italic* are total care data, i.e. community and hospital sector combined; ^a^Data for Belgium are slightly overestimated from 2016 onwards (nursing homes counting units versus packages before 2016); ^b^Data for the Netherlands are based on average package size; ^c^Data for Spain include private prescriptions from 2016 onwards. | | | | | | | | | | | | | |
| **Spain^c^** | - | - | - | - | **0.03** | **0.03** | **0.02** | **0.02** | **0.03** | **0.02** | **0.04** | **0.04** |  |
| EA | - | - | - | - | <0.01 | <0.01 | <0.01 | <0.01 | <0.01 | <0.01 | <0.01 | <0.01 |  |
| EB | - | - | - | - | - | - | - | - | - | - | - | - |  |
| EC | - | - | - | - | <0.01 | <0.01 | <0.01 | <0.01 | <0.01 | <0.01 | <0.01 | <0.01 |  |
| ED | - | - | - | - | - | - | - | - | - | - | - | - |  |
| EE | - | - | - | - | 0.02 | 0.02 | 0.02 | 0.02 | 0.02 | 0.02 | 0.04 | 0.04 |  |
| **Sweden** | - | - | - | **0.06** | **0.06** | **0.05** | **0.05** | **0.05** | **0.04** | **0.04** | **0.04** | **0.04** |  |
| EA | - | - | - | 0.04 | 0.03 | 0.03 | 0.03 | 0.02 | 0.02 | 0.02 | 0.01 | 0.01 |  |
| EB | - | - | - | - | - | - | - | - | - | - | - | - |  |
| EC | - | - | - | <0.01 | <0.01 | <0.01 | <0.01 | <0.01 | - | - | - | - |  |
| ED | - | - | - | - | - | - | - | - | - | - | - | - |  |
| EE | - | - | - | 0.02 | 0.02 | 0.02 | 0.02 | 0.02 | 0.02 | 0.02 | 0.03 | 0.03 |  |
| **UK** | - | - | - | - | - | - | - | - | - | **0.01** | - | - |  |
| EA | - | - | - | - | - | - | - | - | - | 0.01 | - | - |  |
| EB | - | - | - | - | - | - | - | - | - | - | - | - |  |
| EC | - | - | - | - | - | - | - | - | - | - | - | - |  |
| ED | - | - | - | - | - | - | - | - | - | - | - | - |  |
| EE | - | - | - | - | - | - | - | - | - | <0.01 | - | - |  |

Country, community consumption of sulfonamides and trimethoprim (J01E); EA, consumption of trimethoprim and derivatives (J01EA); EB, consumption of short-acting sulfonamides (J01EB);
EC, consumption of intermediate-acting sulfonamides (J01EC); ED, consumption of long-acting sulfonamides (J01ED); EE, consumption of combinations of sulfonamides and trimethoprim
(J01EE); **-**, no consumption reported; Numbers reported in *italic* are total care data, i.e. community and hospital sector combined; ^a^Data for Belgium are slightly overestimated from 2016 onwards (nursing homes
counting units versus packages before 2016); ^b^Data for the Netherlands are based on average package size; ^c^Data for Spain include private prescriptions from 2016 onwards.

**Table S5. Consumption of other antibacterials (ATC J01X) in the community, expressed in DDD (ATC/DDD index 2019) per 1000 inhabitants per day, 30 EU/EEA countries, 1997-2017.**

| **Country** | **1997** | **1998** | **1999** | **2000** | **2001** | **2002** | **2003** | **2004** | **2005** | **2006** | **2007** | **2008** | **2009** | **2010** | **2011** | **2012** | **2013** | **2014** | **2015** | **2016** | **2017** |
| --- | --- | --- | --- | --- | --- | --- | --- | --- | --- | --- | --- | --- | --- | --- | --- | --- | --- | --- | --- | --- | --- |
| **Austria** | - | **0.01** | **0.03** | **0.03** | **0.03** | **0.03** | **0.02** | **0.05** | **0.13** | **0.15** | **0.16** | **0.18** | **0.20** | **0.23** | **0.24** | **0.28** | **0.35** | **0.33** | **0.33** | **0.34** | **0.38** |
| XA | - | <0.01 | <0.01 | <0.01 | <0.01 | <0.01 | <0.01 | <0.01 | <0.01 | <0.01 | <0.01 | <0.01 | <0.01 | <0.01 | <0.01 | <0.01 | <0.01 | <0.01 | <0.01 | <0.01 | <0.01 |
| XB | - | <0.01 | <0.01 | - | - | - | - | <0.01 | - | - | - | - | - | <0.01 | <0.01 | <0.01 | 0.01 | 0.01 | 0.01 | 0.01 | 0.01 |
| XC | - | 0.01 | 0.01 | 0.02 | 0.02 | 0.02 | 0.01 | 0.03 | 0.03 | 0.03 | 0.03 | 0.03 | 0.03 | 0.04 | 0.04 | 0.03 | 0.04 | 0.03 | 0.03 | 0.03 | 0.02 |
| XD | - | <0.01 | <0.01 | <0.01 | <0.01 | <0.01 | <0.01 | <0.01 | <0.01 | <0.01 | <0.01 | <0.01 | <0.01 | <0.01 | <0.01 | <0.01 | <0.01 | <0.01 | <0.01 | <0.01 | <0.01 |
| XE | - | - | - | - | - | - | - | - | 0.10 | 0.11 | 0.12 | 0.14 | 0.16 | 0.17 | 0.17 | 0.21 | 0.27 | 0.26 | 0.26 | 0.26 | 0.30 |
| XX | - | <0.01 | 0.02 | 0.01 | 0.01 | 0.01 | 0.01 | 0.02 | 0.01 | 0.01 | 0.01 | 0.01 | 0.01 | 0.03 | 0.03 | 0.03 | 0.04 | 0.04 | 0.04 | 0.04 | 0.05 |
| **Belgium** | **1.69** | **1.72** | **1.74** | **1.77** | **1.76** | **1.78** | **1.76** | **1.83** | **1.99** | **2.06** | **2.19** | **2.35** | **2.46** | **2.53** | **2.60** | **2.63** | **2.71** | **2.76** | **2.73** | **2.75** | **2.51** |
| XA | <0.01 | <0.01 | <0.01 | <0.01 | <0.01 | <0.01 | <0.01 | <0.01 | <0.01 | <0.01 | <0.01 | <0.01 | <0.01 | <0.01 | <0.01 | <0.01 | <0.01 | <0.01 | <0.01 | <0.01 | <0.01 |
| XB | <0.01 | <0.01 | <0.01 | <0.01 | <0.01 | <0.01 | - | <0.01 | <0.01 | <0.01 | <0.01 | <0.01 | 0.01 | 0.01 | 0.01 | 0.01 | 0.01 | 0.01 | 0.01 | 0.01 | 0.01 |
| XC | - | - | - | - | - | - | <0.01 | <0.01 | <0.01 | <0.01 | <0.01 | <0.01 | - | - | - | - | - | - | - | - | - |
| XD | - | - | - | - | - | - | - | - | - | - | - | - | - | - | - | - | - | - | - | - | - |
| XE | 1.59 | 1.61 | 1.62 | 1.64 | 1.63 | 1.63 | 1.61 | 1.66 | 1.87 | 1.97 | 2.08 | 2.22 | 2.32 | 2.39 | 2.45 | 2.48 | 2.54 | 2.59 | 2.57 | 2.59 | 2.34 |
| XX | 0.10 | 0.11 | 0.12 | 0.12 | 0.13 | 0.14 | 0.15 | 0.17 | 0.12 | 0.09 | 0.11 | 0.12 | 0.13 | 0.13 | 0.14 | 0.15 | 0.15 | 0.16 | 0.16 | 0.15 | 0.16 |
| **Bulgaria** | - | - | ***0.41*** | ***0.37*** | ***0.35*** | ***0.05*** | ***0.14*** | ***0.12*** | ***0.23*** | **<0.01** | **<0.01** | **<0.01** | **<0.01** | **<0.01** | **<0.01** | **<0.01** | **<0.01** | **<0.01** | **<0.01** | **0.10** | **0.11** |
| XA | - | - | *<0.01* | *<0.01* | *<0.01* | *0.01* | *<0.01* | *-* | *<0.01* | <0.01 | <0.01 | <0.01 | <0.01 | <0.01 | <0.01 | <0.01 | <0.01 | <0.01 | <0.01 | <0.01 | <0.01 |
| XB | - | - | *-* | *-* | *-* | *-* | *-* | *-* | *-* | - | - | - | - | - | - | - | - | <0.01 | <0.01 | <0.01 | 0.01 |
| XC | - | - | *-* | *<0.01* | *<0.01* | *-* | *-* | *-* | *-* | - | - | - | - | - | - | - | - | - | - | - | - |
| XD | - | - | *0.41* | *0.37* | *0.34* | *0.04* | *0.14* | *0.12* | *0.23* | <0.01 | <0.01 | <0.01 | <0.01 | <0.01 | <0.01 | - | <0.01 | - | <0.01 | <0.01 | <0.01 |
| XE | - | - | *-* | *-* | *-* | *-* | *-* | *-* | *-* | - | - | - | - | - | - | - | - | - | - | - | - |
| XX | - | - | *-* | *<0.01* | *<0.01* | *-* | *-* | *<0.01* | *<0.01* | - | <0.01 | <0.01 | <0.01 | <0.01 | <0.01 | <0.01 | <0.01 | - | - | 0.09 | 0.10 |
| **Croatia** | - | - | - | - | **<0.01** | **0.01** | **0.01** | **0.56** | **0.59** | **0.65** | **0.49** | **0.66** | **0.72** | **0.69** | **0.60** | **0.68** | **0.72** | **0.78** | **0.83** | **0.88** | **0.73** |
| XA | - | - | - | - | <0.01 | <0.01 | <0.01 | <0.01 | <0.01 | <0.01 | <0.01 | <0.01 | <0.01 | <0.01 | - | - | - | - | - | - | - |
| XB | - | - | - | - | - | - | - | - | - | - | - | - | - | - | - | - | - | - | - | - | - |
| XC | - | - | - | - | - | - | - | - | - | - | - | - | - | - | - | - | - | - | - | - | - |
| XD | - | - | - | - | <0.01 | - | - | <0.01 | <0.01 | <0.01 | <0.01 | <0.01 | <0.01 | <0.01 | - | - | - | - | - | - | - |
| XE | - | - | - | - | - | 0.01 | 0.01 | 0.56 | 0.59 | 0.65 | 0.49 | 0.66 | 0.72 | 0.69 | 0.60 | 0.68 | 0.72 | 0.78 | 0.83 | 0.87 | 0.68 |
| XX | - | - | - | - | - | - | - | - | - | - | - | - | <0.01 | - | - | - | - | - | - | <0.01 | 0.05 |
| Country, community consumption of other antibacterials (J01X); XA, consumption of glycopeptide antibacterials (J01XA); XB, consumption of polymyxins (J01XB); XC, consumption of steroid antibacterials (J01XC); XD, consumption of imidazole derivatives (J01XD); XE, consumption of nitrofuran derivatives (J01XD); XX, consumption of other antibacterials (J01XX); **-**, no consumption reported; Numbers reported in *italic* are total care data, i.e. community and hospital sector combined; ^a^Data for Ireland do not include nitrofurantoin (J01XE01) consumption; ^b^Data for Romania have a coverage in 2009 limited to 30-40%; ^c^Data for Spain include private prescriptions from 2016 onwards. | | | | | | | | | | | | | | | | | | | | | |
| **Cyprus** | - | - | - | - | - | - | - | - | - | ***0.36*** | ***0.38*** | ***0.39*** | ***0.44*** | ***0.47*** | ***0.45*** | ***0.49*** | ***0.57*** | ***0.57*** | ***0.69*** | ***0.74*** | ***0.78*** |
| XA | - | - | - | - | - | - | - | - | - | *0.03* | *0.03* | *0.03* | *0.04* | *0.04* | *0.04* | *0.04* | *0.05* | *0.05* | *0.06* | *0.06* | *0.08* |
| XB | - | - | - | - | - | - | - | - | - | *<0.01* | *<0.01* | *<0.01* | *<0.01* | *0.01* | *<0.01* | *<0.01* | *0.01* | *0.01* | *0.01* | *0.01* | *0.01* |
| XC | - | - | - | - | - | - | - | - | - | *<0.01* | *<0.01* | *<0.01* | *<0.01* | *<0.01* | *<0.01* | *<0.01* | *-* | *-* | *-* | *-* | *-* |
| XD | - | - | - | - | - | - | - | - | - | *0.08* | *0.10* | *0.11* | *0.09* | *0.09* | *0.09* | *0.09* | *0.14* | *0.13* | *0.19* | *0.19* | *0.20* |
| XE | - | - | - | - | - | - | - | - | - | *0.18* | *0.20* | *0.20* | *0.28* | *0.30* | *0.29* | *0.34* | *0.36* | *0.36* | *0.41* | *0.45* | *0.46* |
| XX | - | - | - | - | - | - | - | - | - | *0.06* | *0.06* | *0.04* | *0.03* | *0.04* | *0.03* | *0.02* | *0.02* | *0.02* | *0.02* | *0.03* | *0.02* |
| **Czechia** |  | **0.85** | **0.85** |  |  |  | **1.16** | **0.30** | **0.01** | **0.71** | **0.35** | **0.74** | **0.87** | **0.76** | **1.03** | **1.15** | **1.34** | **1.48** | **1.12** | - | - |
| XA | - | - | - | - | - | - | - | - | - | - | - | 0.01 | 0.01 | 0.02 | 0.01 | 0.01 | 0.01 | 0.02 | 0.02 | - | - |
| XB | - | <0.01 | <0.01 | - | - | - | <0.01 | <0.01 | - | - | <0.01 | <0.01 | <0.01 | <0.01 | <0.01 | <0.01 | <0.01 | <0.01 | 0.01 | - | - |
| XC | - | - | - | - | - | - | - | - | - | - | - | - | - | - | - | - | - | - | - | - | - |
| XD | - | - | - | - | - | - | 0.13 |  | 0.01 | 0.15 | - | 0.04 | 0.05 | 0.04 | 0.05 | 0.04 | 0.04 | 0.05 | 0.05 | - | - |
| XE | - | 0.85 | 0.85 | - | - | - | 1.03 | 0.30 | <0.01 | 0.56 | 0.35 | 0.69 | 0.80 | 0.70 | 0.95 | 1.09 | 1.28 | 1.41 | 1.04 | - | - |
| XX | - | - | - | - | - | - | - | - | <0.01 | - | - | <0.01 | <0.01 | <0.01 | <0.01 | <0.01 | <0.01 | <0.01 | <0.01 | - | - |
| **Denmark** | **0.84** | **0.82** | **0.79** | **0.78** | **0.74** | **0.76** | **0.76** | **0.74** | **0.75** | **0.76** | **0.51** | **0.51** | **0.53** | **0.81** | **0.80** | **0.78** | **0.76** | **0.67** | **0.73** | **0.73** | **0.58** |
| XA | <0.01 | <0.01 | <0.01 | <0.01 | <0.01 | <0.01 | <0.01 | <0.01 | <0.01 | <0.01 | <0.01 | <0.01 | <0.01 | <0.01 | <0.01 | <0.01 | <0.01 | <0.01 | <0.01 | - | <0.01 |
| XB | 0.01 | 0.01 | 0.01 | 0.01 | 0.01 | 0.01 | 0.01 | 0.01 | 0.01 | 0.02 | 0.02 | 0.02 | 0.02 | 0.02 | 0.03 | 0.02 | 0.02 | 0.02 | 0.02 | 0.02 | 0.02 |
| XC | 0.02 | 0.02 | 0.02 | 0.02 | 0.01 | 0.01 | 0.01 | 0.01 | 0.01 | 0.01 | 0.02 | 0.02 | 0.01 | 0.01 | 0.01 | 0.01 | 0.01 | 0.01 | 0.01 | 0.01 | 0.01 |
| XD | <0.01 | <0.01 | - | <0.01 | - | - | - | <0.01 | <0.01 | <0.01 | <0.01 | <0.01 | <0.01 | <0.01 | <0.01 | <0.01 | <0.01 | <0.01 | <0.01 | - | - |
| XE | 0.35 | 0.36 | 0.36 | 0.38 | 0.39 | 0.41 | 0.41 | 0.43 | 0.45 | 0.46 | 0.47 | 0.47 | 0.49 | 0.51 | 0.50 | 0.50 | 0.49 | 0.48 | 0.45 | 0.43 | 0.26 |
| XX | 0.46 | 0.43 | 0.40 | 0.37 | 0.33 | 0.33 | 0.32 | 0.30 | 0.28 | 0.27 | <0.01 | <0.01 | <0.01 | 0.27 | 0.26 | 0.25 | 0.24 | 0.16 | 0.25 | 0.27 | 0.28 |
| **Estonia** | - | - | - | - | ***1.33*** | **1.12** | **0.97** | **0.40** | **1.00** | **0.47** | **0.48** | **0.51** | **0.48** | **0.71** | **0.73** | **0.76** | **0.75** | **0.75** | **0.77** | **0.77** | **0.54** |
| XA | - | - | - | - | *<0.01* | - | <0.01 | <0.01 | - | - | - | <0.01 | - | - | - | <0.01 | - | - | - | - | - |
| XB | - | - | - | - | *-* | - | - | - | <0.01 | - | <0.01 | <0.01 | <0.01 | <0.01 | <0.01 | <0.01 | <0.01 | <0.01 | <0.01 | <0.01 | <0.01 |
| XC | - | - | - | - | *<0.01* | <0.01 | <0.01 | <0.01 | - | - | <0.01 | <0.01 | <0.01 | <0.01 | <0.01 | <0.01 | - | <0.01 | - | <0.01 | <0.01 |
| XD | - | - | - | - | *0.82* | 0.65 | 0.59 | <0.01 | 0.57 | <0.01 | <0.01 | <0.01 | <0.01 | 0.25 | 0.25 | 0.25 | 0.23 | 0.22 | 0.22 | 0.22 | <0.01 |
| XE | - | - | - | - | *0.51* | 0.47 | 0.38 | 0.39 | 0.43 | 0.47 | 0.48 | 0.50 | 0.48 | 0.45 | 0.47 | 0.51 | 0.52 | 0.53 | 0.54 | 0.55 | 0.54 |
| XX | - | - | - | - | *-* | <0.01 | <0.01 | <0.01 | <0.01 | <0.01 | <0.01 | <0.01 | <0.01 | - | <0.01 | - | <0.01 | <0.01 | <0.01 | - | - |
| Country, community consumption of other antibacterials (J01X); XA, consumption of glycopeptide antibacterials (J01XA); XB, consumption of polymyxins (J01XB); XC, consumption of steroid antibacterials (J01XC); XD, consumption of imidazole derivatives (J01XD); XE, consumption of nitrofuran derivatives (J01XD); XX, consumption of other antibacterials (J01XX); **-**, no consumption reported; Numbers reported in *italic* are total care data, i.e. community and hospital sector combined; ^a^Data for Ireland do not include nitrofurantoin (J01XE01) consumption; ^b^Data for Romania have a coverage in 2009 limited to 30-40%; ^c^Data for Spain include private prescriptions from 2016 onwards. | | | | | | | | | | | | | | | | | | | | | |
| **Finland** | **1.80** | **1.85** | **1.82** | **1.96** | **1.93** | **1.92** | **1.92** | **2.04** | **1.98** | **2.01** | **1.92** | **2.10** | **2.10** | **2.09** | **2.04** | **2.14** | **2.04** | **1.97** | **1.84** | **1.72** | **1.51** |
| XA | <0.01 | <0.01 | <0.01 | <0.01 | <0.01 | <0.01 | <0.01 | <0.01 | <0.01 | <0.01 | <0.01 | <0.01 | <0.01 | <0.01 | <0.01 | <0.01 | <0.01 | <0.01 | <0.01 | <0.01 | <0.01 |
| XB | - | - | - | - | - | - | - | - | - | - | - | - | - | - | - | - | - | - | - | <0.01 | <0.01 |
| XC | 0.01 | 0.01 | 0.01 | 0.01 | 0.01 | 0.01 | 0.01 | 0.01 | 0.01 | 0.01 | 0.01 | 0.01 | 0.01 | 0.01 | <0.01 | <0.01 | <0.01 | <0.01 | <0.01 | <0.01 | <0.01 |
| XD | <0.01 | <0.01 | <0.01 | <0.01 | - | <0.01 | <0.01 | <0.01 | - | <0.01 | <0.01 | <0.01 | <0.01 | <0.01 | <0.01 | <0.01 | <0.01 | <0.01 | <0.01 | <0.01 | - |
| XE | 0.69 | 0.69 | 0.56 | 0.67 | 0.65 | 0.63 | 0.60 | 0.63 | 0.65 | 0.64 | 0.57 | 0.61 | 0.61 | 0.52 | 0.43 | 0.52 | 0.52 | 0.52 | 0.48 | 0.47 | 0.44 |
| XX | 1.10 | 1.15 | 1.25 | 1.28 | 1.28 | 1.29 | 1.31 | 1.40 | 1.32 | 1.37 | 1.34 | 1.48 | 1.48 | 1.56 | 1.61 | 1.61 | 1.52 | 1.45 | 1.36 | 1.26 | 1.07 |
| **France** | **0.40** | **0.40** | **0.44** | **0.46** | **0.49** | **0.82** | **0.88** | **0.80** | **0.59** | **0.53** | **0.53** | **0.54** | **0.56** | **0.57** | **0.51** | **0.46** | **0.45** | **0.41** | **0.46** | **0.45** | **0.51** |
| XA | - | - | - | - | - | - | - | - | - | - | - | - | - | - | - | - | - | - | - | - | - |
| XB | <0.01 | <0.01 | <0.01 | <0.01 | <0.01 | 0.01 | 0.01 | 0.01 | 0.01 | 0.01 | 0.02 | 0.02 | 0.02 | - | 0.01 | 0.01 | 0.01 | 0.01 | 0.02 | 0.02 | 0.01 |
| XC | 0.10 | 0.10 | 0.12 | 0.13 | 0.14 | 0.15 | 0.16 | 0.15 | 0.14 | 0.14 | 0.12 | 0.11 | 0.10 | 0.10 | 0.09 | 0.09 | 0.08 | 0.08 | 0.07 | 0.06 | 0.05 |
| XD | - | - | - | - | - | - | - | - | 0.01 | - | - | - | - | - | - | - | - | - | - | - | <0.01 |
| XE | 0.25 | 0.25 | 0.26 | 0.27 | 0.28 | 0.29 | 0.29 | 0.30 | 0.32 | 0.29 | 0.30 | 0.31 | 0.33 | 0.35 | 0.29 | 0.23 | 0.22 | 0.18 | 0.20 | 0.20 | 0.24 |
| XX | 0.04 | 0.05 | 0.05 | 0.06 | 0.06 | 0.37 | 0.42 | 0.34 | 0.12 | 0.11 | 0.10 | 0.11 | 0.11 | 0.12 | 0.12 | 0.14 | 0.15 | 0.14 | 0.18 | 0.17 | 0.20 |
| **Germany** | **0.29** | **0.28** | **0.29** | **0.27** | **0.29** | **0.29** | **0.32** | **0.33** | **0.36** | **0.35** | **0.37** | **0.38** | **0.40** | **0.43** | **0.48** | **0.52** | **0.53** | **0.55** | **0.52** | **0.51** | **0.48** |
| XA | <0.01 | <0.01 | <0.01 | <0.01 | <0.01 | <0.01 | <0.01 | <0.01 | <0.01 | <0.01 | <0.01 | <0.01 | <0.01 | <0.01 | <0.01 | <0.01 | <0.01 | <0.01 | <0.01 | <0.01 | <0.01 |
| XB | <0.01 | <0.01 | <0.01 | <0.01 | <0.01 | <0.01 | <0.01 | <0.01 | <0.01 | <0.01 | <0.01 | <0.01 | <0.01 | 0.01 | 0.01 | 0.01 | 0.01 | 0.01 | 0.01 | 0.01 | 0.01 |
| XC | <0.01 | <0.01 | <0.01 | <0.01 | <0.01 | <0.01 | - | - | - | - | - | - | - | - | - | - | - | - | - | - | - |
| XD | - | - | - | - | <0.01 | <0.01 | <0.01 | <0.01 | <0.01 | <0.01 | <0.01 | <0.01 | <0.01 | <0.01 | <0.01 | <0.01 | <0.01 | <0.01 | <0.01 | <0.01 | <0.01 |
| XE | 0.22 | 0.21 | 0.22 | 0.22 | 0.23 | 0.23 | 0.27 | 0.28 | 0.31 | 0.31 | 0.32 | 0.33 | 0.34 | 0.36 | 0.41 | 0.43 | 0.44 | 0.45 | 0.42 | 0.40 | 0.37 |
| XX | 0.07 | 0.07 | 0.06 | 0.05 | 0.06 | 0.05 | 0.05 | 0.04 | 0.04 | 0.04 | 0.05 | 0.05 | 0.05 | 0.06 | 0.06 | 0.07 | 0.08 | 0.08 | 0.09 | 0.09 | 0.10 |
| **Greece** | **0.19** | **0.22** | **0.24** | **0.24** | **0.27** | **0.29** | **0.28** | ***0.29*** | ***0.16*** | ***0.60*** | ***0.67*** | ***0.71*** | **0.37** | ***3.00*** | **0.56** | **0.55** | **0.67** | **0.67** | **0.77** | **0.84** | **0.92** |
| XA | <0.01 | <0.01 | 0.01 | 0.01 | 0.01 | 0.01 | 0.01 | *0.08* | *0.08* | *0.09* | *0.11* | *0.11* | 0.02 | *0.10* | 0.02 | 0.02 | 0.02 | 0.02 | 0.03 | 0.03 | 0.04 |
| XB | <0.01 | <0.01 | <0.01 | <0.01 | <0.01 | <0.01 | <0.01 | *-* | *-* | *0.02* | *0.03* | *0.03* | 0.01 | *0.04* | 0.02 | 0.02 | 0.02 | 0.02 | 0.02 | 0.02 | 0.02 |
| XC | <0.01 | 0.02 | 0.03 | 0.03 | 0.04 | 0.05 | 0.05 | *0.06* | *0.06* | *0.04* | *0.06* | *0.06* | 0.04 | *0.04* | 0.02 | 0.02 | 0.02 | 0.02 | 0.02 | 0.09 | 0.02 |
| XD | 0.01 | 0.02 | 0.02 | 0.02 | 0.03 | 0.04 | 0.02 | *0.14* | *-* | *0.14* | *0.14* | *0.14* | 0.03 | *2.33* | 0.02 | 0.02 | 0.03 | 0.03 | 0.05 | 0.07 | 0.09 |
| XE | 0.17 | 0.17 | 0.18 | 0.18 | 0.18 | 0.19 | 0.20 | *-* | *-* | *0.29* | *0.31* | *0.33* | 0.26 | *0.44* | 0.46 | 0.46 | 0.57 | 0.55 | 0.60 | 0.55 | 0.66 |
| XX | 0.01 | 0.01 | 0.01 | <0.01 | <0.01 | <0.01 | <0.01 | *0.01* | *0.01* | *0.02* | *0.03* | *0.04* | 0.01 | *0.06* | 0.01 | 0.01 | 0.01 | 0.03 | 0.05 | 0.07 | 0.08 |
| Country, community consumption of other antibacterials (J01X); XA, consumption of glycopeptide antibacterials (J01XA); XB, consumption of polymyxins (J01XB); XC, consumption of steroid antibacterials (J01XC); XD, consumption of imidazole derivatives (J01XD); XE, consumption of nitrofuran derivatives (J01XD); XX, consumption of other antibacterials (J01XX); **-**, no consumption reported; Numbers reported in *italic* are total care data, i.e. community and hospital sector combined; ^a^Data for Ireland do not include nitrofurantoin (J01XE01) consumption; ^b^Data for Romania have a coverage in 2009 limited to 30-40%; ^c^Data for Spain include private prescriptions from 2016 onwards. | | | | | | | | | | | | | | | | | | | | | |
| **Hungary** | - | **<0.01** | **0.01** | **0.01** | **0.01** | **0.03** | **0.03** | **0.03** | **0.04** | **0.29** | **0.28** | **0.28** | **0.14** | **0.09** | **0.19** | **0.21** | **0.25** | **0.26** | **0.21** | **0.09** | **0.09** |
| XA | - | - | - | - | - | - | - | - | - | - | - | - | - | - | - | <0.01 | <0.01 | <0.01 | <0.01 | <0.01 | <0.01 |
| XB | - | - | - | - | - | - | - | - | - | - | - | <0.01 | <0.01 | <0.01 | <0.01 | <0.01 | <0.01 | <0.01 | <0.01 | <0.01 | <0.01 |
| XC | - | - | - | - | - | - | <0.01 | <0.01 | <0.01 | <0.01 | - | - | - | - | - | - | - | - | - | - | - |
| XD | - | - | - | - | - | - | <0.01 | - | - | - | - | - | - | - | - | <0.01 | <0.01 | <0.01 | <0.01 | <0.01 | <0.01 |
| XE | - | - | - | - | - | - | - | - | - | 0.25 | 0.24 | 0.25 | 0.10 | 0.04 | 0.14 | 0.16 | 0.18 | 0.19 | 0.13 | - | - |
| XX | - | <0.01 | 0.01 | 0.01 | 0.01 | 0.03 | 0.03 | 0.03 | 0.04 | 0.04 | 0.04 | 0.04 | 0.04 | 0.05 | 0.05 | 0.05 | 0.06 | 0.07 | 0.08 | 0.08 | 0.09 |
| **Iceland** | ***0.92*** | ***0.94*** | ***1.00*** | ***0.29*** | ***0.32*** | ***0.34*** | ***0.31*** | ***0.51*** | ***0.66*** | **0.38** | **0.41** | **0.48** | **0.76** | ***1.03*** | ***1.04*** | ***0.93*** | ***1.12*** | **0.89** | **0.98** | **1.20** | **1.29** |
| XA | *0.02* | *0.01* | *0.02* | *0.02* | *0.01* | *0.01* | *0.01* | *0.02* | *0.02* | - | - | - | - | *0.02* | *0.02* | *0.02* | *0.03* | - | <0.01 | <0.01 | <0.01 |
| XB | *-* | *-* | *-* | *-* | *-* | *-* | *-* | *-* | *-* | - | - | - | - | *-* | *-* | *-* | *-* | - | - | - | - |
| XC | *<0.01* | *<0.01* | *<0.01* | *<0.01* | *<0.01* | *-* | *-* | *-* | *-* | - | - | - | 0.31 | *-* | *-* | *-* | *-* | - | - | - | - |
| XD | *0.06* | *0.05* | *0.06* | *0.06* | *0.06* | *0.06* | *0.05* | *0.05* | *0.06* | - | - | - | - | *0.05* | *0.05* | *0.05* | *0.05* | - | <0.01 | <0.01 | <0.01 |
| XE | *0.66* | *0.71* | *0.73* | *-* | *0.04* | *0.04* | *-* | *0.21* | *0.33* | 0.26 | 0.28 | 0.32 | 0.26 | *0.62* | *0.60* | *0.49* | *0.66* | 0.58 | 0.65 | 0.72 | 0.77 |
| XX | *0.19* | *0.17* | *0.18* | *0.21* | *0.21* | *0.23* | *0.24* | *0.23* | *0.26* | 0.11 | 0.13 | 0.16 | 0.19 | *0.34* | *0.37* | *0.36* | *0.38* | 0.31 | 0.33 | 0.47 | 0.52 |
| **Ireland^a^** | - | **0.24** | **0.25** | **0.26** | **0.28** | **0.21** | **0.24** | **0.27** | **0.33** | **0.10** | **0.10** | **0.08** | **0.06** | **0.05** | **0.05** | **0.04** | **0.04** | **0.03** | **0.04** | **0.04** | **0.03** |
| XA | - | <0.01 | <0.01 | <0.01 | <0.01 | <0.01 | <0.01 | <0.01 | <0.01 | <0.01 | <0.01 | <0.01 | <0.01 | <0.01 | <0.01 | <0.01 | <0.01 | <0.01 | <0.01 | <0.01 | <0.01 |
| XB | - | 0.01 | 0.01 | 0.02 | 0.02 | 0.02 | 0.02 | 0.02 | 0.02 | 0.02 | 0.02 | 0.03 | 0.03 | 0.02 | 0.02 | 0.02 | 0.02 | 0.02 | 0.02 | 0.02 | 0.02 |
| XC | - | 0.02 | 0.02 | 0.02 | 0.02 | 0.02 | 0.02 | 0.02 | 0.02 | 0.02 | 0.02 | 0.02 | 0.02 | 0.02 | 0.01 | 0.01 | 0.01 | 0.01 | 0.01 | <0.01 | <0.01 |
| XD | - | <0.01 | <0.01 | <0.01 | <0.01 | <0.01 | <0.01 | <0.01 | <0.01 | <0.01 | - | <0.01 | <0.01 | <0.01 | <0.01 | <0.01 | <0.01 | <0.01 | <0.01 | <0.01 | <0.01 |
| XE | - | 0.21 | 0.22 | 0.22 | 0.25 | 0.16 | 0.19 | 0.21 | 0.27 | 0.04 | 0.05 | 0.02 | 0.01 | <0.01 | <0.01 | <0.01 | <0.01 | <0.01 | <0.01 | <0.01 | <0.01 |
| XX | - | - | - | - | <0.01 | <0.01 | 0.01 | 0.01 | 0.01 | 0.01 | 0.01 | 0.01 | 0.01 | 0.01 | 0.01 | 0.01 | 0.01 | 0.01 | 0.01 | 0.01 | 0.01 |
| **Italy** | - | - | **0.53** | **0.56** | **0.61** | **0.61** | **0.63** | **0.64** | **0.63** | **0.66** | **0.65** | **0.66** | **0.67** | **0.68** | **0.66** | **0.68** | **0.70** | **0.71** | **0.71** | **0.70** | **0.65** |
| XA | - | - | <0.01 | 0.01 | 0.01 | 0.01 | 0.01 | 0.01 | 0.01 | 0.01 | 0.01 | 0.01 | 0.01 | 0.01 | 0.01 | 0.01 | <0.01 | <0.01 | <0.01 | <0.01 | <0.01 |
| XB | - | - | - | - | - | - | - | - | - | - | <0.01 | <0.01 | <0.01 | <0.01 | <0.01 | <0.01 | <0.01 | <0.01 | <0.01 | <0.01 | <0.01 |
| XC | - | - | - | - | - | - | - | - | - | - | - | - | - | - | - | - | - | - | - | - | - |
| XD | - | - | <0.01 | <0.01 | <0.01 | <0.01 | <0.01 | <0.01 | <0.01 | <0.01 | <0.01 | <0.01 | <0.01 | <0.01 | <0.01 | <0.01 | <0.01 | <0.01 | <0.01 | <0.01 | <0.01 |
| XE | - | - | 0.27 | 0.27 | 0.26 | 0.25 | 0.26 | 0.25 | 0.24 | 0.24 | 0.23 | 0.23 | 0.22 | 0.22 | 0.22 | 0.22 | 0.22 | 0.22 | 0.22 | 0.21 | 0.18 |
| XX | - | - | 0.26 | 0.29 | 0.34 | 0.35 | 0.37 | 0.39 | 0.39 | 0.42 | 0.41 | 0.43 | 0.44 | 0.45 | 0.44 | 0.45 | 0.48 | 0.49 | 0.49 | 0.49 | 0.46 |
| Country, community consumption of other antibacterials (J01X); XA, consumption of glycopeptide antibacterials (J01XA); XB, consumption of polymyxins (J01XB); XC, consumption of steroid antibacterials (J01XC); XD, consumption of imidazole derivatives (J01XD); XE, consumption of nitrofuran derivatives (J01XD); XX, consumption of other antibacterials (J01XX); **-**, no consumption reported; Numbers reported in *italic* are total care data, i.e. community and hospital sector combined; ^a^Data for Ireland do not include nitrofurantoin (J01XE01) consumption; ^b^Data for Romania have a coverage in 2009 limited to 30-40%; ^c^Data for Spain include private prescriptions from 2016 onwards. | | | | | | | | | | | | | | | | | | | | | |
| **Latvia** | - | - | - | - | - | **0.61** | - | **0.61** | **0.56** | **0.49** | **0.49** | **0.33** | **0.27** | **0.25** | **0.26** | **0.26** | **0.29** | **0.33** | **0.33** | **0.30** | **0.71** |
| XA | - | - | - | - | - | 0.01 | - | <0.01 | <0.01 | <0.01 | <0.01 | <0.01 | <0.01 | <0.01 | <0.01 | <0.01 | <0.01 | <0.01 | <0.01 | <0.01 | <0.01 |
| XB | - | - | - | - | - | - | - | - | - | - | - | - | - | - | - | <0.01 | <0.01 | <0.01 | <0.01 | <0.01 | <0.01 |
| XC | - | - | - | - | - | <0.01 | - | - | - | - | - | - | - | - | - | - | - | - | - | - | - |
| XD | - | - | - | - | - | 0.17 | - | 0.21 | 0.20 | 0.14 | 0.13 | <0.01 | <0.01 | <0.01 | 0.02 | 0.01 | 0.01 | 0.01 | 0.01 | <0.01 | <0.01 |
| XE | - | - | - | - | - | 0.34 | - | 0.32 | 0.28 | 0.27 | 0.28 | 0.26 | 0.22 | 0.20 | 0.19 | 0.21 | 0.24 | 0.28 | 0.28 | 0.26 | 0.68 |
| XX | - | - | - | - | - | 0.09 | - | 0.07 | 0.08 | 0.08 | 0.07 | 0.07 | 0.06 | 0.05 | 0.04 | 0.04 | 0.04 | 0.03 | 0.03 | 0.03 | 0.03 |
| **Lithuania** | - | - | - | - | - | - | - | - | - | ***1.44*** | ***2.19*** | ***2.52*** | ***2.73*** | ***1.98*** | ***1.97*** | **1.33** | **1.37** | **1.44** | **1.42** | **1.43** | **1.17** |
| XA | - | - | - | - | - | - | - | - | - | *<0.01* | *0.01* | *0.01* | *0.01* | *0.01* | *0.01* | <0.01 | <0.01 | <0.01 | <0.01 | <0.01 | <0.01 |
| XB | - | - | - | - | - | - | - | - | - | *-* | *-* | *-* | *-* | *-* | *-* | - | - | - | - | - | - |
| XC | - | - | - | - | - | - | - | - | - | *-* | *-* | *-* | *-* | *-* | *-* | - | - | - | - | - | - |
| XD | - | - | - | - | - | - | - | - | - | *0.39* | *0.63* | *0.94* | *1.44* | *0.77* | *0.73* | 0.02 | 0.01 | <0.01 | <0.01 | <0.01 | <0.01 |
| XE | - | - | - | - | - | - | - | - | - | *0.97* | *1.44* | *1.46* | *1.20* | *1.16* | *1.18* | 1.27 | 1.33 | 1.41 | 1.40 | 1.41 | 1.14 |
| XX | - | - | - | - | - | - | - | - | - | *0.08* | *0.11* | *0.11* | *0.09* | *0.05* | *0.05* | 0.03 | 0.03 | 0.03 | 0.02 | 0.02 | 0.03 |
| **Luxembourg** | **1.31** | **1.31** | **1.37** | **1.37** | **1.27** | **1.46** | **1.15** | **1.47** | **1.29** | **0.93** | **1.04** | **1.10** | **1.23** | **1.22** | **1.41** | **1.31** | **1.31** | **1.28** | **1.27** | **1.23** | **1.78** |
| XA | 0.01 | <0.01 | 0.01 | <0.01 | <0.01 | 0.01 | 0.01 | <0.01 | 0.01 | <0.01 | <0.01 | <0.01 | <0.01 | <0.01 | <0.01 | <0.01 | <0.01 | <0.01 | <0.01 | - | - |
| XB | <0.01 | <0.01 | <0.01 | <0.01 | <0.01 | <0.01 | <0.01 | <0.01 | <0.01 | <0.01 | <0.01 | <0.01 | <0.01 | <0.01 | <0.01 | <0.01 | <0.01 | - | <0.01 | <0.01 | - |
| XC | 0.01 | 0.01 | 0.01 | 0.01 | <0.01 | <0.01 | 0.01 | <0.01 | <0.01 | <0.01 | <0.01 | <0.01 | <0.01 | <0.01 | - | - | - | - | - | - | - |
| XD | 0.13 | 0.13 | 0.11 | 0.11 | 0.12 | 0.12 | 0.12 | 0.14 | 0.14 | - | - | <0.01 | - | - | - | - | - | - | - | - | - |
| XE | 0.92 | 0.92 | 0.98 | 0.97 | 0.87 | 0.99 | 0.93 | 0.99 | 1.00 | 0.89 | 0.99 | 1.04 | 1.16 | 1.15 | 1.34 | 1.23 | 1.22 | 1.19 | 1.16 | 1.12 | 0.47 |
| XX | 0.24 | 0.25 | 0.26 | 0.27 | 0.27 | 0.34 | 0.09 | 0.33 | 0.14 | 0.04 | 0.05 | 0.06 | 0.06 | 0.07 | 0.07 | 0.07 | 0.09 | 0.10 | 0.10 | 0.11 | 1.30 |
| **Malta** | - | - | - | - | - | - | - | - | - | - | **0.14** | **0.12** | **0.17** | **0.24** | **0.41** | **0.34** | **0.40** | **0.48** | **0.99** | **0.31** | **0.40** |
| XA | - | - | - | - | - | - | - | - | - | - | - | - | - | - | - | - | - | <0.01 | - | - | <0.01 |
| XB | - | - | - | - | - | - | - | - | - | - | - | <0.01 | <0.01 | <0.01 | 0.01 | - | <0.01 | - | 0.02 | 0.01 | 0.01 |
| XC | - | - | - | - | - | - | - | - | - | - | 0.02 | <0.01 | <0.01 | <0.01 | <0.01 | <0.01 | - | <0.01 | <0.01 | <0.01 | <0.01 |
| XD | - | - | - | - | - | - | - | - | - | - | 0.04 | <0.01 | <0.01 | <0.01 | 0.11 | 0.04 | 0.04 | 0.04 | <0.01 | <0.01 | <0.01 |
| XE | - | - | - | - | - | - | - | - | - | - | 0.08 | 0.11 | 0.17 | 0.23 | 0.28 | 0.31 | 0.36 | 0.44 | 0.96 | 0.29 | 0.37 |
| XX | - | - | - | - | - | - | - | - | - | - | - | - | - | - | - | - | - | - | - | <0.01 | 0.01 |
| Country, community consumption of other antibacterials (J01X); XA, consumption of glycopeptide antibacterials (J01XA); XB, consumption of polymyxins (J01XB); XC, consumption of steroid antibacterials (J01XC); XD, consumption of imidazole derivatives (J01XD); XE, consumption of nitrofuran derivatives (J01XD); XX, consumption of other antibacterials (J01XX); **-**, no consumption reported; Numbers reported in *italic* are total care data, i.e. community and hospital sector combined; ^a^Data for Ireland do not include nitrofurantoin (J01XE01) consumption; ^b^Data for Romania have a coverage in 2009 limited to 30-40%; ^c^Data for Spain include private prescriptions from 2016 onwards. | | | | | | | | | | | | | | | | | | | | | |
| **Netherlands** | **0.67** | **0.66** | **0.71** | **0.75** | **0.78** | **0.79** | **0.81** | **0.84** | **0.93** | **1.05** | **1.10** | **1.14** | **1.20** | **1.26** | **1.35** | **1.43** | **1.43** | **1.47** | **1.47** | **1.47** | **1.45** |
| XA | <0.01 | <0.01 | <0.01 | <0.01 | <0.01 | <0.01 | <0.01 | <0.01 | <0.01 | <0.01 | <0.01 | <0.01 | <0.01 | <0.01 | <0.01 | <0.01 | <0.01 | <0.01 | <0.01 | <0.01 | <0.01 |
| XB | <0.01 | 0.01 | 0.01 | 0.01 | 0.01 | 0.01 | 0.01 | 0.01 | 0.01 | 0.01 | <0.01 | <0.01 | <0.01 | <0.01 | <0.01 | <0.01 | <0.01 | 0.01 | 0.01 | 0.01 | 0.03 |
| XC | <0.01 | <0.01 | <0.01 | <0.01 | <0.01 | <0.01 | <0.01 | <0.01 | <0.01 | <0.01 | <0.01 | <0.01 | <0.01 | <0.01 | <0.01 | <0.01 | <0.01 | <0.01 | <0.01 | <0.01 | <0.01 |
| XD | <0.01 | <0.01 | <0.01 | <0.01 | <0.01 | <0.01 | <0.01 | <0.01 | <0.01 | <0.01 | <0.01 | <0.01 | <0.01 | <0.01 | <0.01 | <0.01 | <0.01 | <0.01 | <0.01 | <0.01 | <0.01 |
| XE | 0.59 | 0.59 | 0.64 | 0.68 | 0.71 | 0.74 | 0.78 | 0.80 | 0.90 | 1.01 | 1.06 | 1.13 | 1.19 | 1.22 | 1.31 | 1.38 | 1.38 | 1.40 | 1.40 | 1.40 | 1.36 |
| XX | 0.07 | 0.07 | 0.07 | 0.06 | 0.06 | 0.04 | 0.03 | 0.02 | 0.03 | 0.03 | 0.03 | 0.01 | 0.01 | 0.04 | 0.04 | 0.05 | 0.05 | 0.06 | 0.06 | 0.06 | 0.07 |
| **Norway** | - | **2.09** | - | - | **2.43** | **2.40** | **2.46** | **2.68** | **2.93** | **2.34** | **2.51** | **2.67** | **2.85** | **3.06** | **3.16** | **3.29** | **3.40** | **3.58** | **3.74** | **3.87** | **3.94** |
| XA | - | - | - | - | - | - | - | - | - | <0.01 | <0.01 | <0.01 | <0.01 | <0.01 | <0.01 | <0.01 | <0.01 | <0.01 | <0.01 | <0.01 | <0.01 |
| XB | - | - | - | - | - | - | - | - | - | <0.01 | <0.01 | <0.01 | <0.01 | <0.01 | <0.01 | <0.01 | <0.01 | 0.01 | <0.01 | 0.01 | 0.01 |
| XC | - | <0.01 | - | - | 0.01 | 0.01 | 0.01 | 0.01 | 0.01 | <0.01 | <0.01 | <0.01 | <0.01 | <0.01 | <0.01 | <0.01 | <0.01 | <0.01 | <0.01 | <0.01 | <0.01 |
| XD | - | - | - | - | - | - | - | - | - | <0.01 | <0.01 | <0.01 | <0.01 | <0.01 | <0.01 | <0.01 | <0.01 | <0.01 | <0.01 | <0.01 | <0.01 |
| XE | - | 0.37 | - | - | 0.35 | 0.35 | 0.34 | 0.36 | 0.35 | 0.31 | 0.31 | 0.30 | 0.31 | 0.32 | 0.33 | 0.32 | 0.31 | 0.30 | 0.29 | 0.28 | 0.27 |
| XX | - | 1.72 | - | - | 2.06 | 2.04 | 2.11 | 2.32 | 2.57 | 2.03 | 2.20 | 2.36 | 2.54 | 2.73 | 2.82 | 2.96 | 3.08 | 3.27 | 3.44 | 3.58 | 3.66 |
| **Poland** | - | **0.13** | **0.39** | **0.66** | **0.65** | **0.52** | - | **1.95** | **2.01** | - | **<0.01** | **<0.01** | **1.44** | **2.16** | **0.40** | **2.87** | **3.45** | **3.55** | **3.88** | **2.62** | **4.32** |
| XA | - | <0.01 | 0.01 | <0.01 | <0.01 | <0.01 | - | - | - | - | - | - | - | - | - | <0.01 | <0.01 | <0.01 | <0.01 | <0.01 | <0.01 |
| XB | - | <0.01 | <0.01 | <0.01 | <0.01 | <0.01 | - | - | - | - | <0.01 | <0.01 | <0.01 | <0.01 | <0.01 | <0.01 | <0.01 | <0.01 | <0.01 | <0.01 | <0.01 |
| XC | - | - | - | - | - | - | - | - | - | - | - | - | - | - | - | - | - | - | - | - | - |
| XD | - | 0.01 | 0.03 | 0.04 | 0.03 | 0.03 | - | 0.02 | 0.02 | - | - | - | 0.01 | 0.01 | 0.02 | - | 0.22 | <0.01 | <0.01 | 0.03 | <0.01 |
| XE | - | 0.11 | 0.34 | 0.62 | 0.62 | 0.48 | - | 1.93 | 1.99 | - | - | - | 1.42 | 2.14 | 0.38 | 2.87 | 3.21 | 3.53 | 3.85 | 2.57 | 4.26 |
| XX | - | <0.01 | <0.01 | <0.01 | <0.01 | <0.01 | - | <0.01 | <0.01 | - | <0.01 | <0.01 | <0.01 | <0.01 | <0.01 | - | 0.02 | 0.02 | 0.02 | 0.03 | 0.05 |
| **Portugal** | **0.53** | **0.52** | **0.52** | **0.13** | **0.07** | **0.89** | **0.10** | **0.74** | **0.11** | **0.14** | - | **0.85** | **0.95** | **0.99** | **1.37** | **1.44** | **1.00** | **1.10** | **1.13** | **1.15** | **1.13** |
| XA | - | - | - | - | - | - | - | - | - | - | - | - | - | - | - | - | - | - | - | - | - |
| XB | - | - | - | - | - | - | - | - | - | - | - | - | - | - | - | - | - | - | - | - | - |
| XC | <0.01 | - | - | - | 0.01 | 0.03 | 0.03 | 0.03 | 0.03 | 0.06 | - | 0.06 | 0.06 | 0.06 | 0.05 | 0.04 | 0.03 | 0.03 | 0.02 | 0.02 | 0.02 |
| XD | 0.04 | 0.04 | 0.04 | 0.03 | 0.03 | 0.02 | 0.02 | - | - | - | - | - | - | - | - | - | - | - | - | - | - |
| XE | 0.48 | 0.47 | 0.47 | 0.08 | <0.01 | 0.81 | - | 0.65 | - | - | - | 0.69 | 0.78 | 0.81 | 1.16 | 1.21 | 0.82 | 0.90 | 0.93 | 0.93 | 0.92 |
| XX | 0.01 | 0.01 | 0.02 | 0.02 | 0.03 | 0.04 | 0.05 | 0.06 | 0.07 | 0.08 | - | 0.09 | 0.11 | 0.12 | 0.16 | 0.18 | 0.15 | 0.17 | 0.18 | 0.20 | 0.19 |
| Country, community consumption of other antibacterials (J01X); XA, consumption of glycopeptide antibacterials (J01XA); XB, consumption of polymyxins (J01XB); XC, consumption of steroid antibacterials (J01XC); XD, consumption of imidazole derivatives (J01XD); XE, consumption of nitrofuran derivatives (J01XD); XX, consumption of other antibacterials (J01XX); **-**, no consumption reported; Numbers reported in *italic* are total care data, i.e. community and hospital sector combined; ^a^Data for Ireland do not include nitrofurantoin (J01XE01) consumption; ^b^Data for Romania have a coverage in 2009 limited to 30-40%; ^c^Data for Spain include private prescriptions from 2016 onwards. | | | | | | | | | | | | | | | | | | | | | |
| **Romania^a^** | - | - | - | - | - | - | - | - | - | - | - | - | ***0.02*** | - | ***0.11*** | ***0.12*** | ***0.14*** | ***0.16*** | ***0.19*** | ***0.24*** | ***0.24*** |
| XA | - | - | - | - | - | - | - | - | - | - | - | - | *-* | - | *0.01* | *0.01* | *0.02* | *0.01* | *0.03* | *0.03* | *0.04* |
| XB | - | - | - | - | - | - | - | - | - | - | - | - | *<0.01* | - | *0.01* | *0.01* | *0.01* | *0.01* | *0.01* | *0.01* | *0.01* |
| XC | - | - | - | - | - | - | - | - | - | - | - | - | *-* | - | *-* | *-* | *-* | *-* | *-* | *-* | *-* |
| XD | - | - | - | - | - | - | - | - | - | - | - | - | *0.02* | - | *0.03* | *0.03* | *0.03* | *0.03* | *0.03* | *0.06* | *0.06* |
| XE | - | - | - | - | - | - | - | - | - | - | - | - | *-* | - | *0.05* | *0.06* | *0.07* | *0.08* | *0.09* | *0.10* | *0.10* |
| XX | - | - | - | - | - | - | - | - | - | - | - | - | *-* | - | *0.01* | *0.01* | *0.01* | *0.02* | *0.03* | *0.04* | *0.04* |
| **Slovakia** | - | - | **<0.01** | **0.74** | **0.79** | **0.81** | **0.72** | **0.01** | **0.01** | **0.02** | **0.02** | **0.02** | **0.03** | - | ***0.39*** | **0.03** | **0.05** | **0.06** | **0.06** | **0.08** | - |
| XA | - | - | <0.01 | <0.01 | 0.01 | 0.01 | 0.01 | <0.01 | <0.01 | <0.01 | <0.01 | <0.01 | <0.01 | - | *0.01* | <0.01 | <0.01 | <0.01 | <0.01 | <0.01 | - |
| XB | - | - | <0.01 | <0.01 | <0.01 | <0.01 | <0.01 | <0.01 | <0.01 | <0.01 | <0.01 | <0.01 | <0.01 | - | *0.01* | <0.01 | <0.01 | <0.01 | 0.01 | 0.02 | - |
| XC | - | - | - | - | - | - | - | - | - | - | - | - | - | - | *-* | - | - | - | - | - | - |
| XD | - | - | <0.01 | 0.02 | 0.02 | 0.02 | 0.02 | <0.01 | <0.01 | <0.01 | <0.01 | <0.01 | <0.01 | - | *0.34* | <0.01 | <0.01 | 0.01 | 0.02 | 0.02 | - |
| XE | - | - | - | 0.72 | 0.77 | 0.78 | 0.69 | - | - | - | - | - | - | - | *-* | - | - | - | - | - | - |
| XX | - | - | <0.01 | - | <0.01 | <0.01 | <0.01 | <0.01 | <0.01 | 0.01 | 0.02 | 0.02 | 0.02 | - | *0.03* | 0.03 | 0.05 | 0.05 | 0.04 | 0.04 | - |
| **Slovenia** | **<0.01** | **<0.01** | **<0.01** | **<0.01** | **<0.01** | **<0.01** | **<0.01** | **<0.01** | **<0.01** | **<0.01** | **<0.01** | **<0.01** | **<0.01** | **0.01** | **0.06** | **0.11** | **0.19** | **0.26** | **0.36** | **0.36** | **0.40** |
| XA | - | - | - | - | - | - | - | - | - | - | - | - | - | - | - | <0.01 | - | - | - | - | - |
| XB | - | - | - | - | - | - | - | - | - | - | - | - | - | - | - | - | <0.01 | <0.01 | <0.01 | <0.01 | <0.01 |
| XC | - | - | - | - | <0.01 | <0.01 | <0.01 | <0.01 | <0.01 | - | - | - | - | - | - | - | - | - | - | - | - |
| XD | - | - | - | - | - | - | - | - | - | - | - | - | - | - | - | - | - | - | - | - | - |
| XE | - | - | - | - | - | - | - | - | - | - | - | - | - | 0.01 | 0.06 | 0.11 | 0.18 | 0.25 | 0.36 | 0.36 | 0.39 |
| XX | <0.01 | <0.01 | <0.01 | <0.01 | - | - | - | - | <0.01 | <0.01 | <0.01 | <0.01 | <0.01 | <0.01 | <0.01 | <0.01 | <0.01 | <0.01 | <0.01 | <0.01 | <0.01 |
| **Spain^b^** | **0.11** | **0.12** | **0.13** | **0.14** | **0.16** | **0.17** | **0.20** | **0.23** | **0.25** | **0.27** | **0.29** | **0.32** | **0.34** | **0.39** | **0.41** | **0.41** | **0.44** | **0.46** | **0.47** | **0.51** | **0.48** |
| XA | <0.01 | <0.01 | <0.01 | <0.01 | <0.01 | <0.01 | <0.01 | <0.01 | <0.01 | <0.01 | <0.01 | <0.01 | <0.01 | <0.01 | <0.01 | <0.01 | <0.01 | <0.01 | <0.01 | <0.01 | <0.01 |
| XB | <0.01 | <0.01 | <0.01 | <0.01 | <0.01 | - | - | - | - | - | - | - | - | - | - | - | - | - | - | - | - |
| XC | - | <0.01 | 0.01 | 0.01 | 0.01 | 0.01 | 0.02 | 0.01 | 0.01 | 0.01 | 0.01 | 0.01 | 0.01 | 0.01 | 0.01 | 0.01 | 0.01 | <0.01 | <0.01 | <0.01 | <0.01 |
| XD | - | - | - | - | - | - | - | - | - | <0.01 | <0.01 | <0.01 | <0.01 | <0.01 | <0.01 | <0.01 | - | - | - | - | - |
| XE | 0.06 | 0.06 | 0.06 | 0.06 | 0.06 | 0.07 | 0.07 | 0.07 | 0.08 | 0.08 | 0.08 | 0.09 | 0.09 | 0.11 | 0.11 | 0.12 | 0.13 | 0.13 | 0.14 | 0.13 | 0.09 |
| XX | 0.05 | 0.06 | 0.06 | 0.07 | 0.08 | 0.09 | 0.12 | 0.14 | 0.16 | 0.18 | 0.20 | 0.22 | 0.24 | 0.27 | 0.29 | 0.29 | 0.31 | 0.33 | 0.33 | 0.37 | 0.39 |
| Country, community consumption of other antibacterials (J01X); XA, consumption of glycopeptide antibacterials (J01XA); XB, consumption of polymyxins (J01XB); XC, consumption of steroid antibacterials (J01XC); XD, consumption of imidazole derivatives (J01XD); XE, consumption of nitrofuran derivatives (J01XD); XX, consumption of other antibacterials (J01XX); **-**, no consumption reported; Numbers reported in *italic* are total care data, i.e. community and hospital sector combined; ^a^Data for Ireland do not include nitrofurantoin (J01XE01) consumption; ^b^Data for Romania have a coverage in 2009 limited to 30-40%; ^c^Data for Spain include private prescriptions from 2016 onwards. | | | | | | | | | | | | | | | | | | | | | |
| **Sweden** | **1.12** | **1.16** | **1.64** | **1.73** | **1.76** | **1.82** | **1.87** | **2.01** | **2.06** | **2.07** | **2.06** | **1.87** | **1.74** | **1.65** | **1.62** | **1.62** | **1.61** | **1.60** | **1.59** | **1.56** | **1.52** |
| XA | <0.01 | <0.01 | <0.01 | <0.01 | <0.01 | <0.01 | <0.01 | <0.01 | <0.01 | <0.01 | <0.01 | <0.01 | <0.01 | <0.01 | <0.01 | <0.01 | <0.01 | <0.01 | <0.01 | <0.01 | <0.01 |
| XB | - | - | - | - | - | - | - | - | <0.01 | - | <0.01 | <0.01 | <0.01 | <0.01 | <0.01 | <0.01 | <0.01 | <0.01 | <0.01 | <0.01 | 0.01 |
| XC | 0.02 | 0.03 | 0.03 | 0.03 | 0.03 | 0.02 | 0.02 | 0.02 | 0.03 | 0.02 | 0.02 | 0.02 | 0.02 | 0.02 | 0.02 | 0.01 | 0.01 | 0.01 | 0.01 | 0.01 | 0.01 |
| XD | <0.01 | <0.01 | <0.01 | <0.01 | <0.01 | <0.01 | <0.01 | - | <0.01 | <0.01 | <0.01 | <0.01 | <0.01 | <0.01 | <0.01 | <0.01 | <0.01 | <0.01 | <0.01 | <0.01 | <0.01 |
| XE | 0.10 | 0.12 | 0.13 | 0.14 | 0.16 | 0.17 | 0.18 | 0.20 | 0.23 | 0.24 | 0.29 | 0.28 | 0.32 | 0.32 | 0.34 | 0.35 | 0.37 | 0.38 | 0.38 | 0.39 | 0.40 |
| XX | 0.99 | 1.01 | 1.48 | 1.56 | 1.58 | 1.62 | 1.67 | 1.78 | 1.80 | 1.81 | 1.74 | 1.56 | 1.40 | 1.31 | 1.26 | 1.26 | 1.23 | 1.20 | 1.19 | 1.15 | 1.10 |
| **UK** | **0.20** | **0.19** | **0.19** | **0.19** | **0.20** | **0.21** | **0.22** | **0.24** | **0.26** | **0.30** | **0.34** | **0.41** | **0.49** | **0.91** | **0.75** | **0.85** | **0.96** | **0.96** | **1.01** | **1.05** | **1.21** |
| XA | <0.01 | <0.01 | <0.01 | <0.01 | <0.01 | <0.01 | <0.01 | <0.01 | <0.01 | <0.01 | <0.01 | <0.01 | <0.01 | <0.01 | <0.01 | <0.01 | 0.01 | 0.01 | 0.01 | 0.01 | 0.01 |
| XB | 0.01 | 0.01 | 0.01 | 0.01 | 0.01 | 0.01 | 0.01 | 0.01 | 0.01 | 0.03 | 0.04 | 0.04 | 0.02 | 0.04 | 0.03 | 0.03 | 0.03 | 0.03 | 0.03 | 0.03 | 0.05 |
| XC | 0.01 | 0.01 | 0.01 | 0.01 | 0.01 | 0.01 | 0.01 | 0.01 | 0.01 | 0.01 | 0.01 | 0.01 | 0.01 | 0.01 | 0.01 | 0.01 | 0.01 | 0.01 | 0.01 | 0.01 | 0.01 |
| XD | <0.01 | <0.01 | <0.01 | <0.01 | <0.01 | - | - | - | - | - | - | - | - | 0.30 | <0.01 | - | 0.03 | <0.01 | <0.01 | <0.01 | <0.01 |
| XE | 0.17 | 0.16 | 0.15 | 0.15 | 0.16 | 0.17 | 0.18 | 0.20 | 0.22 | 0.25 | 0.28 | 0.35 | 0.45 | 0.54 | 0.69 | 0.79 | 0.86 | 0.87 | 0.90 | 0.94 | 1.05 |
| XX | 0.02 | 0.02 | 0.02 | 0.02 | 0.01 | 0.01 | 0.01 | 0.01 | 0.01 | 0.01 | 0.01 | 0.01 | 0.01 | 0.02 | 0.02 | 0.02 | 0.03 | 0.04 | 0.05 | 0.07 | 0.09 |

Country, community consumption of other antibacterials (J01X); XA, consumption of glycopeptide antibacterials (J01XA); XB, consumption of polymyxins (J01XB); XC, consumption of steroid antibacterials (J01XC); XD, consumption of imidazole derivatives (J01XD); XE, consumption of nitrofuran derivatives (J01XD); XX, consumption of other antibacterials (J01XX); **-**, no consumption reported; Numbers reported in *italic* are total care data, i.e. community and hospital sector combined; ^a^Data for Ireland do not include nitrofurantoin (J01XE01) consumption; ^b^Data for Romania have a coverage in 2009 limited to 30-40%; ^c^Data for Spain include private prescriptions from 2016 onwards.

**Table S6. Consumption of other antibacterials (ATC J01X) in the community, expressed in packages per 1000 inhabitants per day, 23 EU/EEA countries, 2006-2017.**

| **Country** | **2006** | **2007** | **2008** | **2009** | **2010** | **2011** | **2012** | **2013** | **2014** | **2015** | **2016** | **2017** |  |
| --- | --- | --- | --- | --- | --- | --- | --- | --- | --- | --- | --- | --- | --- |
| **Austria** | - | **0.03** | **0.03** | **0.04** | **0.04** | **0.04** | **0.05** | **0.06** | **0.06** | **0.06** | **0.06** | **0.07** |  |
| XA | - | <0.01 | <0.01 | <0.01 | <0.01 | <0.01 | <0.01 | <0.01 | <0.01 | <0.01 | <0.01 | <0.01 |  |
| XB | - | <0.01 | <0.01 | <0.01 | <0.01 | <0.01 | <0.01 | <0.01 | <0.01 | <0.01 | <0.01 | <0.01 |  |
| XC | - | 0.01 | 0.01 | 0.01 | 0.01 | 0.01 | 0.01 | 0.01 | 0.01 | 0.01 | 0.01 | 0.01 |  |
| XD | - | <0.01 | <0.01 | - | - | <0.01 | - | <0.01 | <0.01 | <0.01 | - | - |  |
| XE | - | 0.01 | 0.01 | 0.01 | 0.01 | 0.01 | 0.01 | 0.02 | 0.02 | 0.02 | 0.02 | 0.02 |  |
| XX | - | 0.02 | 0.02 | 0.02 | 0.02 | 0.02 | 0.02 | 0.03 | 0.03 | 0.03 | 0.03 | 0.04 |  |
| **Belgium^a^** | - | **0.19** | **0.21** | **0.22** | **0.23** | **0.24** | **0.24** | **0.25** | **0.26** | **0.26** | **0.56** | **0.52** |  |
| XA | - | <0.01 | <0.01 | <0.01 | <0.01 | <0.01 | <0.01 | <0.01 | - | <0.01 | <0.01 | <0.01 |  |
| XB | - | <0.01 | <0.01 | <0.01 | <0.01 | <0.01 | <0.01 | <0.01 | <0.01 | <0.01 | <0.01 | <0.01 |  |
| XC | - | - | - | - | - | - | - | - | - | - | - | - |  |
| XD | - | - | - | - | - | - | - | - | - | - | 0.01 | 0.01 |  |
| XE | - | 0.08 | 0.08 | 0.09 | 0.09 | 0.09 | 0.09 | 0.10 | 0.10 | 0.10 | 0.40 | 0.36 |  |
| XX | - | 0.11 | 0.12 | 0.13 | 0.13 | 0.14 | 0.15 | 0.15 | 0.16 | 0.16 | 0.15 | 0.16 |  |
| **Bulgaria** | **0.01** | **0.01** | **0.01** | **0.01** | **0.01** | **0.01** | **<0.01** | **0.01** | **<0.01** | **0.01** | **0.05** | **0.06** |  |
| XA | <0.01 | <0.01 | <0.01 | <0.01 | <0.01 | <0.01 | <0.01 | <0.01 | <0.01 | <0.01 | <0.01 | <0.01 |  |
| XB | - | - | - | - | - | - | - | - | <0.01 | <0.01 | <0.01 | <0.01 |  |
| XC | - | - | - | - | - | - | - | - | - | - | - | - |  |
| XD | 0.01 | 0.01 | 0.01 | 0.01 | 0.01 | 0.01 | - | 0.01 | - | 0.01 | 0.01 | 0.01 |  |
| XE | - | - | - | - | - | - | - | - | - | - | - | - |  |
| XX | - | <0.01 | <0.01 | <0.01 | <0.01 | <0.01 | <0.01 | <0.01 | - | - | 0.04 | 0.05 |  |
| **Croatia** | - | **0.07** | **0.09** | **0.10** | **0.09** | **0.08** | **0.09** | **0.10** | **0.10** | **0.11** | **0.12** | **0.12** |  |
| XA | - | <0.01 | <0.01 | <0.01 | <0.01 | - | - | - | - | - | - | - |  |
| XB | - | - | - | - | - | - | - | - | - | - | - | - |  |
| XC | - | - | - | - | - | - | - | - | - | - | - | - |  |
| XD | - | <0.01 | <0.01 | <0.01 | <0.01 | <0.01 | - | - | - | - | - | - |  |
| XE | - | 0.06 | 0.09 | 0.10 | 0.09 | 0.08 | 0.09 | 0.10 | 0.10 | 0.11 | 0.12 | 0.09 |  |
| XX | - | - | - | - | - | - | - | - | - | - | <0.01 | 0.03 |  |
| Country, community consumption of other antibacterials (J01X); XA, consumption of glycopeptide antibacterials (J01XA); XB, consumption of polymyxins (J01XB); XC, consumption of steroid antibacterials (J01XC); XD, consumption of imidazole derivatives (J01XD); XE, consumption of nitrofuran derivatives (J01XE); XX, consumption of other antibacterials (J01XX); **-**, no consumption reported; Numbers reported in *italic* are total care data, i.e. i.e. community and hospital sector combined; ^a^Data for Belgium are slightly overestimated from 2016 onwards (nursing homes counting units versus packages before 2016); ^b^Data for Ireland do not include nitrofurantoin (J01XE01) consumption; ^c^Data for the Netherlands are based on average package size; ^d^Data for Spain include private prescriptions from 2016 onwards. | | | | | | | | | | | | | |
| **Czechia** | - | **0.07** | - | - | **0.19** | **0.21** | **0.20** | **0.19** | **0.22** | **0.21** | - | - |  |
| XA | - | - | - | - | 0.04 | 0.04 | 0.04 | 0.04 | 0.04 | 0.04 | - | - |  |
| XB | - | <0.01 | - | - | <0.01 | <0.01 | <0.01 | <0.01 | <0.01 | <0.01 | - | - |  |
| XC | - | - | - | - | - | - | - | - | - | - | - | - |  |
| XD | - | 0.04 | - | - | 0.12 | 0.12 | 0.11 | 0.10 | 0.12 | 0.12 | - | - |  |
| XE | - | 0.03 | - | - | 0.03 | 0.04 | 0.04 | 0.05 | 0.06 | 0.04 | - | - |  |
| XX | - | - | - | - | <0.01 | <0.01 | <0.01 | <0.01 | <0.01 | <0.01 | - | - |  |
| **Denmark** | - | **0.04** | **0.04** | **0.05** | **0.04** | **0.05** | **0.04** | **0.04** | **0.04** | **0.04** | **0.04** | **0.03** |  |
| XA | - | <0.01 | <0.01 | <0.01 | <0.01 | <0.01 | <0.01 | <0.01 | <0.01 | <0.01 | <0.01 | <0.01 |  |
| XB | - | <0.01 | <0.01 | <0.01 | <0.01 | <0.01 | <0.01 | <0.01 | <0.01 | <0.01 | <0.01 | <0.01 |  |
| XC | - | <0.01 | <0.01 | <0.01 | <0.01 | <0.01 | <0.01 | <0.01 | <0.01 | <0.01 | <0.01 | <0.01 |  |
| XD | - | - | - | - | - | - | - | - | - | - | - | - |  |
| XE | - | 0.03 | 0.03 | 0.03 | 0.03 | 0.04 | 0.03 | 0.03 | 0.03 | 0.03 | 0.03 | 0.02 |  |
| XX | - | 0.01 | 0.01 | 0.01 | 0.01 | 0.01 | 0.01 | <0.01 | <0.01 | <0.01 | 0.01 | 0.01 |  |
| **Estonia** | **0.28** | **0.24** | **0.11** | **0.10** | **0.19** | **0.19** | **0.20** | **0.19** | **0.19** | **0.20** | **0.20** | **0.11** |  |
| XA | <0.01 | - | <0.01 | - | - | - | <0.01 | - | <0.01 | - | - | <0.01 |  |
| XB | - | - | <0.01 | - | - | <0.01 | - | <0.01 | <0.01 | <0.01 | <0.01 | <0.01 |  |
| XC | - | - | - | - | - | - | - | - | - | - | - | - |  |
| XD | 0.18 | 0.14 | 0.01 | <0.01 | 0.10 | 0.10 | 0.10 | 0.09 | 0.08 | 0.09 | 0.09 | <0.01 |  |
| XE | 0.09 | 0.10 | 0.10 | 0.10 | 0.09 | 0.09 | 0.10 | 0.10 | 0.11 | 0.11 | 0.11 | 0.11 |  |
| XX | <0.01 | <0.01 | <0.01 | <0.01 | - | - | - | - | - | <0.01 | - | - |  |
| **Finland** | - | - | **0.09** | **0.09** | **0.08** | **0.07** | **0.09** | **0.08** | **0.08** | **0.08** | **0.07** | **0.07** |  |
| XA | - | - | <0.01 | <0.01 | <0.01 | <0.01 | <0.01 | <0.01 | <0.01 | <0.01 | <0.01 | <0.01 |  |
| XB | - | - | - | - | - | - | - | - | - | - | - | - |  |
| XC | - | - | <0.01 | <0.01 | <0.01 | <0.01 | <0.01 | <0.01 | <0.01 | <0.01 | <0.01 | <0.01 |  |
| XD | - | - | - | - | - | <0.01 | - | - | - | - | - | <0.01 |  |
| XE | - | - | 0.06 | 0.06 | 0.05 | 0.04 | 0.05 | 0.05 | 0.05 | 0.05 | 0.05 | 0.05 |  |
| XX | - | - | 0.03 | 0.03 | 0.03 | 0.03 | 0.03 | 0.03 | 0.03 | 0.03 | 0.03 | 0.02 |  |
| Country, communityl consumption of other antibacterials (J01X); XA, consumption of glycopeptide antibacterials (J01XA); XB, consumption of polymyxins (J01XB); XC, consumption of steroid antibacterials (J01XC); XD, consumption of imidazole derivatives (J01XD); XE, consumption of nitrofuran derivatives (J01XE); XX, consumption of other antibacterials (J01XX); **-**, no consumption reported; Numbers reported in *italic* are total care data, i.e. community and hospital sector combined; ^a^Data for Belgium are slightly overestimated from 2016 onwards (nursing homes counting units versus packages before 2016); ^b^Data for Ireland do not include nitrofurantoin (J01XE01) consumption; ^c^Data for the Netherlands are based on average package size; ^d^Data for Spain include private prescriptions from 2016 onwards. | | | | | | | | | | | | | |
| **France** | - | - | - | - | **0.24** | **0.24** | **0.24** | **0.24** | **0.23** | **0.26** | **0.25** | **0.28** |  |
| XA | - | - | - | - | - | - | - | - | - | - | - | - |  |
| XB | - | - | - | - | - | 0.02 | 0.01 | 0.01 | 0.01 | 0.01 | 0.01 | 0.01 |  |
| XC | - | - | - | - | 0.05 | 0.05 | 0.05 | 0.04 | 0.04 | 0.04 | 0.04 | 0.03 |  |
| XD | - | - | - | - | - | - | - | - | - | - | - | <0.01 |  |
| XE | - | - | - | - | 0.07 | 0.05 | 0.04 | 0.04 | 0.03 | 0.04 | 0.04 | 0.05 |  |
| XX | - | - | - | - | 0.12 | 0.12 | 0.14 | 0.15 | 0.14 | 0.18 | 0.17 | 0.20 |  |
| **Greece** | ***0.84*** | ***0.93*** | ***1.00*** | **0.22** | ***0.91*** | **0.23** | **0.21** | **0.21** | **0.24** | **0.34** | **0.44** | **0.50** |  |
| XA | *0.20* | *0.23* | *0.24* | 0.05 | *0.22* | 0.06 | 0.05 | 0.05 | 0.06 | 0.07 | 0.10 | 0.11 |  |
| XB | *0.12* | *0.17* | *0.20* | 0.04 | *0.16* | 0.05 | 0.05 | 0.04 | 0.04 | 0.04 | 0.04 | 0.05 |  |
| XC | *0.05* | *0.06* | *0.06* | 0.02 | *0.03* | 0.01 | 0.01 | 0.01 | 0.01 | 0.01 | 0.01 | 0.01 |  |
| XD | *0.41* | *0.41* | *0.43* | 0.08 | *0.40* | 0.07 | 0.07 | 0.07 | 0.08 | 0.15 | 0.21 | 0.23 |  |
| XE | *0.02* | *0.02* | *0.02* | 0.02 | *0.03* | 0.03 | 0.03 | 0.04 | 0.04 | 0.04 | 0.04 | 0.04 |  |
| XX | *0.04* | *0.04* | *0.05* | 0.01 | *0.06* | 0.01 | 0.01 | 0.01 | 0.02 | 0.03 | 0.04 | 0.05 |  |
| **Iceland** | - | - | - | - | ***0.12*** | ***0.13*** | ***0.12*** | ***0.14*** | **0.07** | **0.08** | **0.09** | **0.10** |  |
| XA | - | - | - | - | *0.05* | *0.04* | *0.05* | *0.06* | - | <0.01 | <0.01 | <0.01 |  |
| XB | - | - | - | - | *-* | *-* | *-* | *-* | - | - | - | - |  |
| XC | - | - | - | - | *-* | *-* | *-* | *-* | - | - | - | - |  |
| XD | - | - | - | - | *0.01* | *0.01* | *0.01* | *0.01* | - | - | - | - |  |
| XE | - | - | - | - | *0.05* | *0.07* | *0.06* | *0.06* | 0.06 | 0.07 | 0.08 | 0.09 |  |
| XX | - | - | - | - | *0.01* | *0.01* | *0.01* | *0.01* | 0.01 | 0.01 | 0.01 | 0.01 |  |
| **Ireland^b^** | - | **0.03** | - | - | **0.03** | **0.03** | **0.02** | **0.02** | **0.02** | **0.02** | **0.02** | **0.02** |  |
| XA | - | <0.01 | - | - | <0.01 | <0.01 | <0.01 | <0.01 | <0.01 | <0.01 | <0.01 | 0.01 |  |
| XB | - | 0.02 | - | - | 0.02 | 0.02 | 0.02 | 0.02 | 0.01 | 0.01 | 0.01 | 0.01 |  |
| XC | - | <0.01 | - | - | <0.01 | <0.01 | <0.01 | <0.01 | <0.01 | <0.01 | <0.01 | <0.01 |  |
| XD | - | <0.01 | - | - | - | <0.01 | - | - | - | - | <0.01 | <0.01 |  |
| XE | - | <0.01 | - | - | <0.01 | - | - | - | - | - | <0.01 | - |  |
| XX | - | <0.01 | - | - | <0.01 | <0.01 | <0.01 | <0.01 | <0.01 | <0.01 | <0.01 | <0.01 |  |
| Country, community consumption of other antibacterials (J01X); XA, consumption of glycopeptide antibacterials (J01XA); XB, consumption of polymyxins (J01XB); XC, consumption of steroid antibacterials (J01XC); XD, consumption of imidazole derivatives (J01XD); XE, consumption of nitrofuran derivatives (J01XE); XX, consumption of other antibacterials (J01XX); **-**, no consumption reported; Numbers reported in *italic* are total care data, i.e. community and hospital sector combined; ^a^Data for Belgium are slightly overestimated from 2016 onwards (nursing homes counting units versus packages before 2016); ^b^Data for Ireland do not include nitrofurantoin (J01XE01) consumption; ^c^Data for the Netherlands are based on average package size; ^d^Data for Spain include private prescriptions from 2016 onwards. | | | | | | | | | | | | | |
| **Italy** | - | - | **0.27** | - | **0.28** | **0.27** | **0.28** | **0.29** | **0.29** | **0.29** | **0.29** | **0.27** |  |
| XA | - | - | 0.01 | - | 0.01 | 0.01 | 0.01 | 0.01 | 0.01 | 0.01 | 0.01 | 0.01 |  |
| XB | - | - | <0.01 | - | <0.01 | <0.01 | <0.01 | <0.01 | <0.01 | <0.01 | <0.01 | <0.01 |  |
| XC | - | - | - | - | - | - | - | - | - | - | - | - |  |
| XD | - | - | <0.01 | - | <0.01 | <0.01 | <0.01 | <0.01 | <0.01 | <0.01 | <0.01 | <0.01 |  |
| XE | - | - | 0.03 | - | 0.03 | 0.03 | 0.03 | 0.03 | 0.03 | 0.03 | 0.03 | 0.02 |  |
| XX | - | - | 0.23 | - | 0.23 | 0.22 | 0.23 | 0.24 | 0.25 | 0.25 | 0.25 | 0.23 |  |
| **Latvia** | - | - | - | - | **0.06** | **0.06** | **0.05** | **0.05** | **0.06** | **0.06** | **0.05** | **0.14** |  |
| XA | - | - | - | - | <0.01 | <0.01 | <0.01 | <0.01 | <0.01 | <0.01 | <0.01 | <0.01 |  |
| XB | - | - | - | - | - | - | <0.01 | <0.01 | <0.01 | <0.01 | <0.01 | <0.01 |  |
| XC | - | - | - | - | - | - | - | - | - | - | - | - |  |
| XD | - | - | - | - | 0.02 | 0.02 | 0.02 | 0.01 | 0.02 | 0.02 | 0.01 | 0.01 |  |
| XE | - | - | - | - | 0.02 | 0.02 | 0.02 | 0.02 | 0.03 | 0.03 | 0.03 | 0.12 |  |
| XX | - | - | - | - | 0.02 | 0.02 | 0.02 | 0.01 | 0.01 | 0.01 | 0.01 | 0.01 |  |
| **Lithuania** | - | ***0.29*** | ***0.36*** | ***0.29*** | ***0.28*** | ***0.26*** | **0.18** | **0.18** | **0.19** | **0.19** | **0.19** | **0.15** |  |
| XA | - | *0.01* | *0.03* | *0.02* | *0.03* | *0.03* | <0.01 | <0.01 | <0.01 | <0.01 | <0.01 | <0.01 |  |
| XB | - | *-* | *-* | *-* | *-* | *-* | - | - | - | - | - | - |  |
| XC | - | *-* | *-* | *-* | *-* | *-* | - | - | - | - | - | - |  |
| XD | - | *0.09* | *0.13* | *0.11* | *0.11* | *0.09* | 0.04 | 0.04 | 0.04 | 0.04 | 0.04 | 0.02 |  |
| XE | - | *0.14* | *0.15* | *0.12* | *0.12* | *0.12* | 0.13 | 0.13 | 0.14 | 0.14 | 0.14 | 0.11 |  |
| XX | - | *0.04* | *0.05* | *0.03* | *0.02* | *0.02* | 0.01 | 0.01 | 0.01 | 0.01 | 0.01 | 0.01 |  |
| **Luxembourg** | **0.08** | **0.09** | **0.11** | **0.11** | **0.11** | **0.13** | **0.13** | **0.14** | **0.15** | **0.15** | **0.16** | - |  |
| XA | <0.01 | <0.01 | 0.01 | <0.01 | <0.01 | <0.01 | <0.01 | <0.01 | <0.01 | <0.01 | - | - |  |
| XB | 0.01 | <0.01 | <0.01 | <0.01 | <0.01 | <0.01 | <0.01 | <0.01 | - | - | - | - |  |
| XC | <0.01 | <0.01 | <0.01 | <0.01 | - | - | - | - | - | - | - | - |  |
| XD | - | - | <0.01 | - | - | - | - | - | - | - | - | - |  |
| XE | 0.03 | 0.04 | 0.04 | 0.04 | 0.04 | 0.05 | 0.05 | 0.05 | 0.05 | 0.05 | 0.05 | - |  |
| XX | 0.04 | 0.05 | 0.06 | 0.06 | 0.07 | 0.07 | 0.07 | 0.09 | 0.10 | 0.10 | 0.11 | - |  |
| Country, community consumption of other antibacterials (J01X); XA, consumption of glycopeptide antibacterials (J01XA); XB, consumption of polymyxins (J01XB); XC, consumption of steroid antibacterials (J01XC); XD, consumption of imidazole derivatives (J01XD); XE, consumption of nitrofuran derivatives (J01XE); XX, consumption of other antibacterials (J01XX); **-**, no consumption reported; Numbers reported in *italic* are total care data, i.e. community and hospital sector combined; ^a^Data for Belgium are slightly overestimated from 2016 onwards (nursing homes counting units versus packages before 2016); ^b^Data for Ireland do not include nitrofurantoin (J01XE01) consumption; ^c^Data for the Netherlands are based on average package size; ^d^Data for Spain include private prescriptions from 2016 onwards. | | | | | | | | | | | | | |
| **Netherlands^c^** | - | - | **0.18** | **0.20** | - | - | - | - | - | - | - | - |  |
| XA | - | - | <0.01 | <0.01 | - | - | - | - | - | - | - | - |  |
| XB | - | - | <0.01 | <0.01 | - | - | - | - | - | - | - | - |  |
| XC | - | - | <0.01 | <0.01 | - | - | - | - | - | - | - | - |  |
| XD | - | - | <0.01 | <0.01 | - | - | - | - | - | - | - | - |  |
| XE | - | - | 0.17 | 0.19 | - | - | - | - | - | - | - | - |  |
| XX | - | - | 0.01 | 0.01 | - | - | - | - | - | - | - | - |  |
| **Portugal** | - | - | **0.09** | **0.11** | **0.11** | **0.15** | **0.16** | **0.12** | **0.14** | **0.14** | **0.15** | **0.16** |  |
| XA | - | - | - | - | - | - | - | - | - | - | - | - |  |
| XB | - | - | - | - | - | - | - | - | - | - | - | - |  |
| XC | - | - | 0.02 | 0.02 | 0.02 | 0.02 | 0.01 | 0.01 | 0.01 | 0.01 | 0.01 | 0.01 |  |
| XD | - | - | - | - | - | - | - | - | - | - | - | - |  |
| XE | - | - | 0.03 | 0.03 | 0.03 | 0.05 | 0.05 | 0.03 | 0.04 | 0.04 | 0.04 | 0.04 |  |
| XX | - | - | 0.05 | 0.06 | 0.06 | 0.08 | 0.10 | 0.08 | 0.09 | 0.10 | 0.11 | 0.12 |  |
| **Slovakia** | - | - | - | - | - | ***0.13*** | **0.03** | **0.03** | **0.04** | **0.04** | **0.05** | - |  |
| XA | - | - | - | - | - | *0.03* | <0.01 | <0.01 | <0.01 | <0.01 | <0.01 | - |  |
| XB | - | - | - | - | - | *<0.01* | <0.01 | <0.01 | <0.01 | <0.01 | <0.01 | - |  |
| XC | - | - | - | - | - | *-* | - | - | - | - | - | - |  |
| XD | - | - | - | - | - | *0.07* | <0.01 | <0.01 | 0.01 | 0.01 | 0.01 | - |  |
| XE | - | - | - | - | - | *-* | - | - | - | - | - | - |  |
| XX | - | - | - | - | - | *0.02* | 0.02 | 0.02 | 0.03 | 0.03 | 0.04 | - |  |
| **Slovenia** | - | **<0.01** | **<0.01** | **<0.01** | **<0.01** | **0.01** | **0.02** | **0.02** | **0.04** | **0.04** | **0.04** | **0.06** |  |
| XA | - | - | - | - | - | - | <0.01 | - | - | - | - | - |  |
| XB | - | - | - | - | - | - | - | <0.01 | <0.01 | <0.01 | <0.01 | <0.01 |  |
| XC | - | - | - | - | - | - | - | - | - | - | - | - |  |
| XD | - | - | - | - | - | - | - | - | - | - | - | - |  |
| XE | - | - | - | - | <0.01 | 0.01 | 0.02 | 0.02 | 0.04 | 0.04 | 0.04 | 0.06 |  |
| XX | - | <0.01 | <0.01 | <0.01 | - | - | <0.01 | <0.01 | <0.01 | <0.01 | <0.01 | <0.01 |  |
| Country, community consumption of other antibacterials (J01X); XA, consumption of glycopeptide antibacterials (J01XA); XB, consumption of polymyxins (J01XB); XC, consumption of steroid antibacterials (J01XC); XD, consumption of imidazole derivatives (J01XD); XE, consumption of nitrofuran derivatives (J01XE); XX, consumption of other antibacterials (J01XX); **-**, no consumption reported; Numbers reported in *italic* are total care data, i.e. community and hospital sector combined; ^a^Data for Belgium are slightly overestimated from 2016 onwards (nursing homes counting units versus packages before 2016); ^b^Data for Ireland do not include nitrofurantoin (J01XE01) consumption; ^c^Data for the Netherlands are based on average package size; ^d^Data for Spain include private prescriptions from 2016 onwards. | | | | | | | | | | | | | |
| **Spain^d^** | - | - | - | - | **0.15** | **0.16** | **0.15** | **0.16** | **0.17** | **0.18** | **0.19** | **0.20** |  |
| XA | - | - | - | - | <0.01 | <0.01 | <0.01 | <0.01 | <0.01 | <0.01 | <0.01 | <0.01 |  |
| XB | - | - | - | - | - | - | - | - | - | - | - | - |  |
| XC | - | - | - | - | <0.01 | <0.01 | <0.01 | <0.01 | <0.01 | <0.01 | <0.01 | <0.01 |  |
| XD | - | - | - | - | <0.01 | <0.01 | <0.01 | - | - | - | - | - |  |
| XE | - | - | - | - | 0.01 | 0.01 | 0.01 | 0.01 | 0.01 | 0.02 | 0.01 | 0.01 |  |
| XX | - | - | - | - | 0.13 | 0.14 | 0.14 | 0.15 | 0.16 | 0.16 | 0.17 | 0.18 |  |
| **Sweden** | - | - | - | **0.09** | **0.09** | **0.10** | **0.10** | **0.10** | **0.10** | **0.10** | **0.10** | **0.10** |  |
| XA | - | - | - | <0.01 | <0.01 | <0.01 | <0.01 | <0.01 | <0.01 | <0.01 | <0.01 | <0.01 |  |
| XB | - | - | - | <0.01 | <0.01 | <0.01 | <0.01 | <0.01 | <0.01 | <0.01 | <0.01 | <0.01 |  |
| XC | - | - | - | <0.01 | <0.01 | <0.01 | <0.01 | <0.01 | <0.01 | <0.01 | <0.01 | <0.01 |  |
| XD | - | - | - | - | - | - | - | - | - | - | - | - |  |
| XE | - | - | - | 0.05 | 0.06 | 0.07 | 0.07 | 0.07 | 0.07 | 0.07 | 0.07 | 0.08 |  |
| XX | - | - | - | 0.03 | 0.03 | 0.03 | 0.03 | 0.03 | 0.03 | 0.03 | 0.02 | 0.03 |  |
| **UK** | - | - | - | - | - | - | - | - | - | **0.01** | - | - |  |
| XA | - | - | - | - | - | - | - | - | - | - | - | - |  |
| XB | - | - | - | - | - | - | - | - | - | <0.01 | - | - |  |
| XC | - | - | - | - | - | - | - | - | - | - | - | - |  |
| XD | - | - | - | - | - | - | - | - | - | - | - | - |  |
| XE | - | - | - | - | - | - | - | - | - | 0.01 | - | - |  |
| XX | - | - | - | - | - | - | - | - | - | - | - | - |  |

Country, community consumption of other antibacterials (J01X); XA, consumption of glycopeptide antibacterials (J01XA); XB, consumption of polymyxins (J01XB); XC, consumption of steroid
antibacterials (J01XC); XD, consumption of imidazole derivatives (J01XD); XE, consumption of nitrofuran derivatives (J01XE); XX, consumption of other antibacterials (J01XX); **-**, no consumption
reported; Numbers reported in *italic* are total care data,. i.e. community and hospital sector combined; ^a^Data for Belgium are slightly overestimated from 2016 onwards (nursing homes counting units versus packages before 2016); ^b^Data for Ireland do not include nitrofurantoin (J01XE01) consumption; ^c^Data for the Netherlands are based on average package size; ^d^Data for Spain include private prescriptions from 2016 onwards.

**
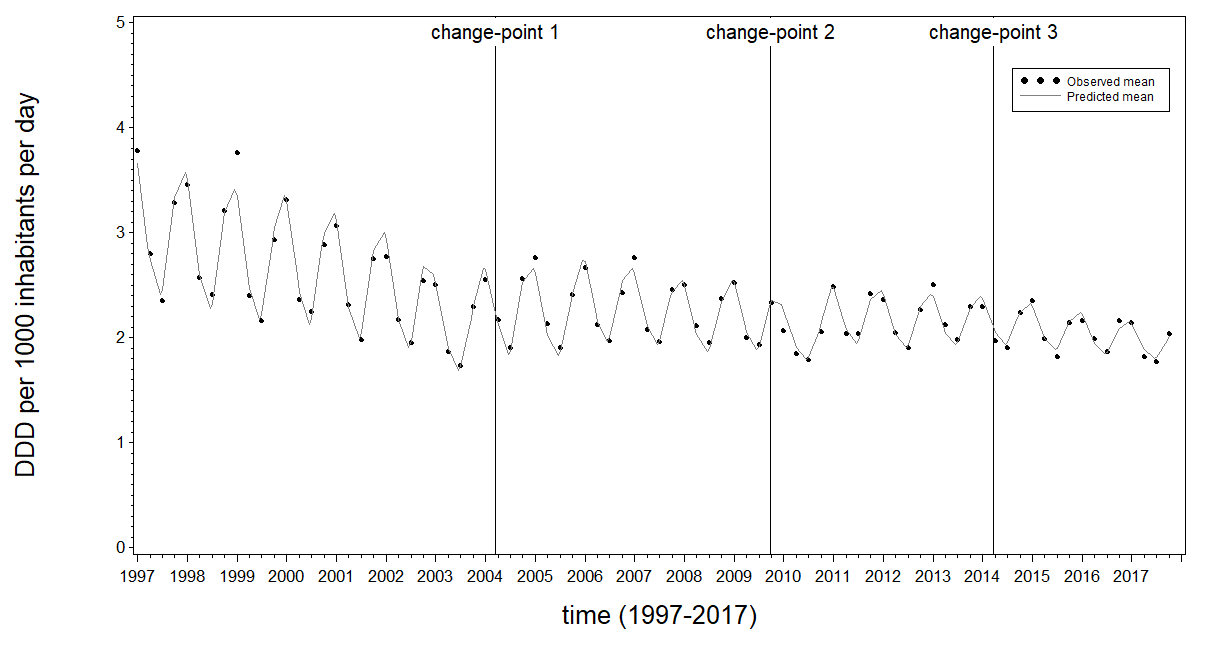
Figure S1. Average of observed (dots) and predicted (solid line) consumption of tetracyclines (ATC J01A) in the community expressed in DDD (ATC/DDD index 2019) per 1000 inhabitants per day and based on quarterly data, 25 EU/EEA countries, 1997-2017.**


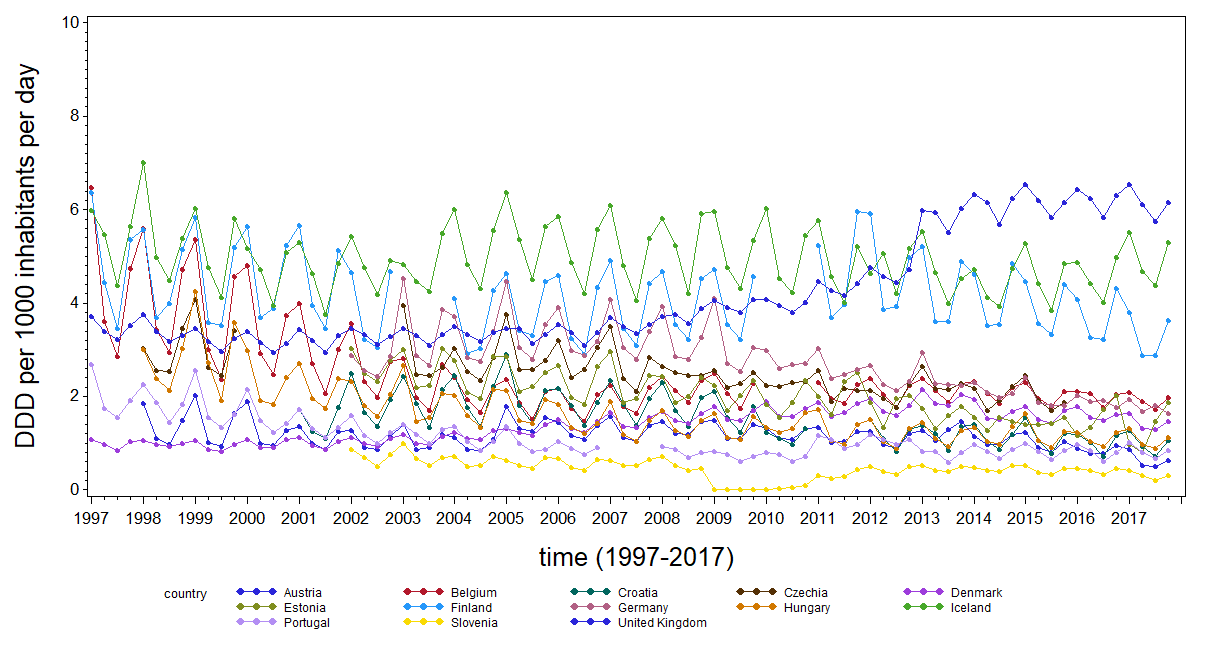


**Figure S2. Seasonal variation in consumption of tetracyclines (ATC J01A) in the community, expressed in DDD (ATC/DDD index 2019) per 1000 inhabitants per day, 13 EU/EEA countries reporting consumption per quarter for at least 15 years, 1997-2017.**


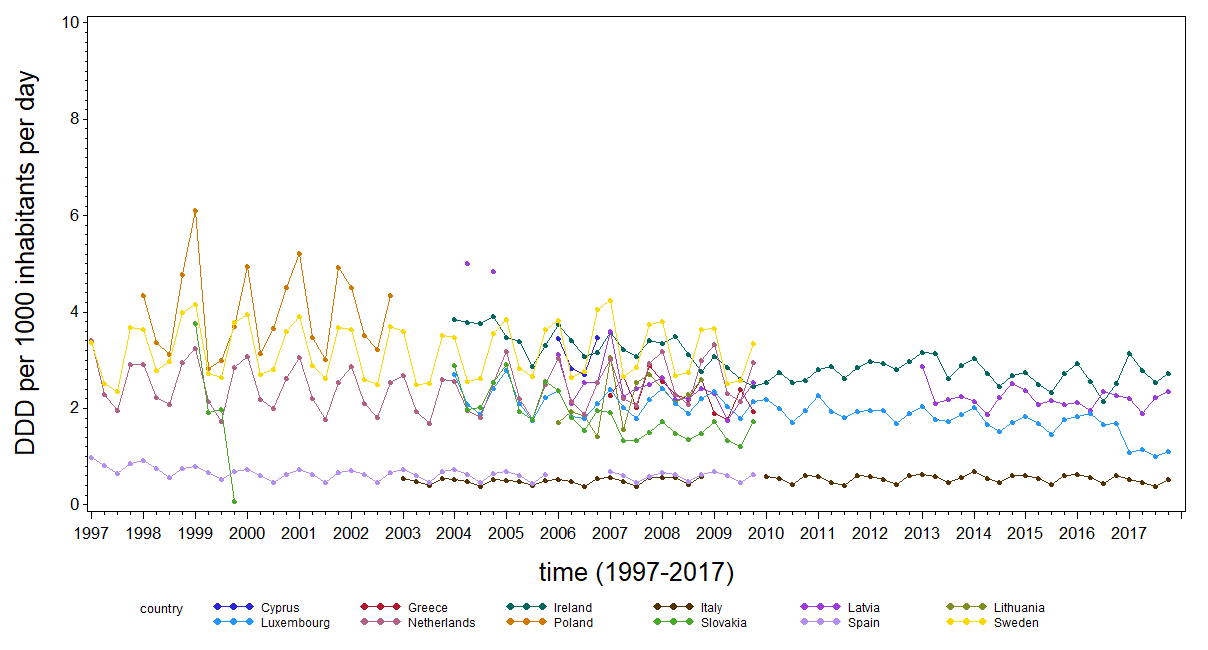


**Figure S3. Seasonal variation in consumption of tetracyclines (ATC J01A) in the community, expressed in DDD (ATC/DDD index 2019) per 1000 inhabitants per day, 12 EU countries reporting consumption per quarter for less than 15 years, 1997-2017. For Cyprus, total care data, i.e. community and hospital sector combined, are used. For Spain, private prescriptions are included from 2016 onwards.**

**
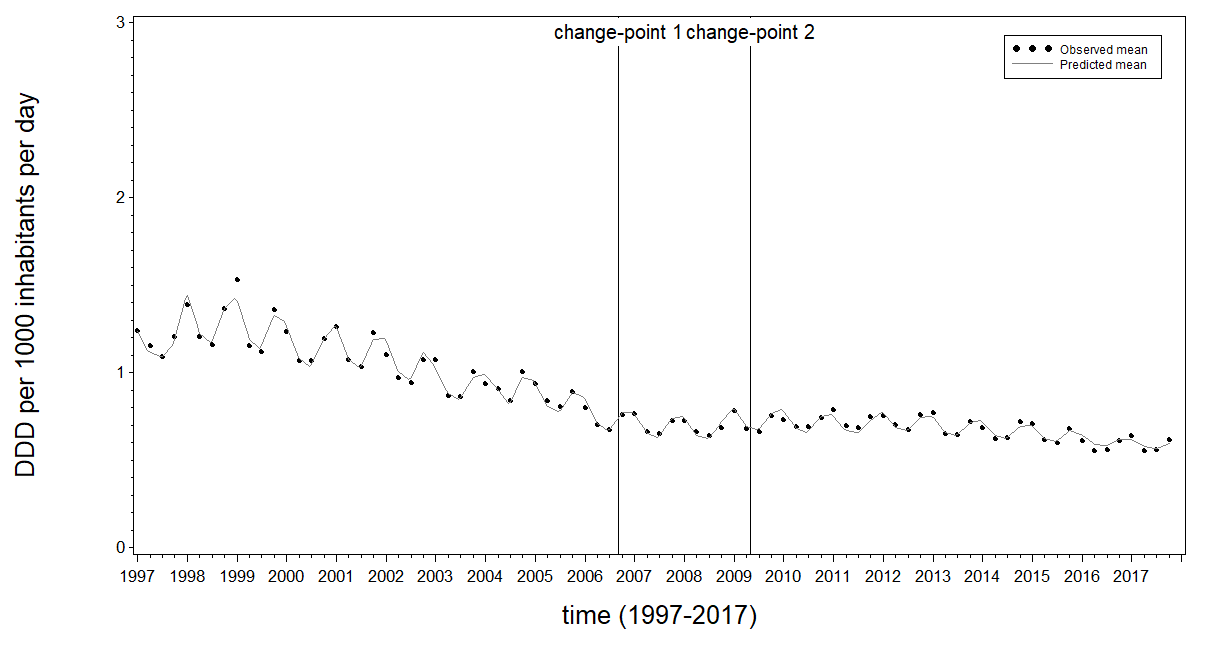
Figure S4. Average of observed (dots) and predicted (solid line) consumption of sulfonamides and trimethoprim (ATC J01E) in the community expressed in DDD (ATC/DDD index 2019) per 1000 inhabitants per day and based on quarterly data, 25 EU/EEA countries, 1997-2017.**

**
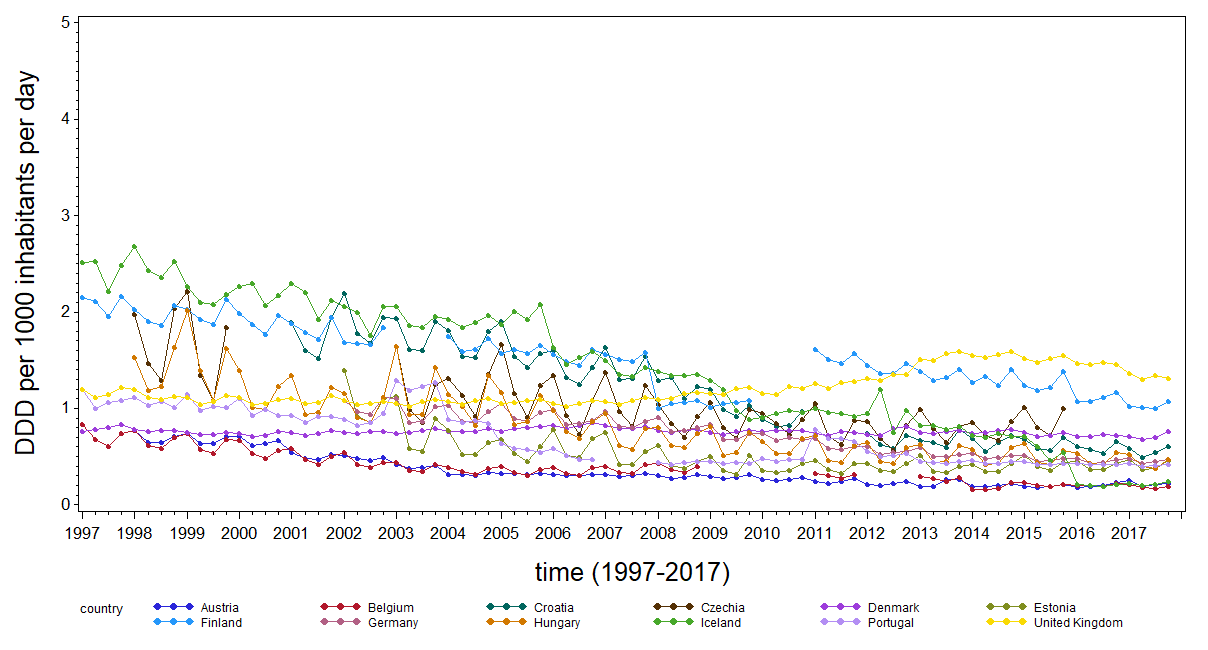
**

**Figure S5. Seasonal variation in consumption of sulfonamides and trimethoprim (ATC J01E) in the community, expressed in DDD (ATC/DDD index 2019) per 1000 inhabitants per day, 13 EU/EEA countries reporting consumption per quarter for at least 15 years, 1997-2017.**

**
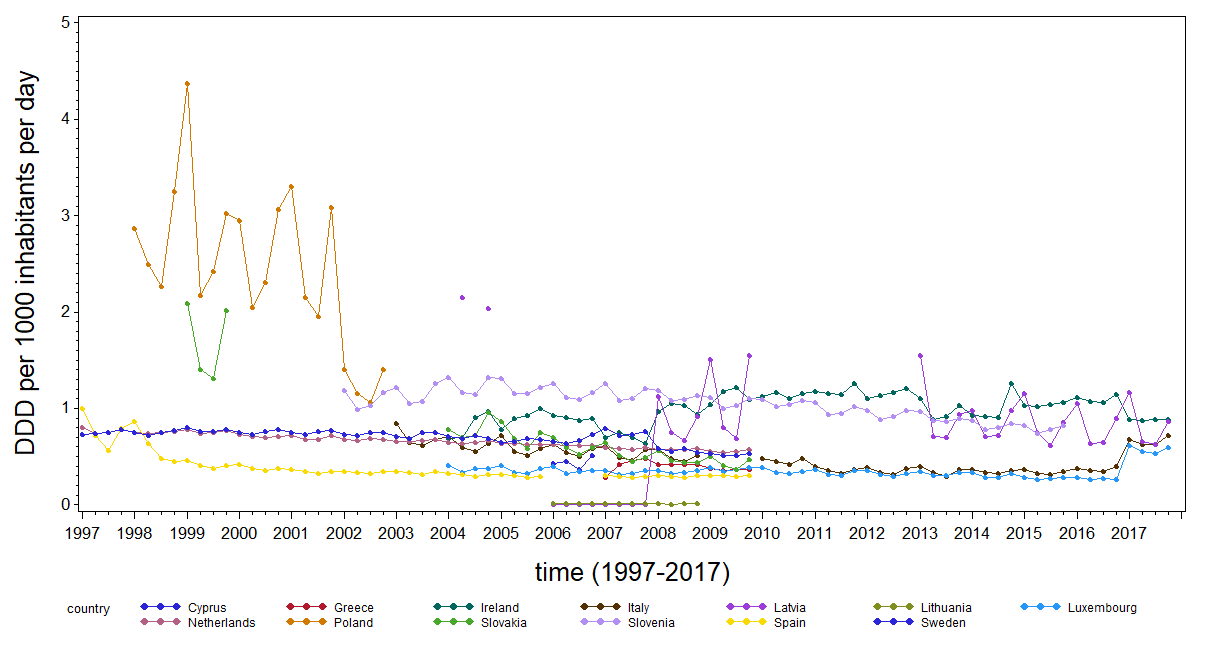
**

**Figure S6. Seasonal variation in consumption of sulfonamides and trimethoprim (ATC J01E) in the community, expressed in DDD (ATC/DDD index 2019) per 1000 inhabitants per day, 12 EU countries reporting consumption per quarter for less than 15 years, 1997-2017. For Cyprus, total care data, i.e. community and hospital sector combined, are used. For Spain, private prescriptions are included from 2016 onwards.**

^
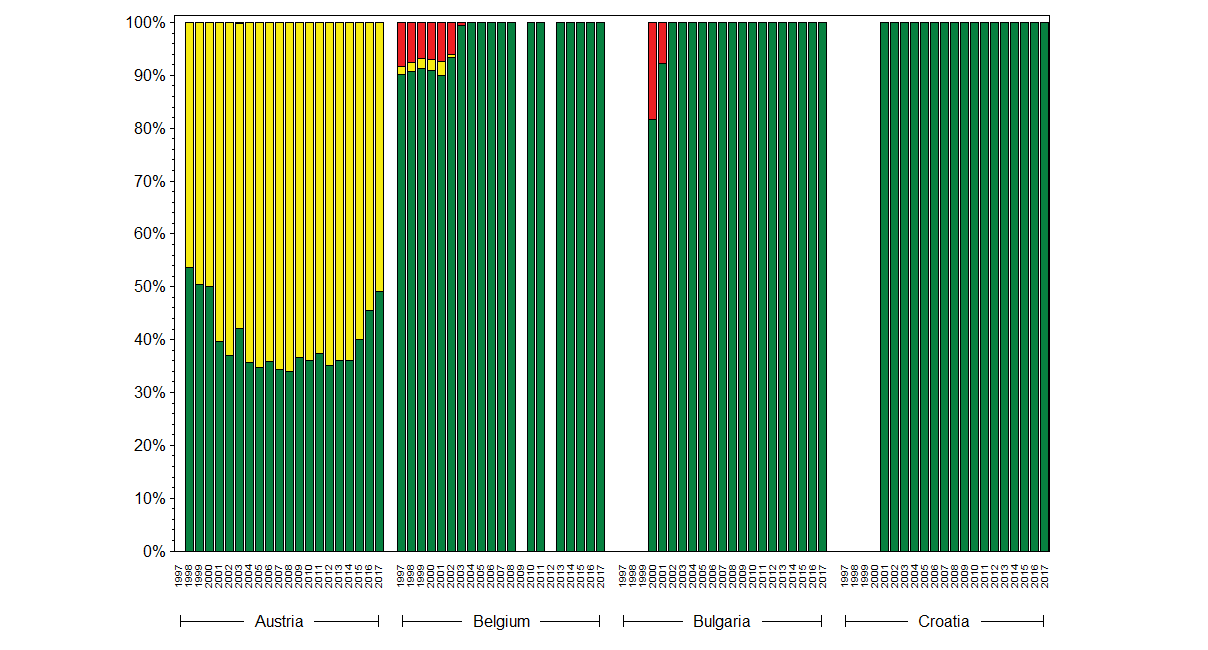
^

^
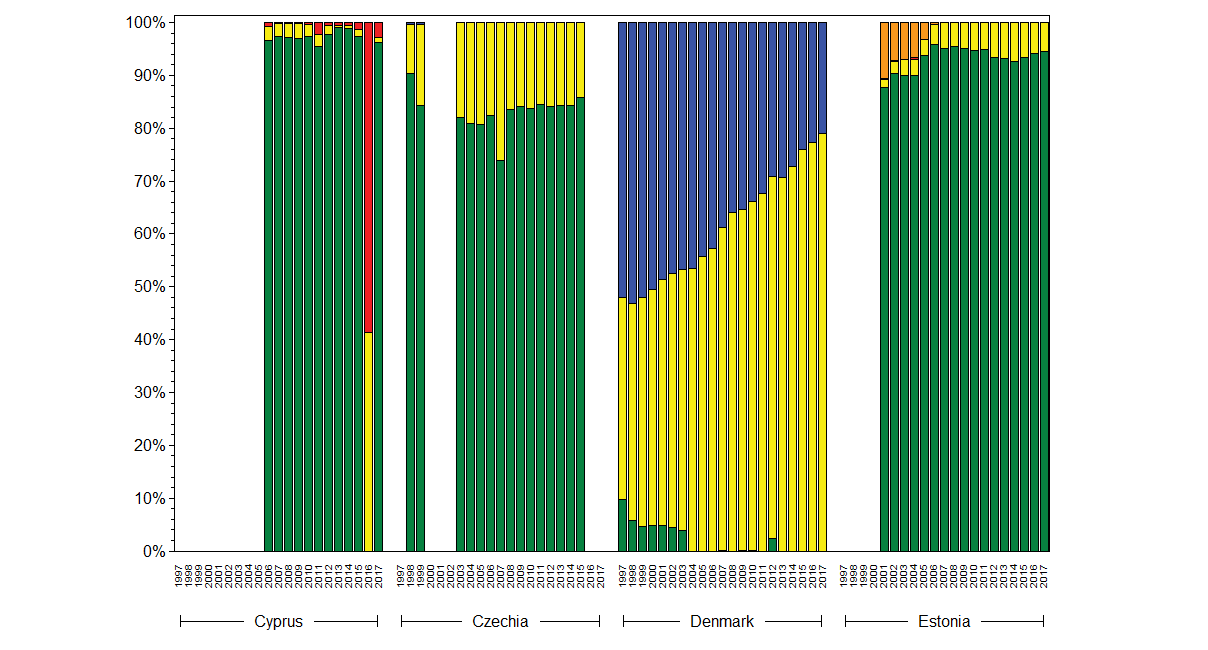
^

= combinations of sulfonamides and trimethoprim (J01EE), = trimethoprim and derivatives (J01EA),
 = short-acting sulfonamides (J01EB), = intermediate-acting sulfonamides (J01EC),
 = long-acting sulfonamides (J01ED)

**Figure S7. Composition of consumption of sulfonamides and trimethoprim (ATC J01E) in the community, expressed in DDD (ATC/DDD index 2019) per 1000 inhabitants per day 30 EU/EEA countries, 1997-2017. For Cyprus and Romania, total care data, i.e. community and hospital sector combined, are used. For Spain, private prescription are included from 2016 onwards. For Romania, data have a coverage in 2009 limited to 30-40%.**

^
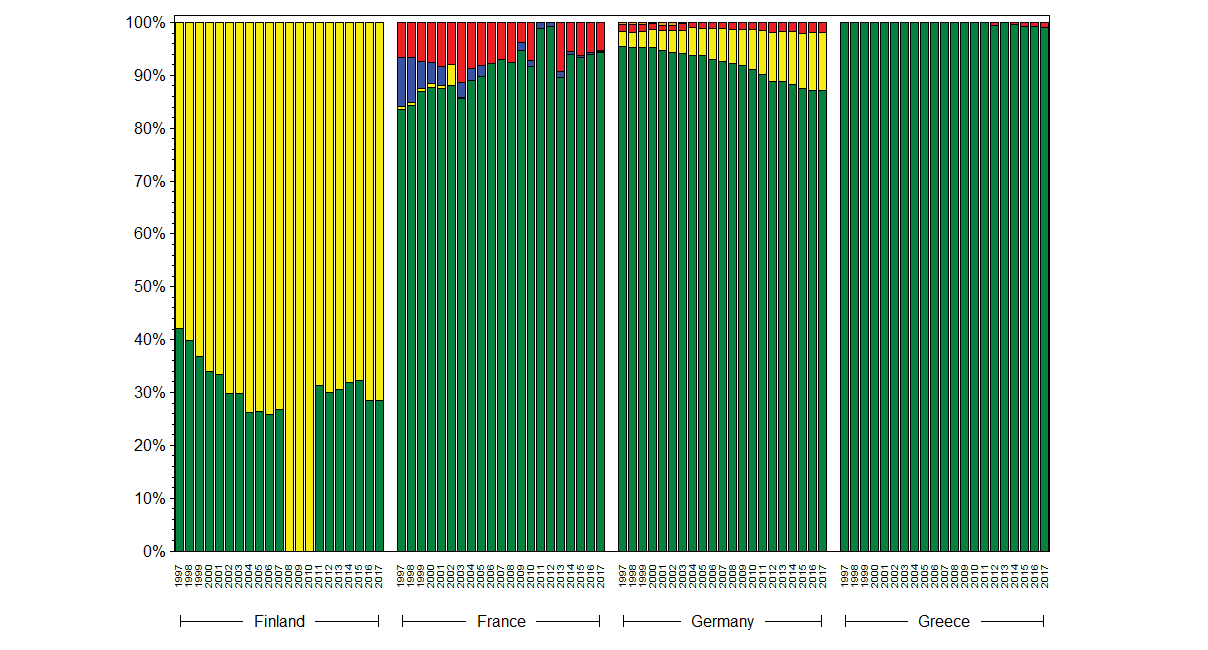
^

^
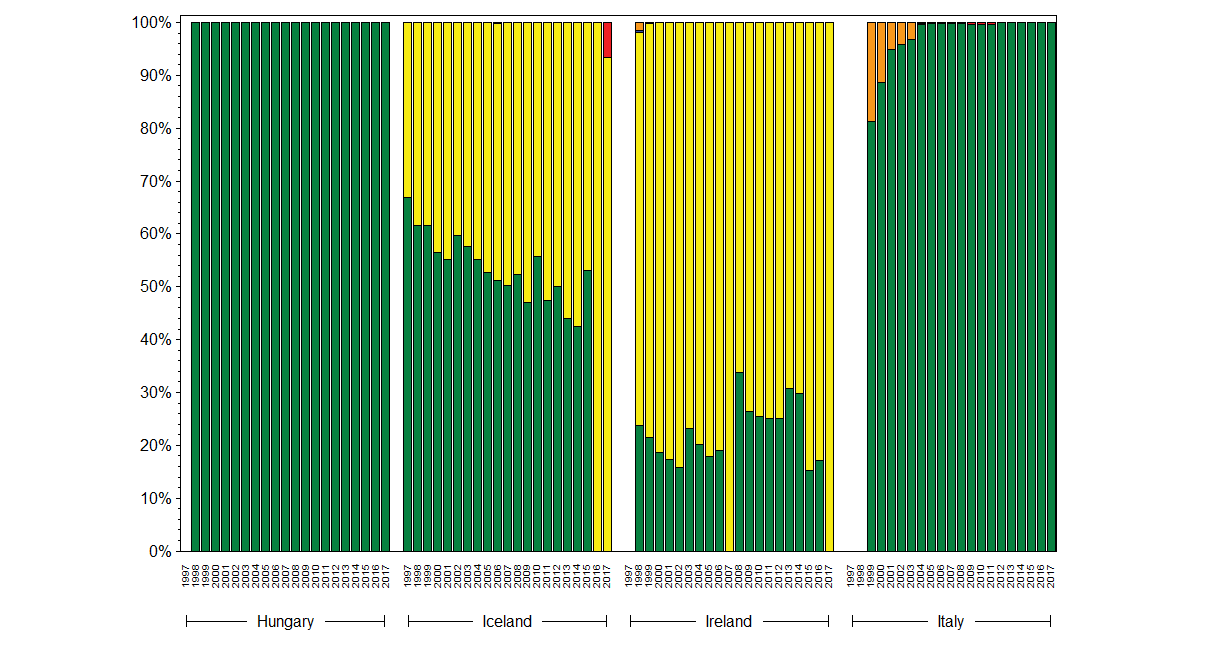
^

= combinations of sulfonamides and trimethoprim (J01EE), = trimethoprim and derivatives (J01EA),
 = short-acting sulfonamides (J01EB), = intermediate-acting sulfonamides (J01EC),
 = long-acting sulfonamides (J01ED)

**Figure S7.** Continued

^
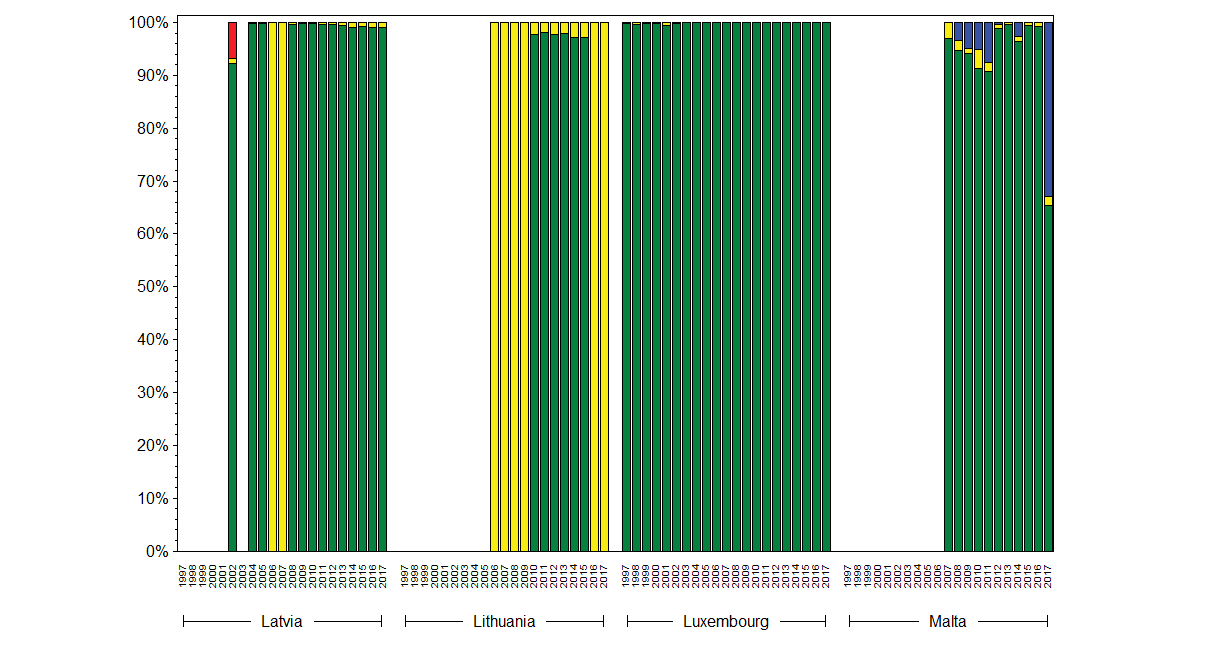
^

^
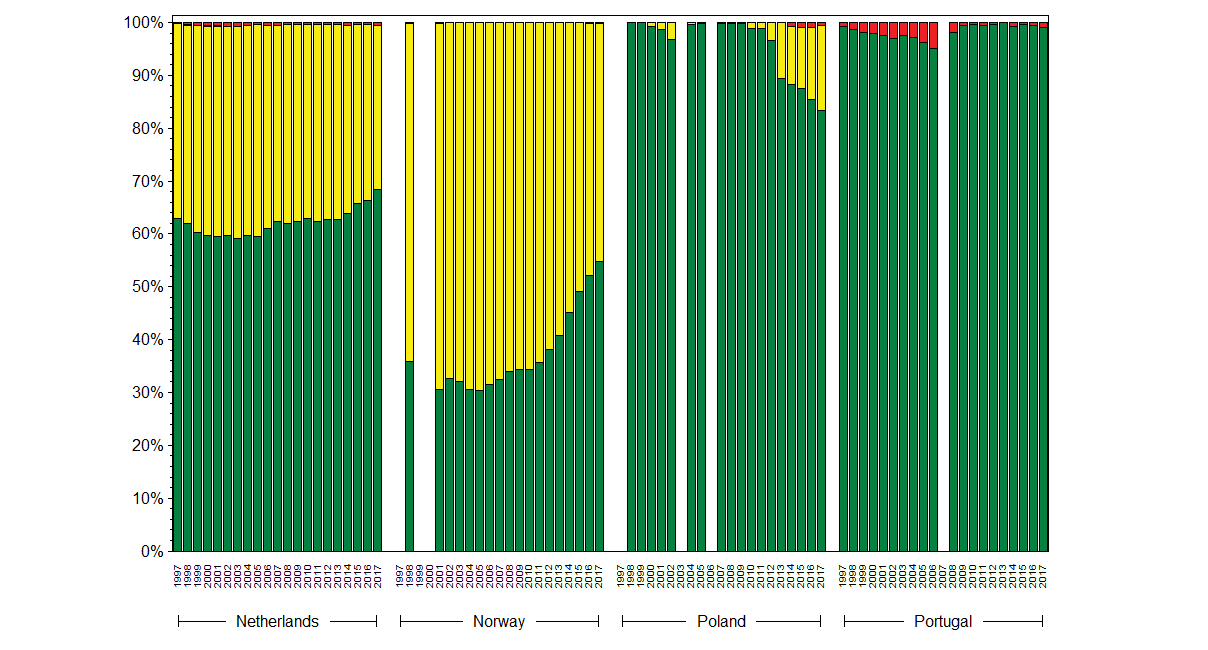
^

= combinations of sulfonamides and trimethoprim (J01EE), = trimethoprim and derivatives (J01EA),
 = short-acting sulfonamides (J01EB), = intermediate-acting sulfonamides (J01EC),
 = long-acting sulfonamides (J01ED)

**Figure S7.** Continued

^
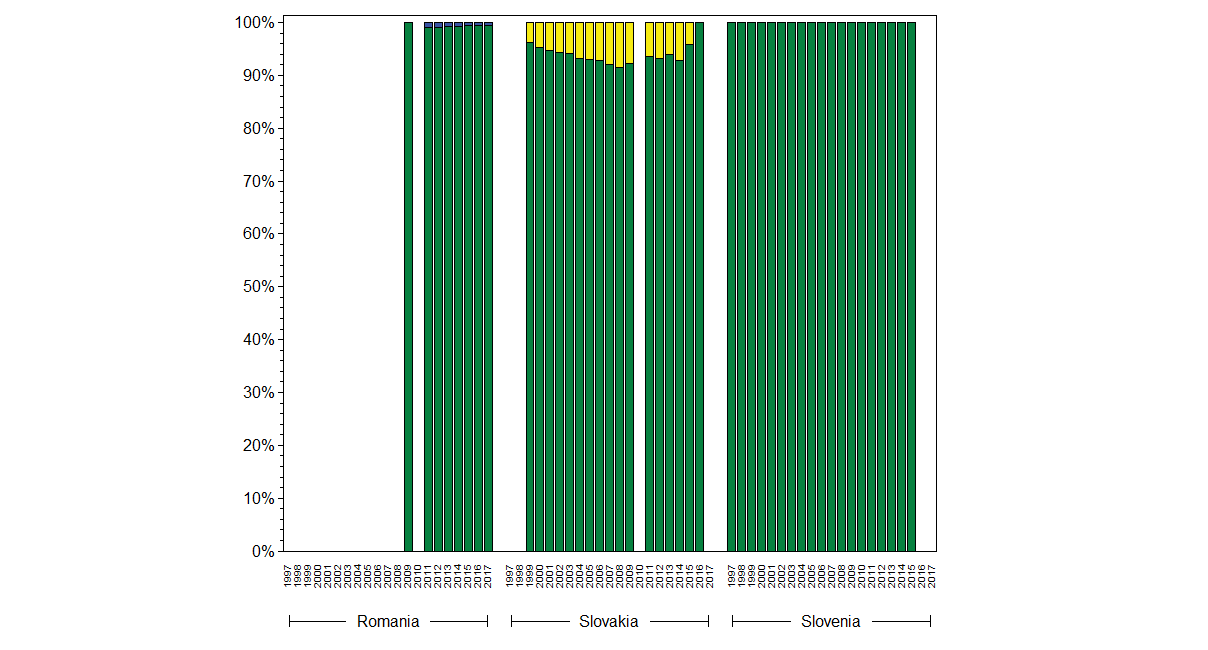
^

^
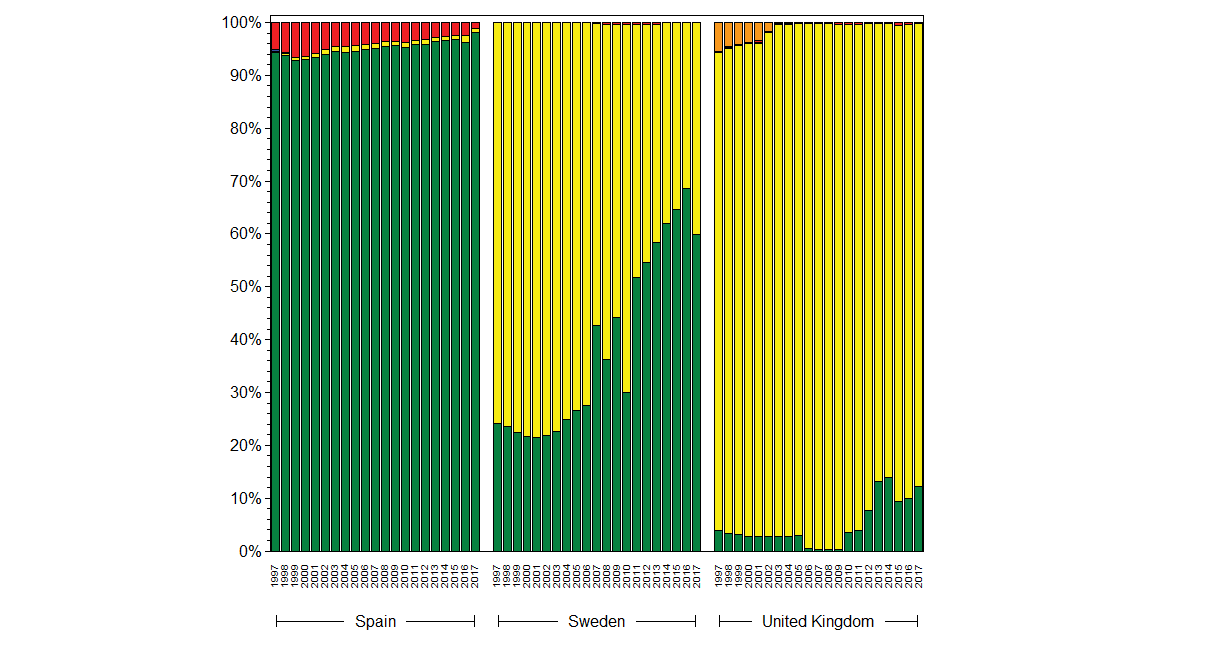
^

= combinations of sulfonamides and trimethoprim (J01EE), = trimethoprim and derivatives (J01EA),
 = short-acting sulfonamides (J01EB), = intermediate-acting sulfonamides (J01EC),
 = long-acting sulfonamides (J01ED)

**Figure S7.** Continued

**
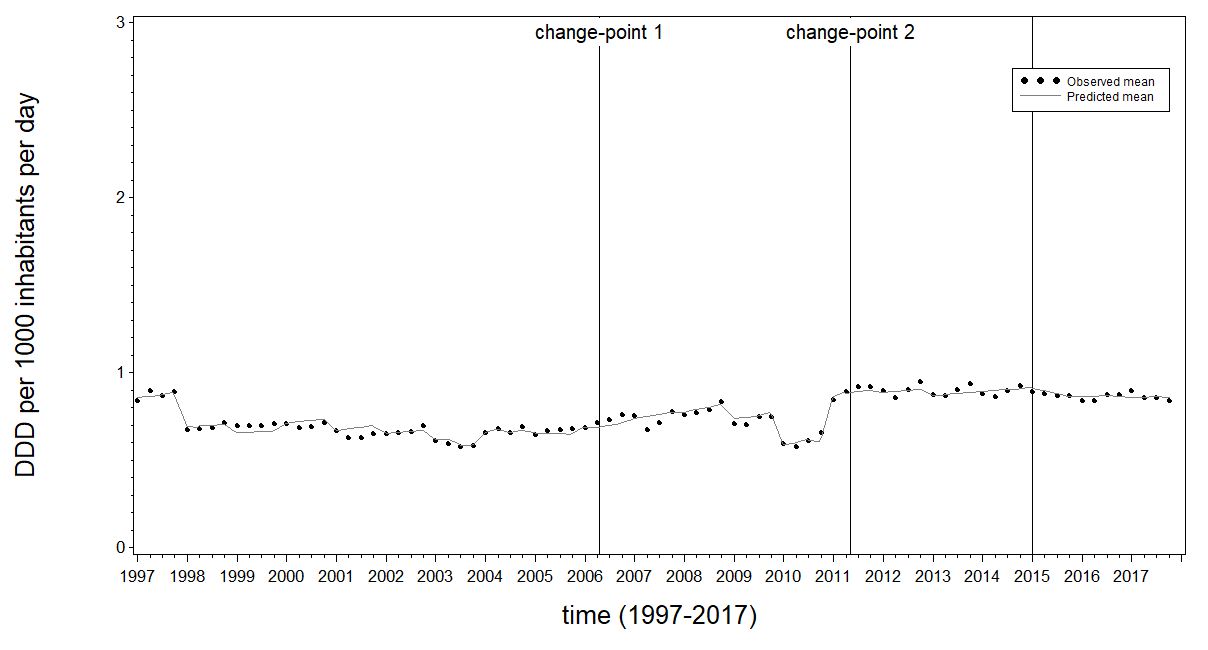
Figure S8. Average of observed (dots) and predicted (solid line) consumption of other antibacterials (ATC J01X) in the community expressed in DDD (ATC/DDD index 2019) per 1000 inhabitants per day and based on quarterly data, 25 EU/EEA countries, 1997-2017.**

**
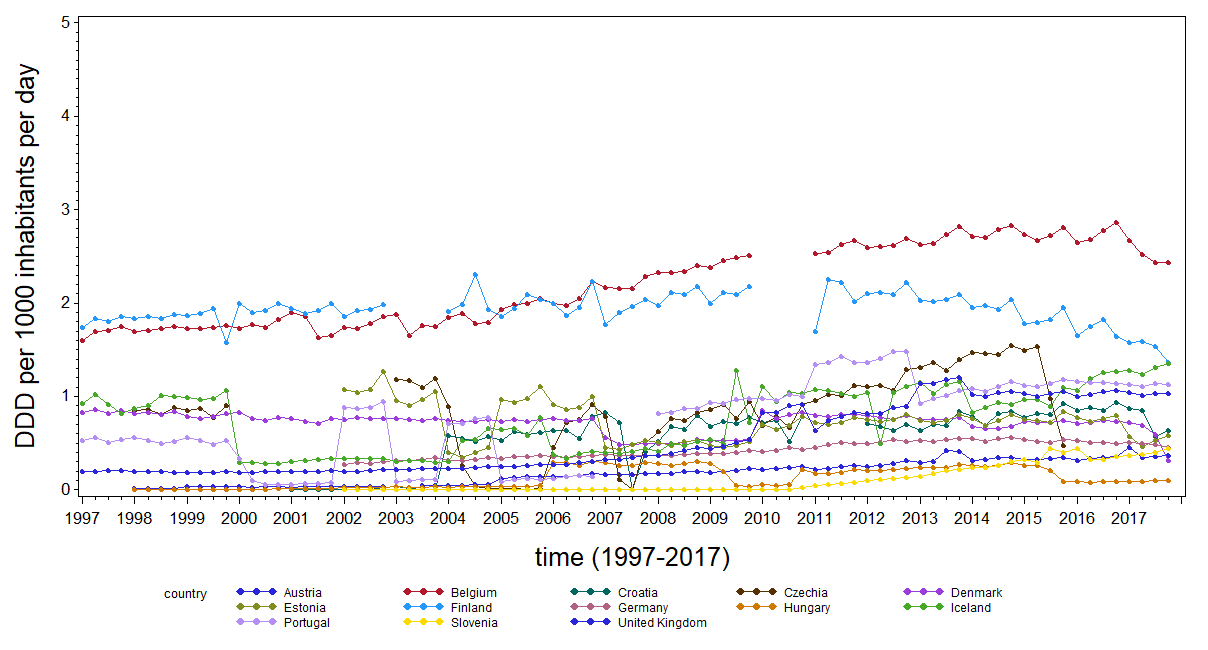
**

**Figure S9. Seasonal variation in consumption of other antibacterials (ATC J01X) in the community, expressed in DDD (ATC/DDD index 2019) per 1000 inhabitants per day, 13 EU/EEA countries reporting consumption per quarter for at least 15 years, 1997-2017.**

**
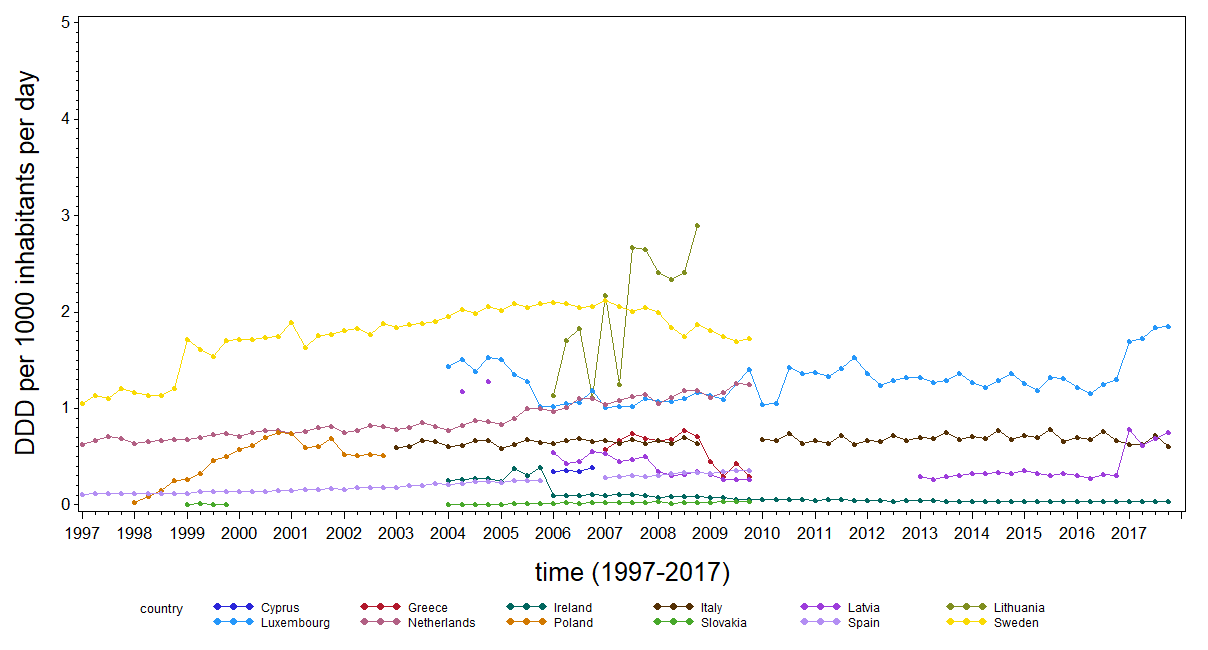
**

**Figure S10. Seasonal variation in consumption of other antibacterials (ATC J01X) in the community, expressed in DDD (ATC/DDD index 2019) per 1000 inhabitants per day, 12 EU countries reporting consumption per quarter for less than 15 years, 1997-2017. For Cyprus, total care data, i.e. community and hospital sector combined, are used. For Ireland, nitrofurantoin (J01XE01) consumption is not included. For Spain, private prescriptions are included from 2016 onwards.**


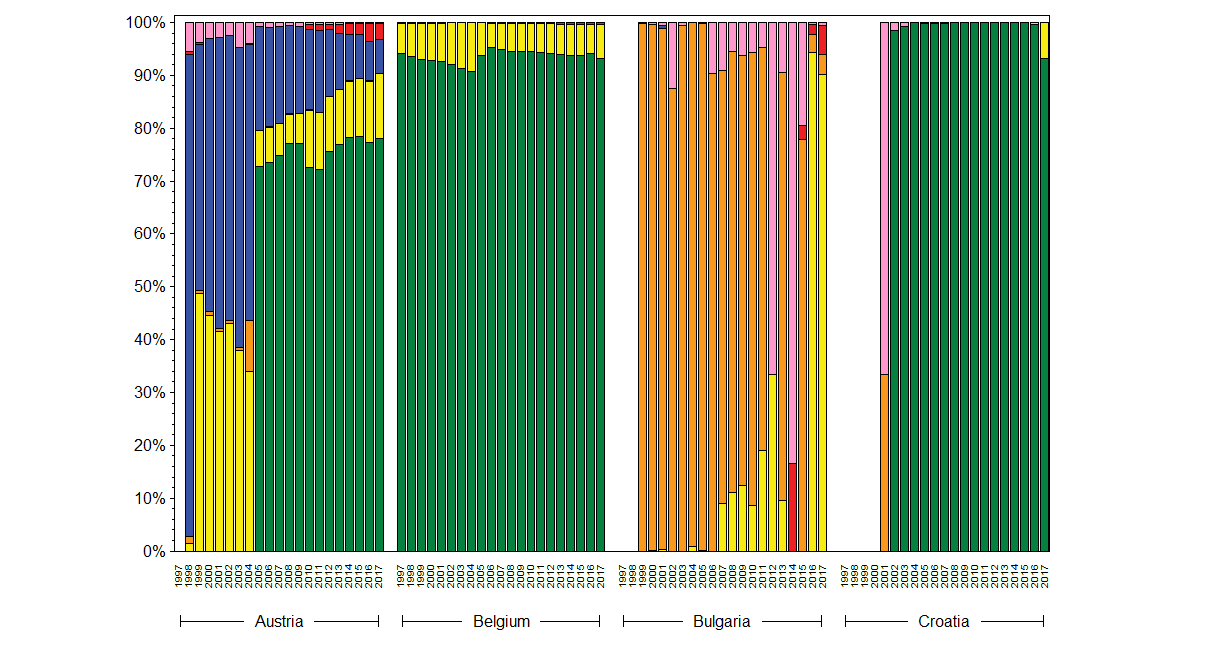


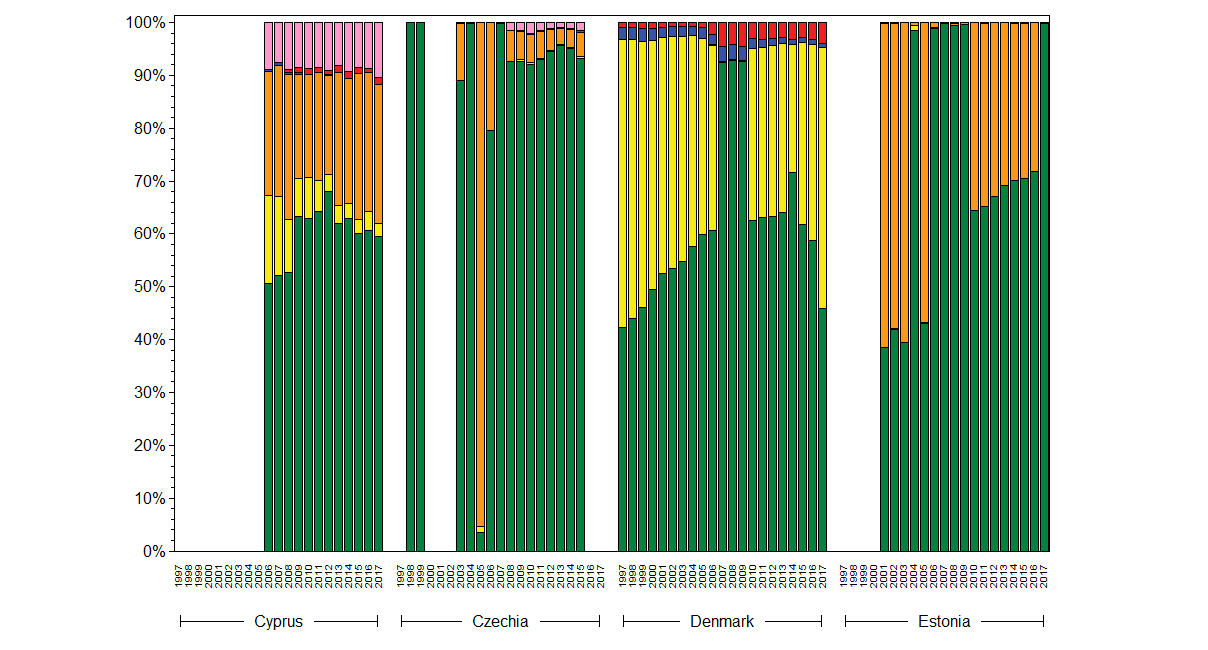


= nitrofuran derivatives (J01XE), = other antibacterials (J01XX), = imidazole derivatives (J01XD),

= steroid antibacterials (J01XC), = polymyxins (J01XB), = glycopeptide antibacterials (J01XA)

**Figure S11. Composition of consumption of other antibacterials (J01X) in the community, expressed in DDD (ATC/DDD index 2019) per 1000 inhabitants per day, in 30 EU/EEA countries, 1997-2017. For Cyprus and Romania, total care data are used. For Ireland, nitrofurantoin (J01XE01) consumption was not included. For Spain, private prescription were included from 2016 onwards. For Romania, data have a coverage in 2009 limited to 30-40%.**


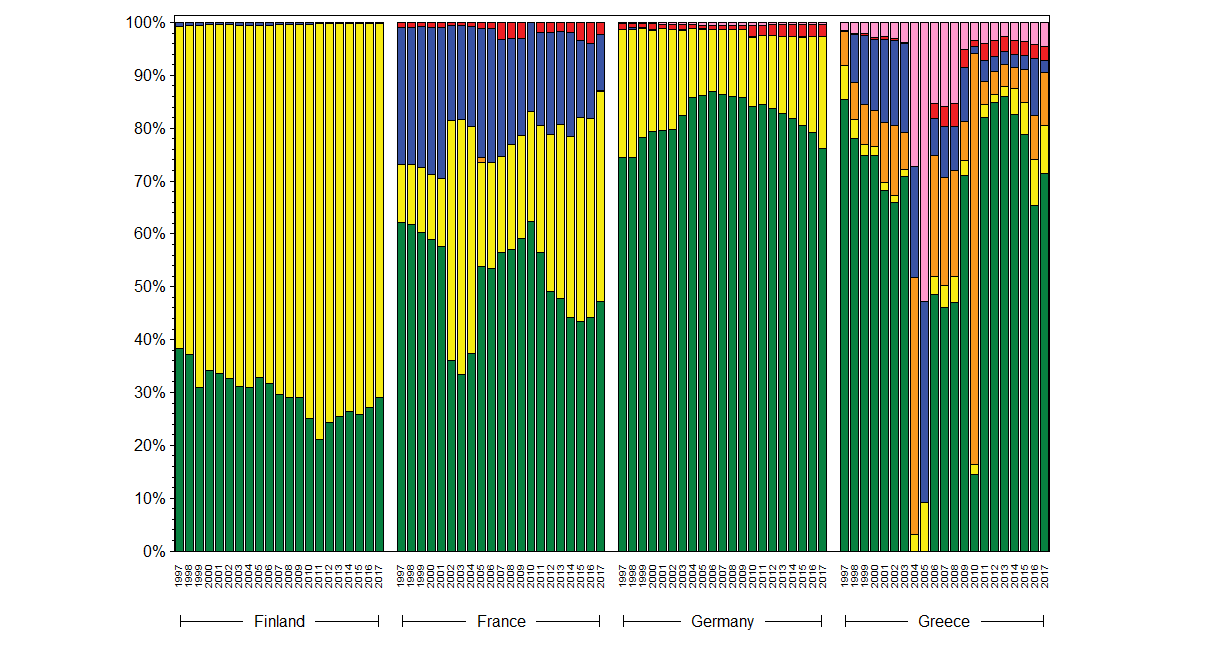

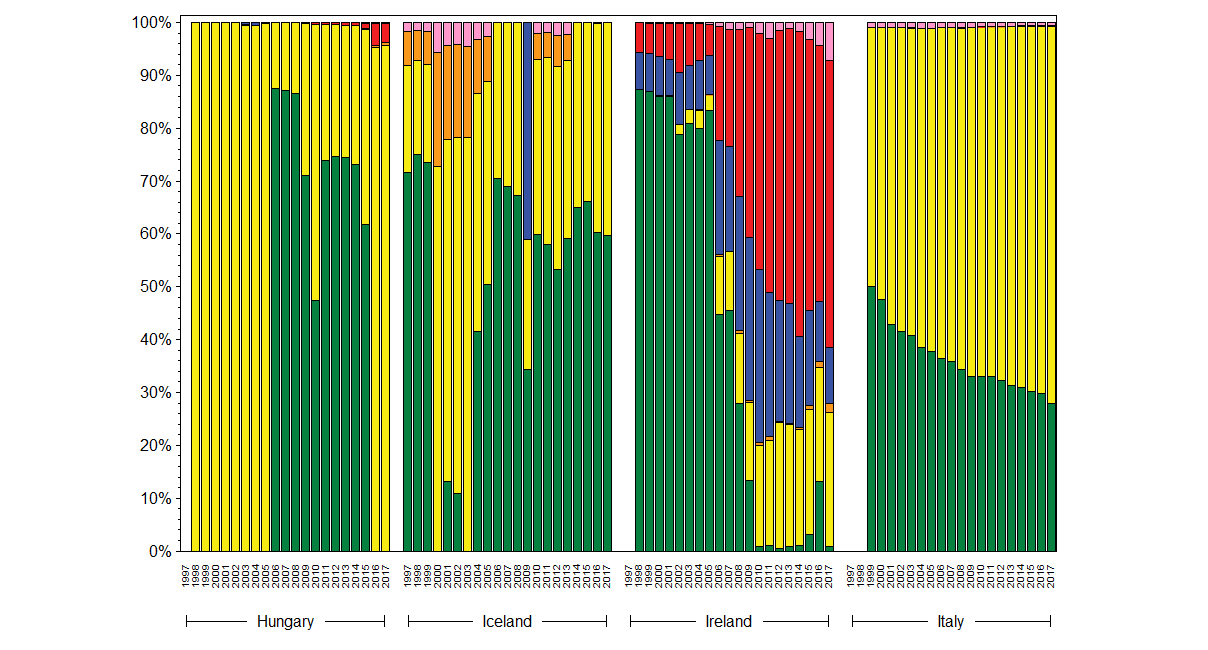


= nitrofuran derivatives (J01XE), = other antibacterials (J01XX), = imidazole derivatives (J01XD),

= steroid antibacterials (J01XC), = polymyxins (J01XB), = glycopeptide antibacterials (J01XA)

**Figure S11.** Continued


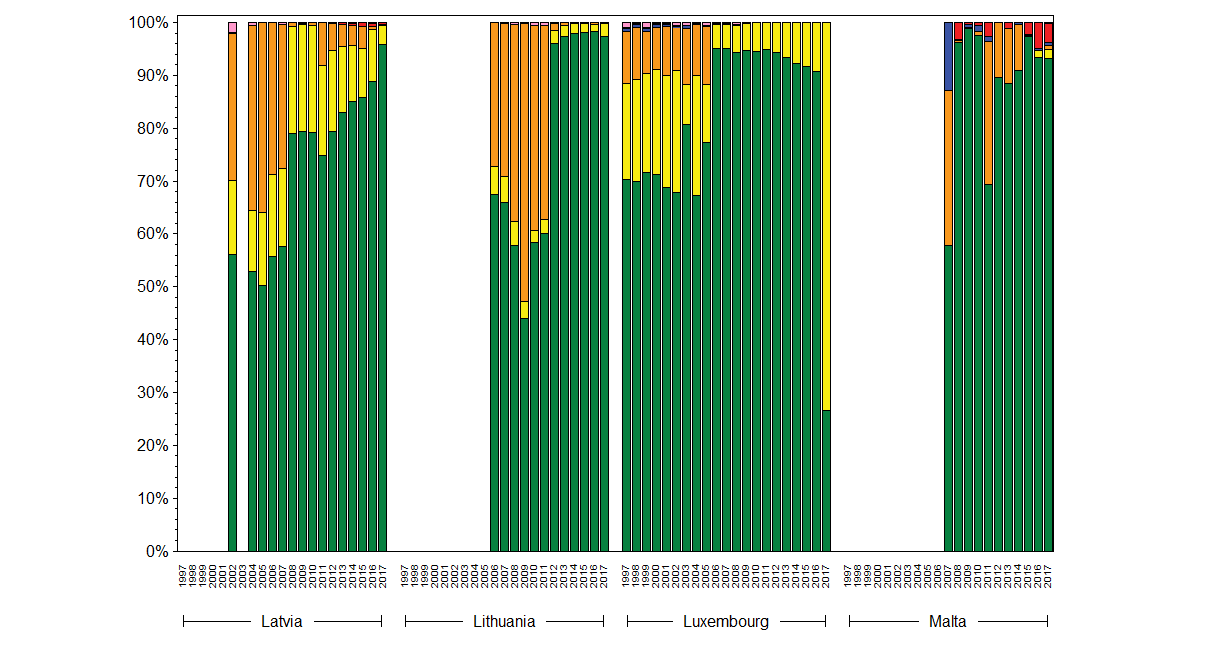

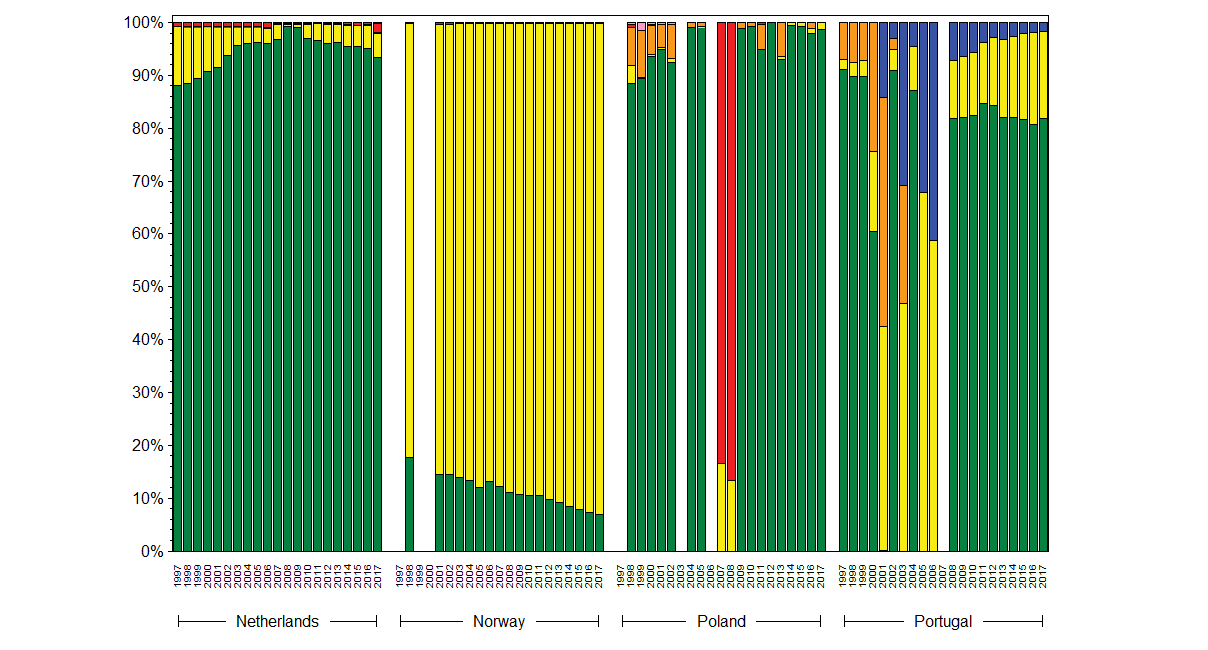


= nitrofuran derivatives (J01XE), = other antibacterials (J01XX), = imidazole derivatives (J01XD),

= steroid antibacterials (J01XC), = polymyxins (J01XB), = glycopeptide antibacterials (J01XA)

**Figure S11.** Continued


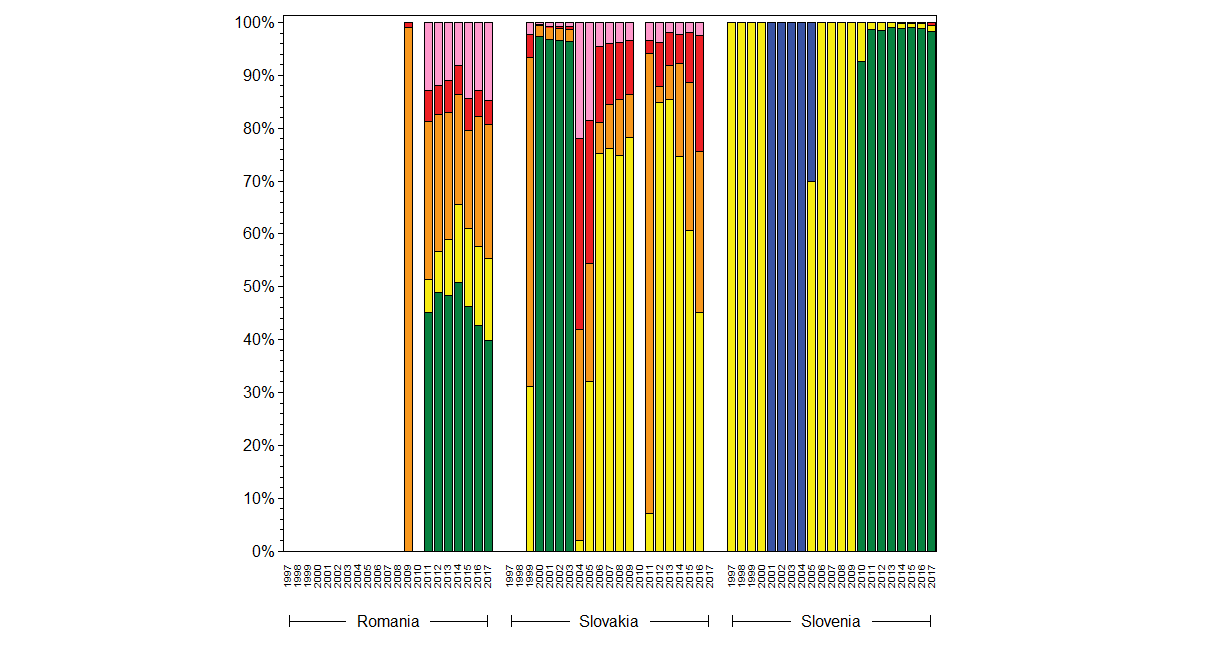

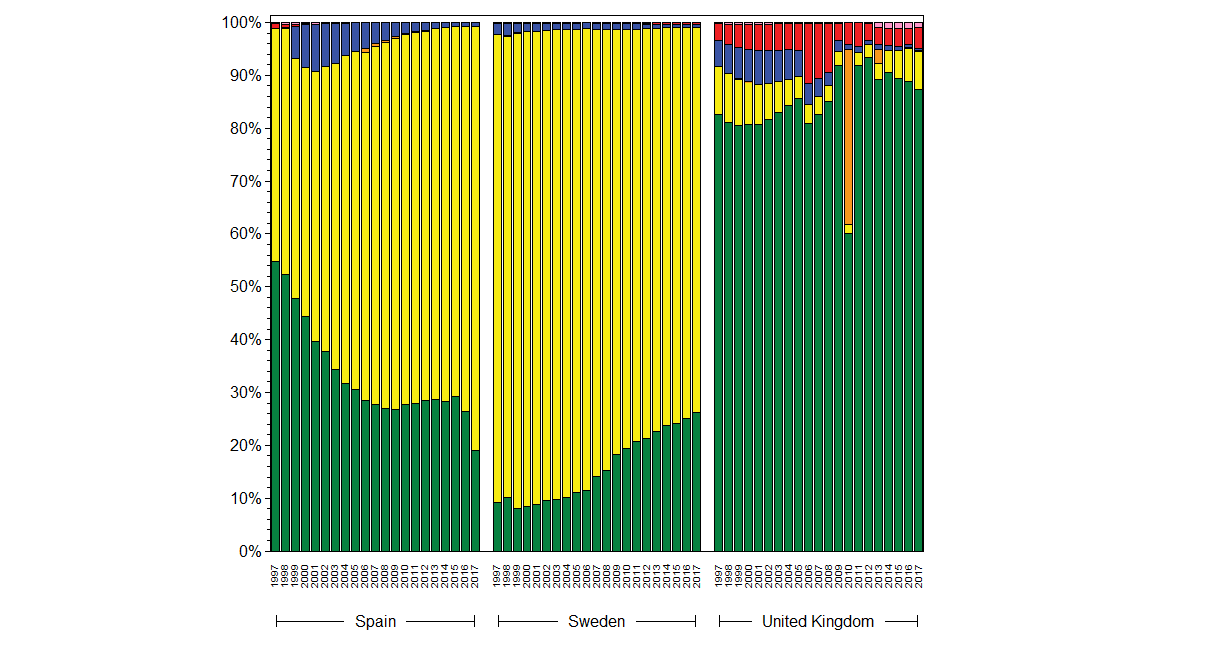


= nitrofuran derivatives (J01XE), = other antibacterials (J01XX), = imidazole derivatives (J01XD),

= steroid antibacterials (J01XC), = polymyxins (J01XB), = glycopeptide antibacterials (J01XA)

**Figure S11.** Continued
